# The global, regional, and national early-onset colorectal cancer burden and trends from 1990 to 2019: results from the Global Burden of Disease Study 2019

**DOI:** 10.1186/s12889-022-14274-7

**Published:** 2022-10-12

**Authors:** Hongfeng Pan, Zeyi Zhao, Yu Deng, Zhifang Zheng, Ying Huang, Shenghui Huang, Pan Chi

**Affiliations:** 1 Department of Colorectal Surgery Fujian Medical University Union Hospital, No.29 Xinquan Road, Fuzhou, 350001 Fujian Province China; 2 Department of General Surgery, Fujian Medical University Union Hospital, No.29 Xinquan Road, Fuzhou, 350001 Fujian Province China

**Keywords:** Early-onset colorectal cancer (EO-CRC), Incidence, Prevalence, Mortality, Disability-adjusted life years (DALYs), Global Burden of Disease (GBD)

## Abstract

**Purpose:**

The incidence of early-onset colorectal cancer (EO-CRC), which occurs in people under age 50, has been increasing annually. The aim of this study was to provide an up-to-date estimate of the global EO-CRC burden.

**Methods:**

We used Global Burden of Disease Study data and methodologies to describe changes in the EO-CRC burden from 1990 to 2019, including incidence, prevalence, mortality, and disability-adjusted life years (DALYs). The driving factors for cancer burden variation were further analyzed using decomposition analysis. Frontier analysis was used to visually demonstrate the potential for burden reduction in each country or region based on their development levels.

**Results:**

The global EO-CRC incidence more than doubled, increasing from 95,737 (95% uncertainty interval (UI): 90,838–101.042) /100,000 in 1990 to 226,782 (95% UI: 207,495–248,604) /100,000 in 2019. Additionally, related deaths increased from 50,997 (95% UI: 47,692–54,410) /100,000 to 87,014 (95% UI: 80,259–94,339) /100,000, and DALYs increased from 256,1842 (95% UI: 239,4962–2,735,823) /100,000 to 4,297,573 (95% UI: 3,965,485–4,650,790) /100,000. Regarding age-standardized rates, incidence and prevalence increased significantly, while mortality and DALYs rate were basically unchanged. Decomposition analysis showed a significant increase in DALYs in the middle sociodemographic index (SDI) quintile region, in which aging and population growth played a major driving role. Frontier analysis showed that countries or regions with a higher SDI quintile tend to have greater improvement potential.

**Conclusion:**

The current EO-CRC burden was found to be the greatest in the high-middle SDI quintile region and East Asia, which may need to adjust screening guidelines accordingly and introduce more effective interventions.

**Supplementary Information:**

The online version contains supplementary material available at 10.1186/s12889-022-14274-7.

## Introduction

In 2019, colorectal cancer became the third most common cause of cancer deaths worldwide and the second leading cause of cancer-related disability-adjusted life years (DALYs) [1]. Early-onset colorectal cancer (EO-CRC) is defined as colorectal cancer diagnosed before the age of 50 and is reported that it accounts for about 10–12% of newly diagnosed colorectal cancer [2, 3]. Currently, around 50 is also the recommended age to start most screening programs, according to analyses of the cost-effectiveness for the sustainability of healthcare systems [4]. Although in many developed countries, the incidence of colorectal cancer in people above age 50 shows a stable or slightly decreasing trend, in recent decades, the incidence of EO-CRC has shown a continuous increasing trend in many countries, such as Australia, Canada, Denmark, and nine others [3, 5]. Based on previous long-term data, this upward trend dates back to the 1990s, at the earliest. It should be noted that studies have shown that the EO-CRC mortality rate is increasing annually, though it is decreasing in people over the age of 50 [6].

However, on the one hand, the reasons for the increasing incidence of EO-CRC have not been determined, and on the other hand, there is no reasonable explanation for why EO-CRC is often advanced and poorly differentiated when diagnosed [7]. Several findings suggest that many well recognized colorectal cancer risk factors, such as lifestyle westernization and tobacco and alcohol use, among others, play important roles in EO-CRC [8–11]. Some scholars believe that geographical and sociodemographic factors such as ethnicity and income are also associated with epidemiological changes in EO-CRC. Moreover, with the expansion of screening programs and the popularization of colonoscopy, the worldwide EO-CRC burden has changed dramatically [12, 13].

In conclusion, the global EO-CRC burden is ominous, and effective use of limited health resources requires an understanding of burden variance over time and across geographical locations as well as different factors’ roles in these changes. This study aimed to provide the latest estimates of the EO-CRC burden in 204 countries and regions worldwide. The relationship between the level of sociodemographic development and the EO-CRC burden was also studied. Finally, potential improvements in the EO-CRC burden were analyzed to identify those countries or regions where more work is needed.

## Methods

### Study population and data collection

The Global Burden of Disease (GBD) Study 2019 comprehensively assessed health loss in 204 countries and territories using the latest epidemiological data sources and improved standardized methods and found that health loss was caused by 369 diseases and injuries and 87 risk factors. In the present study, we obtained and analyzed GBD Study data on EO-CRC incidence, prevalence, mortality, and DALYs (< age 50) at the global, regional, and national levels.

The sociodemographic index (SDI), a comprehensive measure of education, economic, and fertility levels, including five levels corresponding to the five SDI quintiles (i.e., low, low-middle, middle, high-middle, and high), was also used.

All data for this study are available at: http://ghdx.healthdata.org/gbd-results-tool. Data analysis was completed on April 1, 2022. The Fujian Medical University Union Hospital (FMUUH) Institutional Review Board determined that the study did not require approval because it used publicly available data. All methods were carried out in accordance with relevant guidelines and regulations.

### Statistical analysis

Previous studies have explained the methodologies of the GBD Study 2019 in detail [1, 14]. In the present study, a 95% uncertainty interval (UI) was calculated for each variable. All rates are reported per 100,000 population. All tests were two sided, and *P* values of less than .05 were considered significant.

Joinpoint regression analysis was performed to assess trends in the EO-CRC disease burden, using Joint Command Line Version 4.5.0.1, provided by the United States National Cancer Institute Surveillance Research Program. This software tracks trends in data over time and then fits the simplest model possible to the data by connecting several different line segments on a logarithmic scale. Average annual percentage changes (AAPCs) were calculated to assess trends, AAPCs is a geometrically weighted average of the different annual percentage changes from the joinpoint trend analysis, for which weights are equal to the length of each period during the specified time interval [15]. The 95% confidence interval (CI) was obtained from the linear regression model. For the AAPC value and 95% CI above zero, the corresponding age-standardized rate (ASR) showed an upward trend and vice versa. If the 95% CI of the AAPC included zero, the ASR was considered to be stable over time [16].

Decomposition analysis was used to visually demonstrate the role of the three factors driving changes in DALYs between 1990 and 2019 (i.e., aging, population, and epidemiology). Epidemiological changes refer to the underlying age and population-adjusted mortality and morbidity rates [17]. We applied frontier analysis to further assess the relationship between the EO-CRC burden and sociodemographic development. To produce a non-linear frontier, this frontier implies the lowest achievable burden determined according to development status. We used non-parametric data envelope analysis and referenced detailed descriptions in previous studies [18, 19]. The distance between the observed DALYs rate in a country and its frontier, defined as the effective difference, represents an unrealized health gain that exists based on the current level of development in the country or region. All statistical analyses and graphics were executed using R version 3.5.1.

## Results

### Overview of the Global Burden

**Fig. 1:**
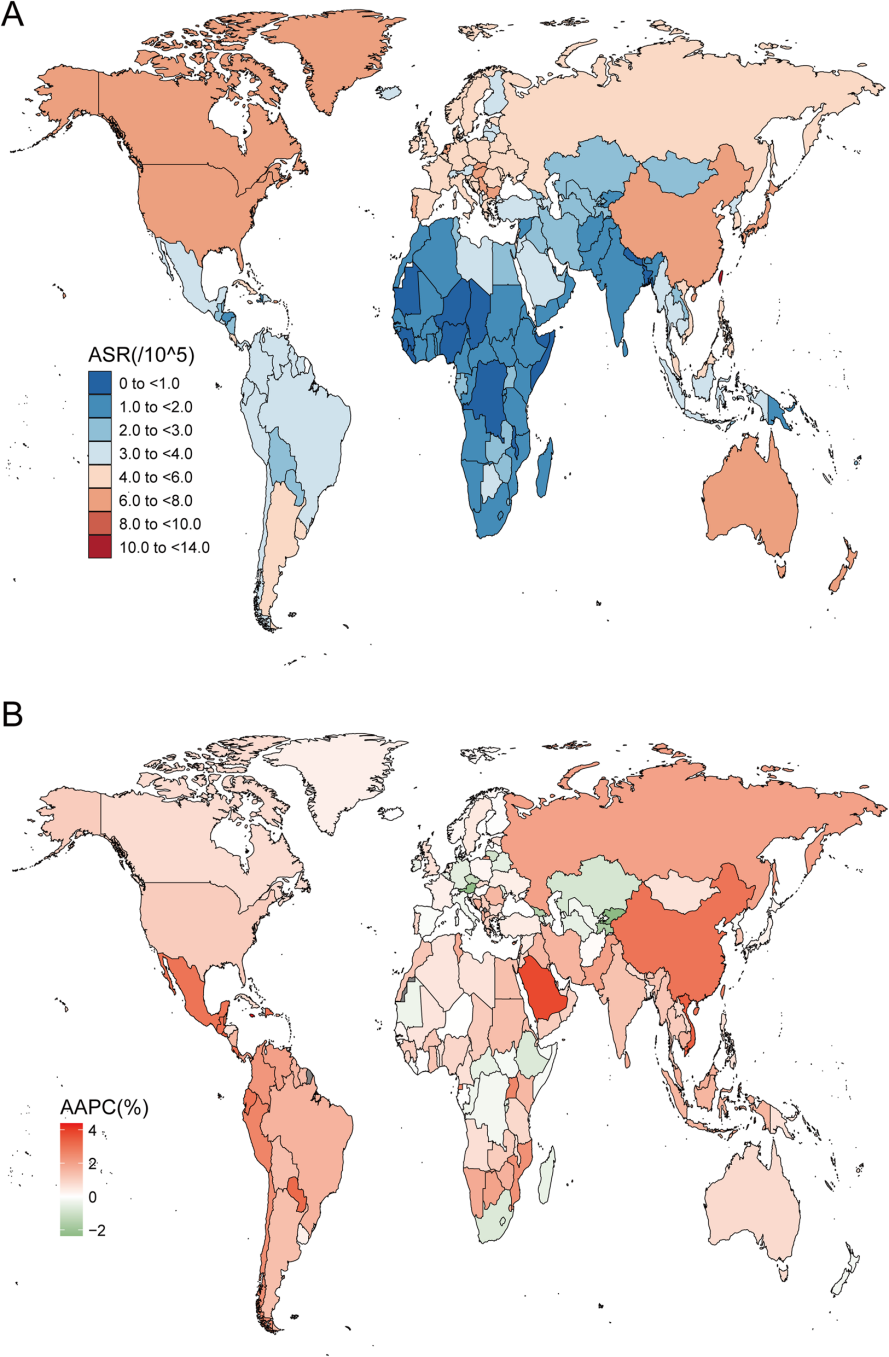
The incidence of early-onset colorectal cancer for both sexes in 204 countries and territories. **A** The age-standardized incidence of early-onset colorectal cancer in 2019; **B** The AAPC of age-standardized incidence of early-onset colorectal cancer from 1990 to 2019. AAPC, average annual percentage change

Figure 1 shows the age-standardized incidence of EO-CRC in 204 countries and territories. EO-CRC incidence more than doubled, rising from 95,737 cases (95% UI: 90,838–101,042) in 1990 to 226,782 cases (95% UI: 207,495–248,604) worldwide in 2019 (Table 1). During the same period, the age-standardized incidence rate increased from 2.95 (95% UI: 2.8–3.11) /100,000 in 1990 to 4.04 (95% UI: 3.7–4.43)/100,000 in 2019, with an average annual increase of 1.01% (95% confidence interval [1]: 0.76%–1.26%). By sex, the number of incidence cases in males 137,686 (95% UI: 121,841–155,610) was higher than that in females 89,096 (95% UI: 80,068–98,817), as was the age-standardized incidence rate, which increased faster in males than in females (males: AAPC = 1.43; 95% CI: 1.28–1.58; females: AAPC = 0.43; 95% CI: 0.27–0.59).

**Table 1:** Incidence of early-onset colorectal cancer in 1990 and 2019 for both sexes and all locations, with AAPC from 2009 and 2019

Location	1990		2019		AAPC % (95% CI) 1990–2019
	Cases (95% UI)	Age-standardized incidence per 100 000 population (95% UI)	Cases (95% UI)	Age-standardized incidence per 100 000 population (95% UI)	
Global	95,737 (90,838 to 101,042)	2.95 (2.8 to 3.11)	226,782 (207,495 to 248,604)	4.04 (3.7 to 4.43)	1.01 (0.76 to 1.26)
**Sex**					
Female	44,529 (41,296 to 48,061)	2.8 (2.6 to 3.01)	89,096 (80,068 to 98,817)	3.2 (2.87 to 3.55)	0.43 (0.27 to 0.59)
Male	51,208 (47,838 to 55,255)	3.1 (2.9 to 3.34)	137,686 (121,841 to 155,610)	4.87 (4.31 to 5.5)	1.43 (1.28 to 1.58)
**SDI**					
High SDI	32,325 (31,486 to 33,177)	5.26 (5.13 to 5.4)	47,607 (43,258 to 52,342)	6.13 (5.57 to 6.73)	0.51 (0.38 to 0.65)
High-middle SDI	27,926 (26,143 to 30,007)	3.67 (3.44 to 3.94)	69,769 (61,598 to 78,980)	5.91 (5.22 to 6.69)	1.72 (1.42 to 2.02)
Middle SDI	23,740 (21,387 to 26,304)	2.28 (2.06 to 2.53)	76,197 (67,037 to 86,572)	4.09 (3.6 to 4.65)	2.05 (1.75 to 2.34)
Low-middle SDI	8839 (7694 to 10,108)	1.43 (1.24 to 1.63)	25,242 (22,274 to 28,452)	2.11 (1.87 to 2.38)	1.42 (1.17 to 1.66)
Low SDI	2861 (2304 to 3488)	1.12 (0.91 to 1.36)	7864 (6680 to 9189)	1.32 (1.13 to 1.54)	0.61 (0.56 to 0.66)
**Region**					
High-income Asia Pacific	7981 (7574 to 8414)	5.7 (5.41 to 6.01)	9312 (7851 to 10,922)	6.08 (5.13 to 7.13)	0.11 (-0.44 to 0.65)
High-income North America	11,724 (11,271 to 12,176)	5.63 (5.41 to 5.84)	18,549 (15,830 to 21,705)	7.18 (6.13 to 8.4)	1.09 (1.01 to 1.17)
Western Europe	13,509 (12,946 to 14,087)	4.82 (4.62 to 5.02)	17,072 (14,507 to 19,975)	5.19 (4.41 to 6.06)	-0.01 (-0.22 to 0.2)
Australasia	946 (856 to 1045)	6.29 (5.69 to 6.94)	1525 (1137 to 2040)	7.07 (5.27 to 9.46)	0.61 (0.42 to 0.79)
Andean Latin America	360 (297 to 435)	1.7 (1.41 to 2.05)	1523 (1101 to 2048)	3.44 (2.49 to 4.63)	2.56 (2.14 to 2.97)
Tropical Latin America	1946 (1829 to 2069)	2.11 (1.99 to 2.24)	5780 (5325 to 6246)	3.34 (3.07 to 3.61)	1.66 (1.52 to 1.81)
Central Latin America	1488 (1409 to 1570)	1.63 (1.55 to 1.72)	5820 (4894 to 6911)	3.24 (2.72 to 3.85)	2.44 (2.29 to 2.6)
Southern Latin America	958 (865 to 1058)	2.98 (2.7 to 3.29)	2243 (1635 to 2998)	4.57 (3.33 to 6.1)	1.55 (1.32 to 1.77)
Caribbean	583 (523 to 648)	2.74 (2.46 to 3.04)	1230 (989 to 1516)	3.66 (2.94 to 4.51)	0.99 (0.94 to 1.04)
Central Europe	3872 (3677 to 4079)	4.44 (4.22 to 4.68)	4863 (4140 to 5687)	5.25 (4.47 to 6.14)	0.48 (0.24 to 0.73)
Eastern Europe	6847 (6317 to 7313)	4.57 (4.22 to 4.88)	9259 (8075 to 10,555)	5.58 (4.87 to 6.36)	0.94 (-0.44 to 2.34)
Central Asia	1311 (1216 to 1411)	3.53 (3.28 to 3.78)	1841 (1593 to 2122)	2.77 (2.39 to 3.19)	-1.09 (-1.45 to -0.72)
North Africa and Middle East	3222 (2602 to 4036)	1.81 (1.47 to 2.27)	11,201 (9520 to 13,122)	2.48 (2.11 to 2.91)	1.09 (0.96 to 1.23)
South Asia	6344 (5412 to 7390)	1.02 (0.88 to 1.19)	18,487 (15,565 to 21,669)	1.49 (1.25 to 1.75)	1.39 (1.17 to 1.6)
Southeast Asia	6201 (5178 to 7104)	2.32 (1.95 to 2.65)	19,103 (15,497 to 22,937)	3.69 (2.99 to 4.43)	1.59 (1.46 to 1.72)
East Asia	25,578 (22,075 to 29,577)	3.12 (2.69 to 3.61)	91,083 (75,766 to 107,853)	7.34 (6.12 to 8.68)	2.94 (2.7 to 3.18)
Oceania	62 (47 to 81)	1.74 (1.32 to 2.26)	167 (119 to 232)	1.97 (1.41 to 2.74)	0.41 (0.19 to 0.63)
Western Sub-Saharan Africa	843 (658 to 1055)	0.9 (0.71 to 1.13)	2657 (2103 to 3266)	1.13 (0.9 to 1.39)	0.75 (0.61 to 0.9)
Eastern Sub-Saharan Africa	1090 (853 to 1366)	1.29 (1.01 to 1.61)	3135 (2517 to 3889)	1.5 (1.21 to 1.86)	0.49 (0.41 to 0.56)
Central Sub-Saharan Africa	299 (206 to 422)	1.18 (0.82 to 1.66)	812 (556 to 1143)	1.21 (0.83 to 1.69)	0.06 (-0.06 to 0.19)
Southern Sub-Saharan Africa	575 (494 to 664)	2.04 (1.76 to 2.34)	1120 (857 to 1425)	2.06 (1.58 to 2.62)	-0.24 (-0.63 to 0.16)

*UI* Uncertainty interval, CI Confidence interval, *AAPC* Average annual percent change, *SDI* Socio-demographic index

**Fig. 2:**
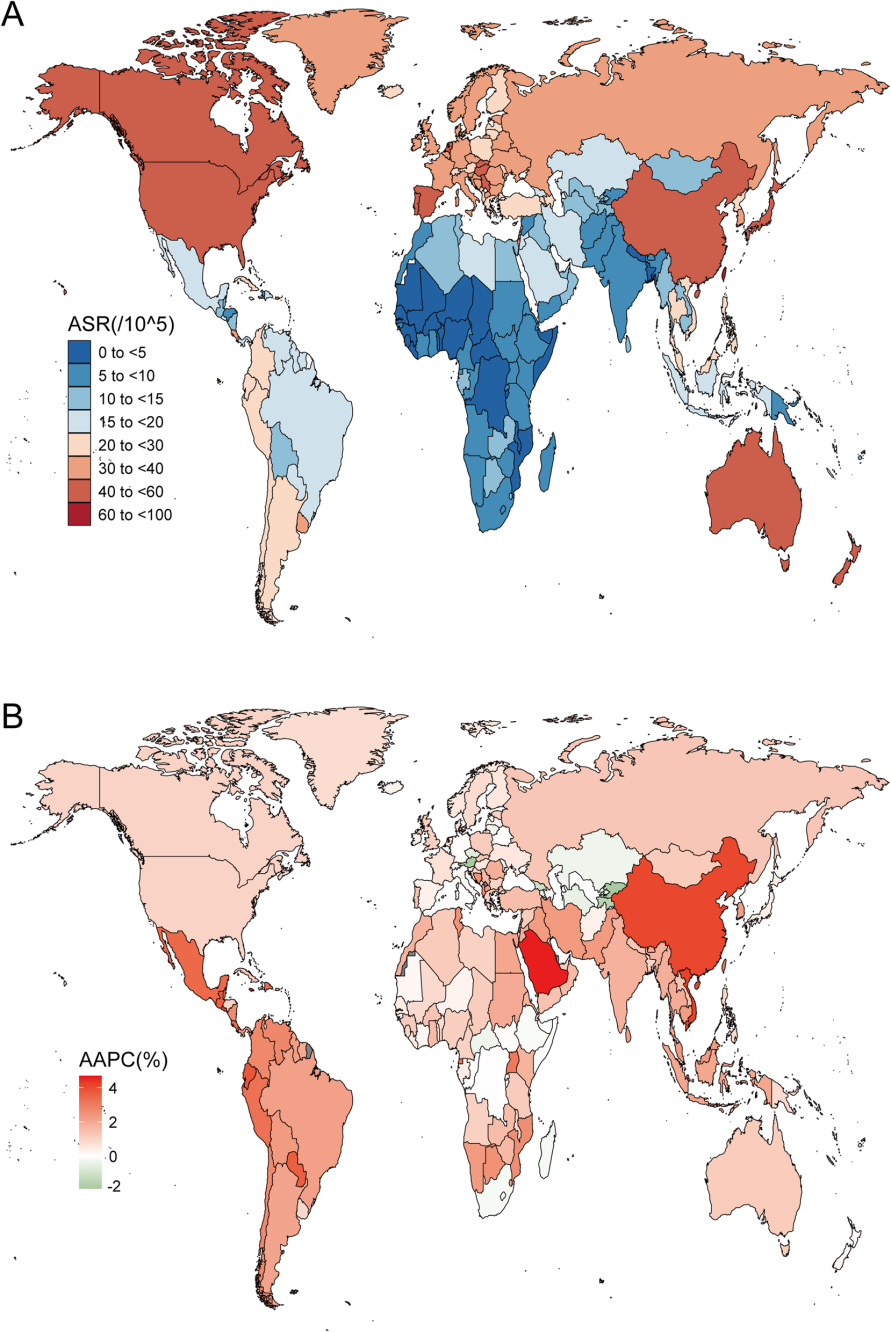
The prevalence of early-onset colorectal cancer for both sexes in 204 countries and territories. **A** The age-standardized prevalence of early-onset colorectal cancer in 2019; **B** The AAPC of age-standardized prevalence of early-onset colorectal cancer from 1990 to 2019. AAPC, average annual percentage change

Figure 2 and Table 2 show the prevalence of EO-CRC in 1990 and 2019 for all locations, as well as the AAPC for 2009 and 2019. The number of EO-CRC cases increased by 169.5% in 2019 compared to 1990. The age-standardized prevalence rate increased from 16.31 (95% UI: 15.53–17.18) /100,000 in 1990 to 25.44 (95% UI: 23.24–27.9) /100,000 in 2019, with an average annual increase of 1.43% (95% CI: 1.34%–1.52%). The EO-CRC prevalence and rate of increase are much higher in males than in females.

**Table 2:** Prevalence of early-onset colorectal cancer in 1990 and 2019 for both sexes and all locations, with AAPC from 2009 and 2019

Location	1990		2019		AAPC % (95% CI) 1990–2019
	Cases (95% UI)	Age-standardized prevalence per 100 000 population (95% UI)	Cases (95% UI)	Age-standardized prevalence per 100 000 population (95% UI)	
Global	529,611 (503,694 to 558,749)	16.31 (15.53 to 17.18)	1,427,386 (1,303,987 to 1,565,205)	25.44 (23.24 to 27.9)	1.43 (1.34 to 1.52)
**Sex**					
Female	246,208 (230,432 to 263,775)	15.46 (14.5 to 16.53)	553,203 (496,575 to 613,615)	19.87 (17.83 to 22.04)	0.87 (0.77 to 0.97)
Male	283,403 (265,564 to 303,470)	17.13 (16.09 to 18.32)	874,183 (770,140 to 991,423)	30.93 (27.26 to 35.07)	1.96 (1.88 to 2.04)
**SDI**					
High SDI	214,756 (207,303 to 223,108)	34.96 (33.75 to 36.32)	340,974 (310,697 to 373,998)	43.95 (40.06 to 48.19)	0.8 (0.76 to 0.84)
High-middle SDI	152,777 (142,896 to 164,317)	20.03 (18.75 to 21.51)	464,512 (408,620 to 527,879)	39.44 (34.69 to 44.83)	2.22 (2.08 to 2.35)
Middle SDI	113,948 (102,702 to 126,258)	10.84 (9.79 to 11.99)	465,421 (407,688 to 531,004)	25.03 (21.94 to 28.55)	2.91 (2.77 to 3.05)
Low-middle SDI	37,075 (32,378 to 42,183)	5.9 (5.17 to 6.69)	123,181 (109,234 to 138,301)	10.29 (9.13 to 11.55)	1.93 (1.71 to 2.15)
Low SDI	10,813 (8791 to 13,121)	4.15 (3.39 to 5.03)	32,707 (27,851 to 38,179)	5.43 (4.63 to 6.32)	0.92 (0.84 to 0.99)
**Region**					
High-income Asia Pacific	53,158 (49,928 to 56,609)	37.94 (35.62 to 40.43)	68,628 (57,781 to 80,329)	44.92 (37.87 to 52.56)	0.48 (0.04 to 0.92)
High-income North America	81,814 (77,945 to 85,789)	39.2 (37.36 to 41.09)	133,964 (115,141 to 155,811)	51.94 (44.67 to 60.39)	0.99 (0.69 to 1.28)
Western Europe	87,816 (83,642 to 92,408)	31.33 (29.84 to 32.98)	122,930 (105,229 to 143,136)	37.44 (32.07 to 43.56)	0.47 (0.41 to 0.54)
Australasia	6361 (5728 to 7068)	42.24 (38.05 to 46.93)	11,186 (8420 to 14,923)	51.94 (39.05 to 69.28)	1.03 (0.79 to 1.27)
Andean Latin America	1855 (1528 to 2260)	8.66 (7.18 to 10.49)	9822 (7074 to 13,299)	22.17 (15.96 to 30.02)	3.24 (2.99 to 3.49)
Tropical Latin America	9166 (8583 to 9780)	9.85 (9.23 to 10.5)	32,157 (29,553 to 34,900)	18.58 (17.07 to 20.17)	2.2 (2.14 to 2.26)
Central Latin America	7443 (7014 to 7905)	8.06 (7.61 to 8.55)	34,236 (28,862 to 40,740)	19.06 (16.07 to 22.67)	3.01 (2.94 to 3.08)
Southern Latin America	4870 (4360 to 5421)	15.14 (13.57 to 16.85)	13,306 (9786 to 17,659)	27.11 (19.94 to 35.97)	2.09 (1.94 to 2.23)
Caribbean	3087 (2760 to 3439)	14.44 (12.94 to 16.05)	7094 (5703 to 8726)	21.08 (16.94 to 25.94)	1.38 (1.3 to 1.45)
Central Europe	20,608 (19,426 to 21,852)	23.64 (22.28 to 25.07)	30,611 (26,065 to 35,706)	33.17 (28.25 to 38.69)	1.14 (0.96 to 1.32)
Eastern Europe	37,800 (34,828 to 40,408)	25.16 (23.19 to 26.88)	58,171 (50,829 to 66,416)	35.17 (30.73 to 40.14)	1.43 (0.82 to 2.04)
Central Asia	6441 (5943 to 6961)	17.09 (15.79 to 18.43)	10,080 (8735 to 11,586)	15.13 (13.12 to 17.39)	-0.65 (-1.01 to -0.3)
North Africa and Middle East	14,531 (11,807 to 18,127)	8.05 (6.56 to 10.02)	63,316 (53,915 to 74,129)	14.01 (11.93 to 16.41)	1.88 (1.75 to 2.01)
South Asia	25,424 (21,799 to 29,403)	4.03 (3.48 to 4.65)	83,288 (70,439 to 97,088)	6.69 (5.66 to 7.79)	1.79 (1.63 to 1.95)
Southeast Asia	27,997 (23,690 to 31,899)	10.37 (8.83 to 11.79)	100,248 (82,096 to 120,522)	19.38 (15.88 to 23.3)	2.14 (2.09 to 2.2)
East Asia	130,021 (112,991 to 149,447)	15.75 (13.7 to 18.08)	615,021 (512,753 to 726,748)	49.73 (41.55 to 58.68)	4.04 (3.83 to 4.25)
Oceania	271 (210 to 347)	7.47 (5.82 to 9.55)	744 (545 to 1020)	8.72 (6.39 to 11.95)	0.53 (0.46 to 0.61)
Western Sub-Saharan Africa	3319 (2622 to 4118)	3.49 (2.76 to 4.33)	11,238 (8863 to 13,819)	4.7 (3.72 to 5.77)	1.05 (0.96 to 1.14)
Eastern Sub-Saharan Africa	4044 (3199 to 5010)	4.66 (3.71 to 5.76)	12,953 (10,445 to 16,051)	6.1 (4.94 to 7.55)	0.92 (0.88 to 0.96)
Central Sub-Saharan Africa	1119 (782 to 1565)	4.29 (3.02 to 5.98)	3264 (2278 to 4578)	4.77 (3.34 to 6.67)	0.36 (0.31 to 0.4)
Southern Sub-Saharan Africa	2466 (2121 to 2845)	8.63 (7.46 to 9.9)	5130 (3942 to 6498)	9.39 (7.25 to 11.87)	0.28 (0.08 to 0.48)

*UI* Uncertainty interval, *CI* Confidence interval, *AAPC* Average annual percent change, *SDI* Socio-demographic index

**Fig. 3:**
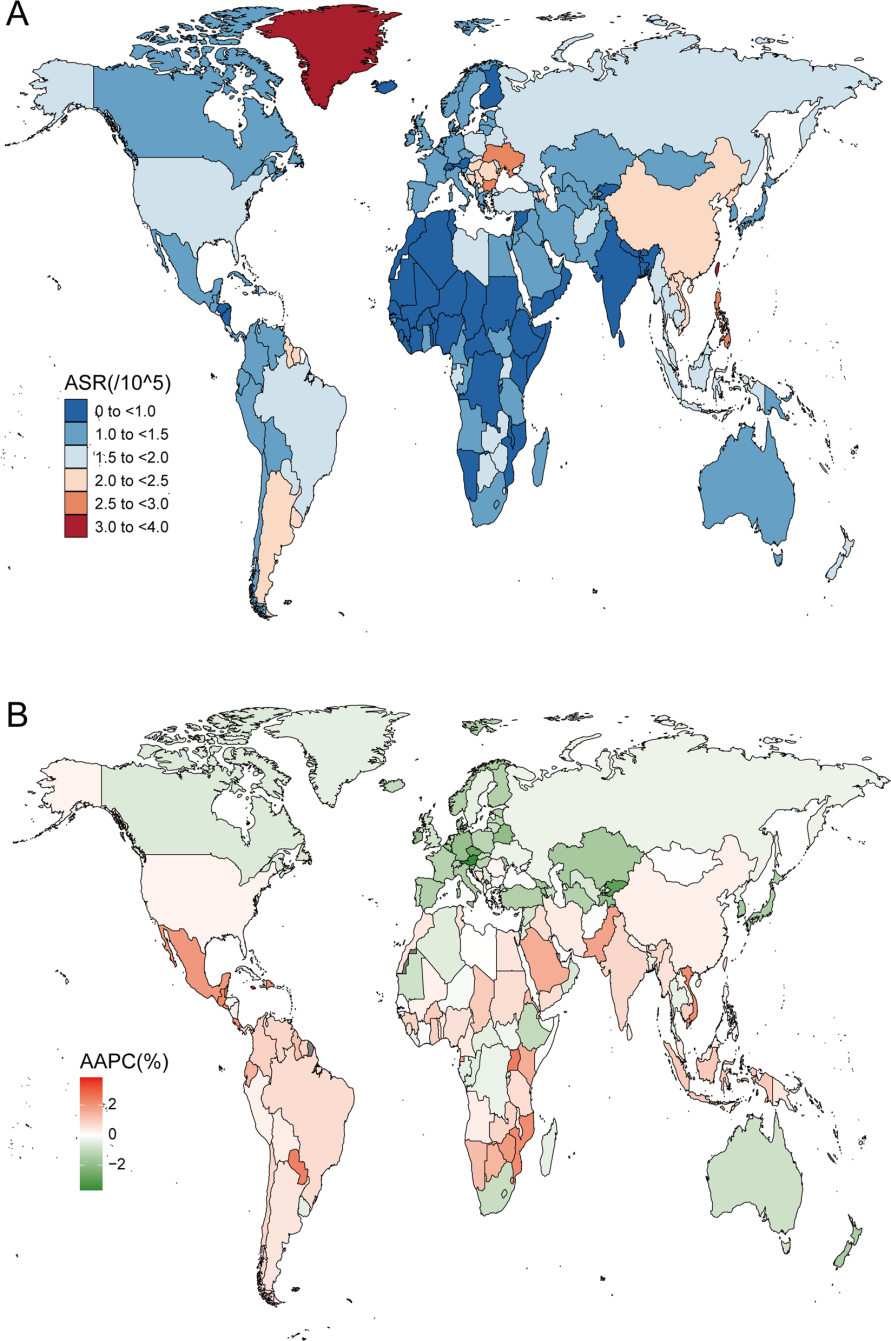
The mortality of early-onset colorectal cancer for both sexes in 204 countries and territories. **A** The age-standardized mortality of early-onset colorectal cancer in 2019; **B** The AAPC of age-standardized mortality of early-onset colorectal cancer from 1990 to 2019. AAPC, average annual percentage change

From 1990 to 2019, the number of worldwide deaths associated with EO-CRC increased from 50,997 (95% UI: 47,692–54,410) to 87,014 (95% UI: 80,259–94339; Table 3). However, the age-standardized mortality rate essentially remained flat over the same period (AAPC = 0.07%; 95% CI: -0.16–0.02). The worldwide EO-CRC age-standardized mortality rate and its changes are shown in Fig. 3. Regarding gender, the number of male deaths was 51,243 (95% UI: 45,960–56957) compared with 35,771 (95% UI: 32,388–39350) for females. The age-standardized death rate followed a similar trend. Evidently, men’s age-standardized mortality rate increased slightly each year (AAPC = 0.23; 95% CI: 0.07–0.38), but this was not the case for women (AAPC = -0.57; 95% CI: -0.71 – -0.44).

**Table 3:** Mortality of early-onset colorectal cancer in 1990 and 2019 for both sexes and all locations, with AAPC from 2009 and 2019

Location	1990		2019		AAPC % (95% CI) 1990–2019
	Cases (95% UI)	Age-standardized mortality per 100 000 population (95% UI)	Cases (95% UI)	Age-standardized mortality per 100 000 population (95% UI)	
Global	50,997 (47,692 to 54,410)	1.57 (1.47 to 1.68)	87,014 (80,259 to 94,339)	1.55 (1.43 to 1.68)	-0.07 (-0.16 to 0.02)
**Sex**					
Female	23,714 (21,653 to 26,102)	1.49 (1.37 to 1.64)	35,771 (32,388 to 39,350)	1.28 (1.16 to 1.41)	-0.57 (-0.71 to -0.44)
Male	27,284 (25,066 to 29,985)	1.65 (1.52 to 1.82)	51,243 (45,960 to 56,957)	1.81 (1.62 to 2.01)	0.23 (0.07 to 0.38)
**SDI**					
High SDI	11,590 (11,316 to 11,853)	1.89 (1.84 to 1.93)	12,045 (11,467 to 12,676)	1.54 (1.47 to 1.62)	-0.76 (-0.93 to -0.59)
High-middle SDI	15,002 (13,930 to 16,180)	1.98 (1.84 to 2.13)	22,235 (20,123 to 24,677)	1.87 (1.69 to 2.08)	-0.27 (-0.64 to 0.1)
Middle SDI	15,520 (13,872 to 17,221)	1.51 (1.35 to 1.68)	31,372 (28,038 to 34,858)	1.68 (1.5 to 1.87)	0.33 (0.24 to 0.42)
Low-middle SDI	6579 (5715 to 7534)	1.08 (0.94 to 1.23)	15,526 (13,629 to 17,625)	1.3 (1.15 to 1.48)	0.59 (0.4 to 0.79)
Low SDI	2282 (1849 to 2783)	0.91 (0.74 to 1.11)	5787 (4931 to 6785)	0.99 (0.84 to 1.15)	0.29 (0.25 to 0.34)
**Region**					
High-income Asia Pacific	2848 (2745 to 2954)	2.04 (1.96 to 2.12)	2060 (1906 to 2218)	1.34 (1.23 to 1.44)	-1.55 (-1.86 to -1.24)
High-income North America	3620 (3496 to 3741)	1.74 (1.68 to 1.8)	4555 (4331 to 4806)	1.75 (1.67 to 1.85)	0.15 (0.04 to 0.27)
Western Europe	5103 (4945 to 5271)	1.82 (1.76 to 1.88)	4258 (3998 to 4528)	1.28 (1.2 to 1.36)	-1.17 (-1.48 to -0.85)
Australasia	322 (297 to 349)	2.14 (1.97 to 2.32)	338 (288 to 396)	1.55 (1.32 to 1.83)	-1.01 (-1.22 to -0.81)
Andean Latin America	208 (174 to 249)	1 (0.84 to 1.19)	531 (393 to 700)	1.2 (0.89 to 1.58)	0.63 (0.35 to 0.9)
Tropical Latin America	1255 (1185 to 1331)	1.38 (1.3 to 1.46)	2868 (2660 to 3089)	1.65 (1.53 to 1.78)	0.7 (0.56 to 0.84)
Central Latin America	894 (850 to 941)	1 (0.95 to 1.05)	2579 (2181 to 3048)	1.44 (1.21 to 1.7)	1.25 (1.18 to 1.32)
Southern Latin America	589 (536 to 646)	1.84 (1.67 to 2.02)	1026 (880 to 1190)	2.08 (1.78 to 2.42)	0.46 (0.27 to 0.66)
Caribbean	318 (285 to 355)	1.5 (1.35 to 1.68)	566 (451 to 706)	1.68 (1.34 to 2.1)	0.39 (0.08 to 0.71)
Central Europe	2125 (2031 to 2225)	2.44 (2.33 to 2.55)	1866 (1590 to 2161)	2 (1.7 to 2.31)	-0.82 (-1.12 to -0.53)
Eastern Europe	3531 (3259 to 3770)	2.36 (2.18 to 2.52)	3465 (3026 to 3955)	2.08 (1.81 to 2.37)	-0.43 (-2.09 to 1.26)
Central Asia	813 (756 to 874)	2.23 (2.07 to 2.39)	987 (854 to 1137)	1.48 (1.28 to 1.71)	-1.63 (-2 to -1.26)
North Africa and Middle East	2236 (1805 to 2802)	1.28 (1.04 to 1.6)	5529 (4689 to 6523)	1.23 (1.04 to 1.45)	-0.08 (-0.25 to 0.09)
South Asia	4962 (4248 to 5781)	0.81 (0.69 to 0.94)	12,558 (10,615 to 14,794)	1.02 (0.86 to 1.2)	0.88 (0.66 to 1.09)
Southeast Asia	4303 (3606 to 4940)	1.63 (1.37 to 1.87)	10,616 (8646 to 12,691)	2.05 (1.67 to 2.45)	0.8 (0.69 to 0.91)
East Asia	15,658 (13,393 to 18,168)	1.93 (1.65 to 2.24)	27,495 (22,938 to 32,625)	2.19 (1.83 to 2.59)	0.29 (0.05 to 0.53)
Oceania	43 (33 to 57)	1.24 (0.93 to 1.62)	113 (80 to 158)	1.34 (0.96 to 1.87)	0.29 (0.13 to 0.45)
Western Sub-Saharan Africa	656 (514 to 823)	0.72 (0.56 to 0.9)	1922 (1518 to 2394)	0.83 (0.66 to 1.03)	0.53 (0.38 to 0.67)
Eastern Sub-Saharan Africa	866 (672 to 1086)	1.04 (0.81 to 1.3)	2327 (1877 to 2883)	1.13 (0.91 to 1.4)	0.25 (0.16 to 0.35)
Central Sub-Saharan Africa	237 (167 to 330)	0.95 (0.68 to 1.32)	613 (421 to 866)	0.92 (0.64 to 1.3)	-0.12 (-0.26 to 0.02)
Southern Sub-Saharan Africa	412 (355 to 474)	1.48 (1.28 to 1.7)	743 (574 to 937)	1.38 (1.07 to 1.73)	-0.49 (-0.88 to -0.09)

*UI* Uncertainty interval, *CI* Confidence interval, *AAPC* Average annual percent change, *SDI* Socio-demographic index

**Fig. 4:**
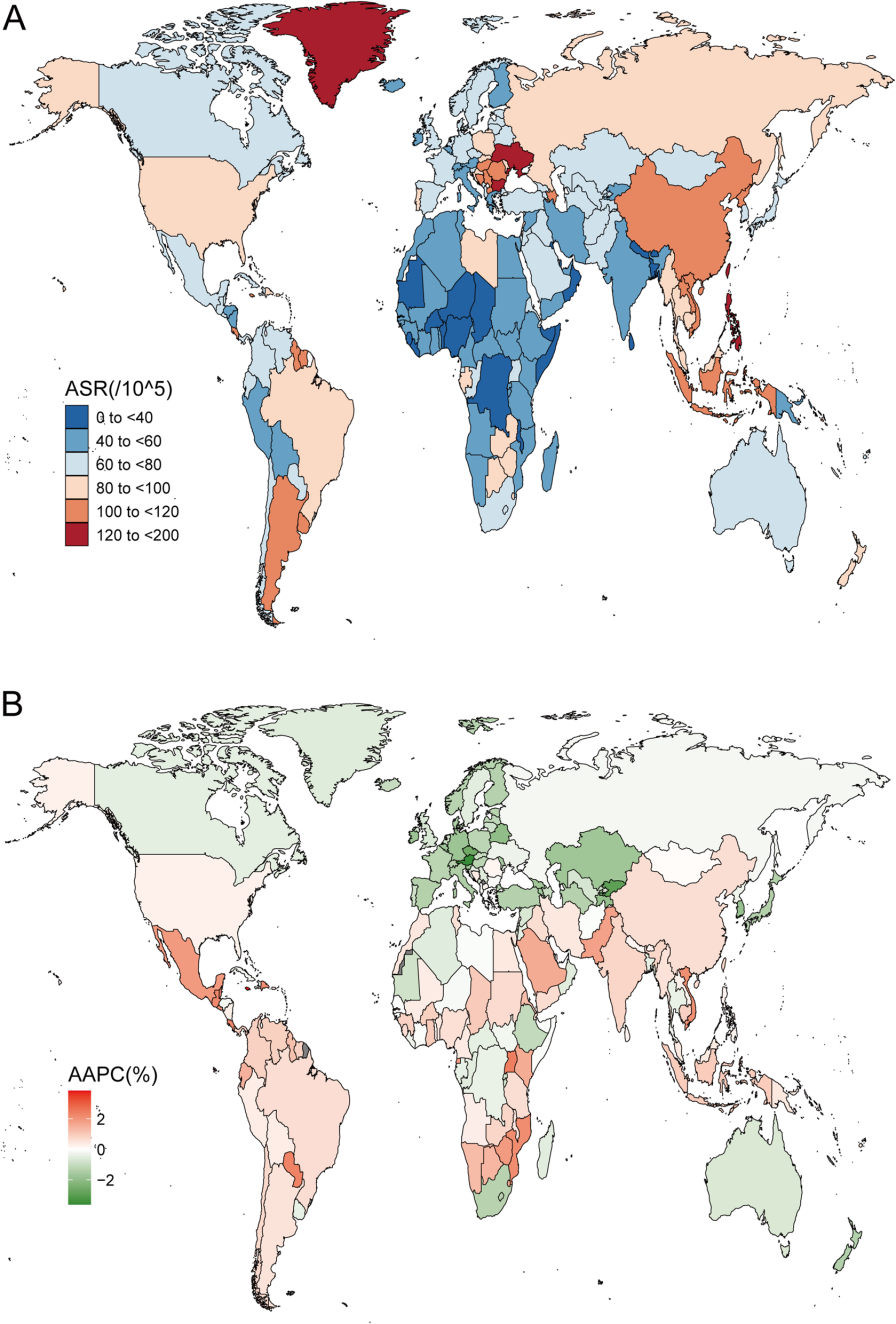
The DALYs of early-onset colorectal cancer for both sexes in 204 countries and territories. **A** The age-standardized DALYs of early-onset colorectal cancer in 2019; **B** The AAPC of age-standardized DALYs of early-onset colorectal cancer from 1990 to 2019. ASR, age-standardized rate; AAPC, average annual percentage change; DALYs, Disability-Adjusted Life Years

Worldwide DALYs caused by EO-CRC increased from 2,561,842 (95% UI: 239,4962–2735823) in 1990 to 4,297,573 (95% UI: 3,965,485–4,650,790) in 2019 (Table 4). The age-standardized DALYs rate was 77.7 (95% UI: 72.76–82.85) /100,000 in 1990 and 76.86 (95% UI: 70.91–83.19) /100,000 in 2019 (Fig. 4A) and has essentially been flat over the past 30 years (AAPC = -0.08; 95% CI: -0.16–0.01; Fig. 4B). Additionally, the number of DALYs in males was significantly higher than that in females, with an opposite trend (males: AAPC = 0.24; 95% CI: 0.09–0.39; females: AAPC = -0.55; 95% CI: -0.68 – -0.42).

**Table 4:** DALY of early-onset colorectal cancer in 1990 and 2019 for both sexes and all locations, with AAPC from 2009 and 2019

Location	1990		2019		AAPC % (95% CI) 1990–2019
	Cases (95% UI)	Age-standardized DALY per 100 000 population (95% UI)	Cases (95% UI)	Age-standardized DALY per 100 000 population (95% UI)	
Global	2,561,842 (2,394,962 to 2,735,823)	77.7 (72.76 to 82.85)	4,297,573 (3,965,485 to 4,650,790)	76.86 (70.91 to 83.19)	-0.08 (-0.16 to 0.01)
**Sex**					
Female	1,189,993 (1,084,567 to 1,310,035)	73.51 (67.19 to 80.77)	1,762,817 (1,596,957 to 1,938,989)	63.58 (57.57 to 69.95)	-0.55 (-0.68 to -0.42)
Male	1,371,849 (1,260,775 to 1,508,695)	81.74 (75.27 to 89.77)	2,534,756 (2,278,256 to 2,814,579)	89.95 (80.85 to 99.86)	0.24 (0.09 to 0.39)
**SDI**					
High SDI	565,844 (551,158 to 580,209)	92.23 (89.82 to 94.58)	591,574 (562,033 to 624,366)	76.85 (72.97 to 81.16)	-0.68 (-0.85 to -0.52)
High-middle SDI	750,711 (696,355 to 811,081)	97.6 (90.65 to 105.32)	1,098,600 (994,304 to 1,214,332)	93.91 (84.98 to 103.82)	-0.23 (-0.6 to 0.13)
Middle SDI	796,532 (712,556 to 884,382)	75.51 (67.64 to 83.73)	1,550,467 (1,388,571 to 1,717,229)	83.8 (75.05 to 92.8)	0.32 (0.22 to 0.42)
Low-middle SDI	333,162 (288,215 to 382,422)	52.98 (46.04 to 60.67)	766,284 (672,536 to 870,105)	63.92 (56.12 to 72.57)	0.72 (0.6 to 0.85)
Low SDI	114,328 (92,257 to 139,751)	44.09 (35.75 to 53.74)	288,192 (245,104 to 338,714)	47.83 (40.77 to 56.1)	0.31 (0.26 to 0.35)
**Region**	138,575 (133,000 to 144,275)	100.28 (96.09 to 104.6)	100,788 (93,094 to 108,937)	67.31 (62.02 to 72.96)	-1.46 (-1.79 to -1.13)
High-income Asia Pacific					
High-income North America	178,942 (172,223 to 185,314)	85.68 (82.47 to 88.73)	224,187 (212,140 to 237,605)	87.32 (82.57 to 92.65)	0.21 (0.09 to 0.32)
Western Europe	246,823 (238,646 to 255,303)	88.27 (85.31 to 91.33)	207,340 (193,916 to 221,500)	63.64 (59.48 to 68.06)	-1.08 (-1.39 to -0.77)
Australasia	15,611 (14,315 to 16,985)	103.71 (95.1 to 112.84)	16,851 (14,264 to 19,864)	78.69 (66.43 to 92.97)	-0.81 (-0.94 to -0.68)
Andean Latin America	10,727 (8924 to 12,898)	49.86 (41.7 to 59.65)	27,087 (20,040 to 35,742)	61.07 (45.18 to 80.59)	0.69 (0.42 to 0.96)
Tropical Latin America	63,639 (59,960 to 67,593)	68.09 (64.23 to 72.22)	140,322 (130,057 to 151,074)	81.18 (75.22 to 87.43)	0.68 (0.54 to 0.82)
Central Latin America	46,399 (44,064 to 48,878)	49.98 (47.55 to 52.56)	129,267 (109,626 to 152,485)	71.99 (61.05 to 84.91)	1.26 (1.19 to 1.33)
Southern Latin America	28,721 (26,077 to 31,609)	89.25 (81.09 to 98.15)	50,371 (43,173 to 58,593)	102.82 (88.05 to 119.71)	0.54 (0.32 to 0.75)
Caribbean	15,941 (14,207 to 17,883)	74.04 (66.22 to 82.81)	27,802 (22,083 to 34,737)	83.05 (65.9 to 103.85)	0.41 (0.1 to 0.71)
Central Europe	102,738 (97,982 to 107,802)	117.71 (112.23 to 123.55)	88,960 (75,933 to 103,094)	97 (82.83 to 112.41)	-0.79 (-1.1 to -0.47)
Eastern Europe	173,230 (159,657 to 185,203)	114.92 (105.98 to 122.84)	168,658 (147,831 to 192,396)	102.48 (89.82 to 116.92)	-0.37 (-1.97 to 1.27)
Central Asia	42,467 (39,423 to 45,696)	111.7 (103.94 to 119.88)	49,744 (43,004 to 57,382)	74.4 (64.32 to 85.82)	-1.65 (-2.03 to -1.28)
North Africa and Middle East	113,331 (91,128 to 142,353)	62.75 (50.66 to 78.62)	273,698 (231,829 to 322,390)	60.49 (51.23 to 71.25)	-0.07 (-0.23 to 0.1)
South Asia	248,721 (211,957 to 290,817)	39.47 (33.83 to 45.97)	616,964 (520,893 to 727,009)	49.49 (41.81 to 58.31)	0.86 (0.66 to 1.07)
Southeast Asia	219,226 (183,062 to 252,017)	80.7 (67.78 to 92.54)	522,972 (427,068 to 624,755)	101.43 (82.86 to 121.14)	0.81 (0.7 to 0.92)
East Asia	805,205 (689,571 to 932,724)	97.01 (83.09 to 112.38)	1,366,228 (1,152,737 to 1,609,615)	111.28 (94.03 to 130.8)	0.61 (0.26 to 0.97)
Oceania	2224 (1670 to 2942)	61.24 (46.13 to 80.7)	5723 (4069 to 7991)	66.91 (47.56 to 93.39)	0.3 (0.13 to 0.48)
Western Sub-Saharan Africa	33,034 (25,850 to 41,448)	34.89 (27.32 to 43.71)	97,086 (76,476 to 121,008)	40.6 (32.07 to 50.55)	0.55 (0.4 to 0.7)
Eastern Sub-Saharan Africa	43,415 (33,586 to 54,521)	50.41 (39.23 to 63.13)	115,961 (93,351 to 143,938)	54.66 (44.11 to 67.77)	0.25 (0.18 to 0.31)
Central Sub-Saharan Africa	11,965 (8394 to 16,739)	46.31 (32.77 to 64.43)	30,537 (20,886 to 43,251)	44.71 (30.65 to 63.11)	-0.13 (-0.24 to -0.02)
Southern Sub-Saharan Africa	20,906 (17,964 to 24,174)	73.03 (63.04 to 83.99)	37,028 (28,480 to 46,855)	67.63 (52.21 to 85.35)	-0.56 (-0.98 to -0.15)

*UI* Uncertainty interval, *CI* Confidence interval, *AAPC* Average annual percent change, *SDI* Socio-demographic index

### EO-CRC Burden by SDI Quintile

**Fig. 5:**
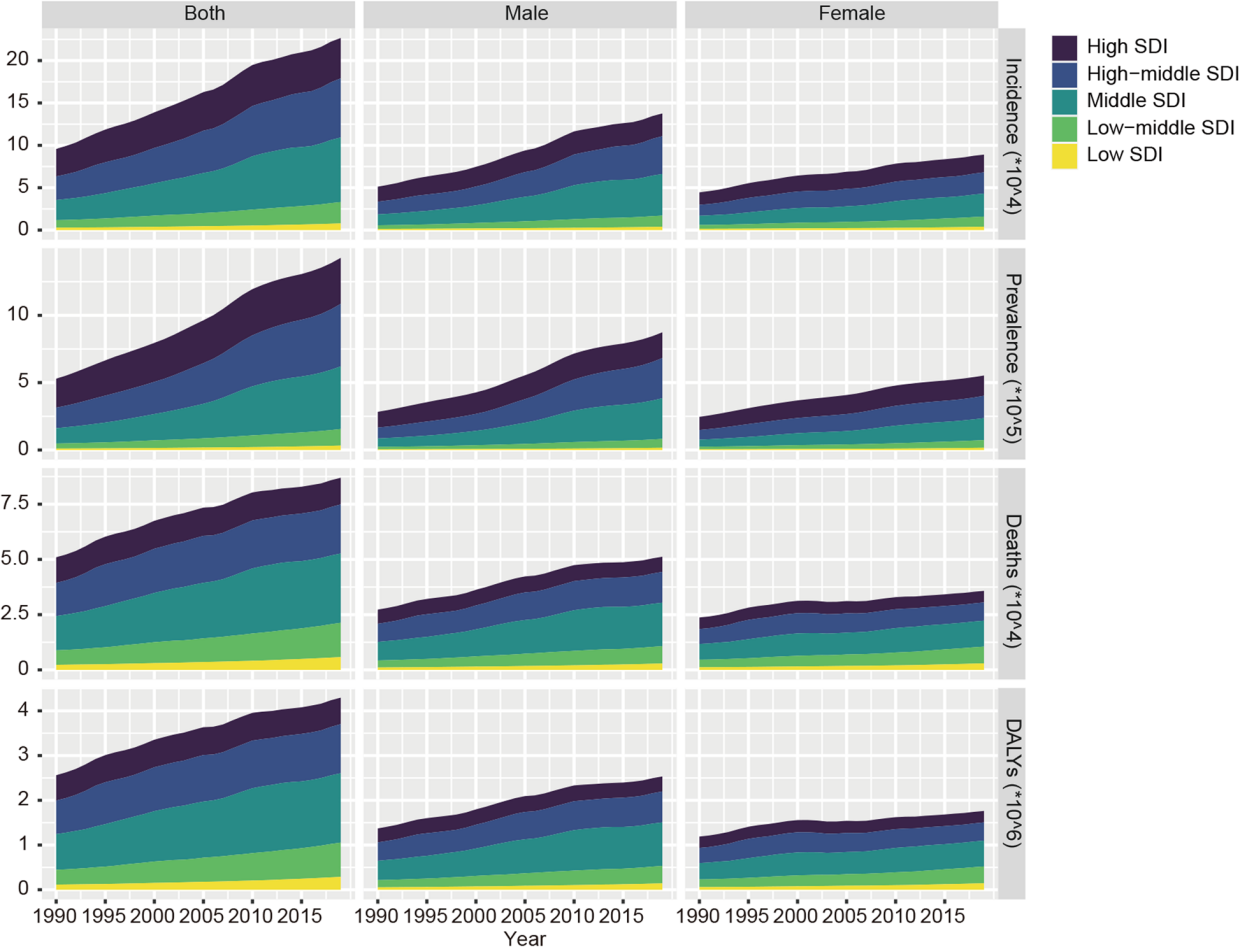
The number of incidence, prevalence, deaths and DALYs due to early-onset colorectal cancer grouped by SDI quintiles for different sexes from 1990 to 2019. SDI: Socio-demographic index; DALYs, Disability-Adjusted Life Years

**Fig.6:**
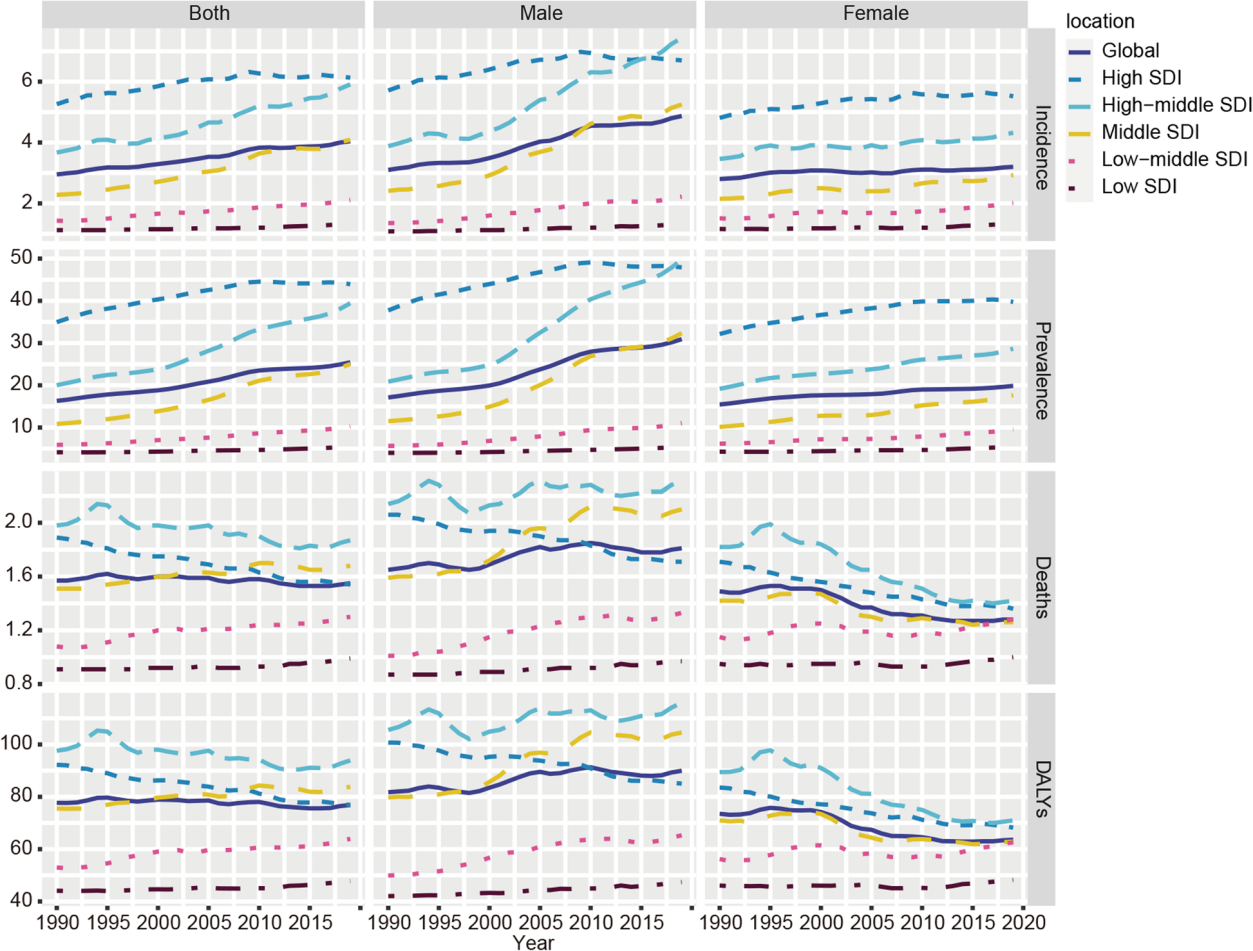
Age-standardized incidence, prevalence, mortality and DALYs per 100 000 people of early-onset colorectal cancer grouped by SDI quintiles for different sexes from 1990 to 2019. SDI: Socio-demographic index; DALYs, Disability-Adjusted Life Years

Figure 5 shows EO-CRC incidence, prevalence, deaths, and DALYs grouped by SDI quintiles from 1990 to 2019. The middle SDI region had the most incidence cases (76,197; 95% UI: 67,037–86,572) and the most death cases (31,372; 95% UI: 28,038–34,858) and DALYs (155,0467; 95% UI: 138,8571–1,717,229). Regarding ASR, higher SDI quintiles tend to have higher age-standardized incidence rates, with the highest found in the high SDI quintiles region (6.13; 95% UI: 5.57–6.73/100, 000) (Fig. 6, Table 1). EO-CRC prevalence and variance across SDI quintiles are shown in Table 2 and Fig. 6. The highest age-standardized mortality rate was found in the high-middle SDI region (1.87; 95% UI: 1.69–2.08/100, 000). Regarding age-standardized DALYs, the high-middle SDI region ranked first (93.91; 95% UI: 84.98–103.82/100,000). Additionally, the EO-CRC burden was generally greater for males than for females in every SDI quintile (Fig. 6).

### EO-CRC Burden in 21 GBD Regions

Among 21 GBD regions by SDI in 2019, East Asia had the highest EO-CRC incidence (91,083; 95% UI: 75,766–107,853), while high-income North America had the highest age-standardized incidence rate (7.18, 95% UI: 6.13–8.4/100,000; Table 1, Figure S1A). East Asia saw the most rapid increase in incidence rates (AAPC = 2.94; 95% CI: 2.7–3.18). Regarding the age-standardized prevalence rate, high-income North America (51.94; 95% UI: 44.67–60.39) and Australasia (51.94; 95% UI: 39.05–69.28) ranked first (Table 2, Figure S2A). East Asia had the most deaths (27,495; 95% UI: 22,938–32,625) and the highest age-standardized mortality rate (2.19; 95% UI: 1.83–2.59/100,000; Table 3, Figure S3A) as well as the highest age-standardized DALYs rate (111.28, 95% UI: 94.03–130.8; Table 4, Figure S4A). Central Latin America has the fastest growth rate in DALYs (AAPC = 1.26; 95% CI: 1.19–1.33).

### EO-CRC Burden by Country or Region

In 2019, China had the most incidence cases (87,551; 95% UI: 72,074–104,615; Table S1), the most prevalence cases (591,944; 95% UI: 489,497–705,006; Table S2), the most death cases (26,320; 95% UI: 21,827–31,571; Table S3) and the most DALYs numbers (1,308,591; 95% UI: 1,096,385–1,556,940; Table S4; Figure S4B) in the world. Regarding ASR, the Chinese province of Taiwan had the highest incidence (13.19; 95% UI: 9.26–18.38/100,000; Figure S1B), prevalence (91.46; 95% UI: 64.15–127.86/100, 000; Figure S2B), mortality (3.46; 95% UI: 2.48–4.7/100,000; Figure S3B), and DALYs rates (173.31; 95% UI: 124.55–236.4/100,000; Fig. 4B) in the world. Additionally, Jamaica has shown the fastest cancer burden increase.

### Decomposition analysis of age-standardized DALYs rates

**Fig. 7:**
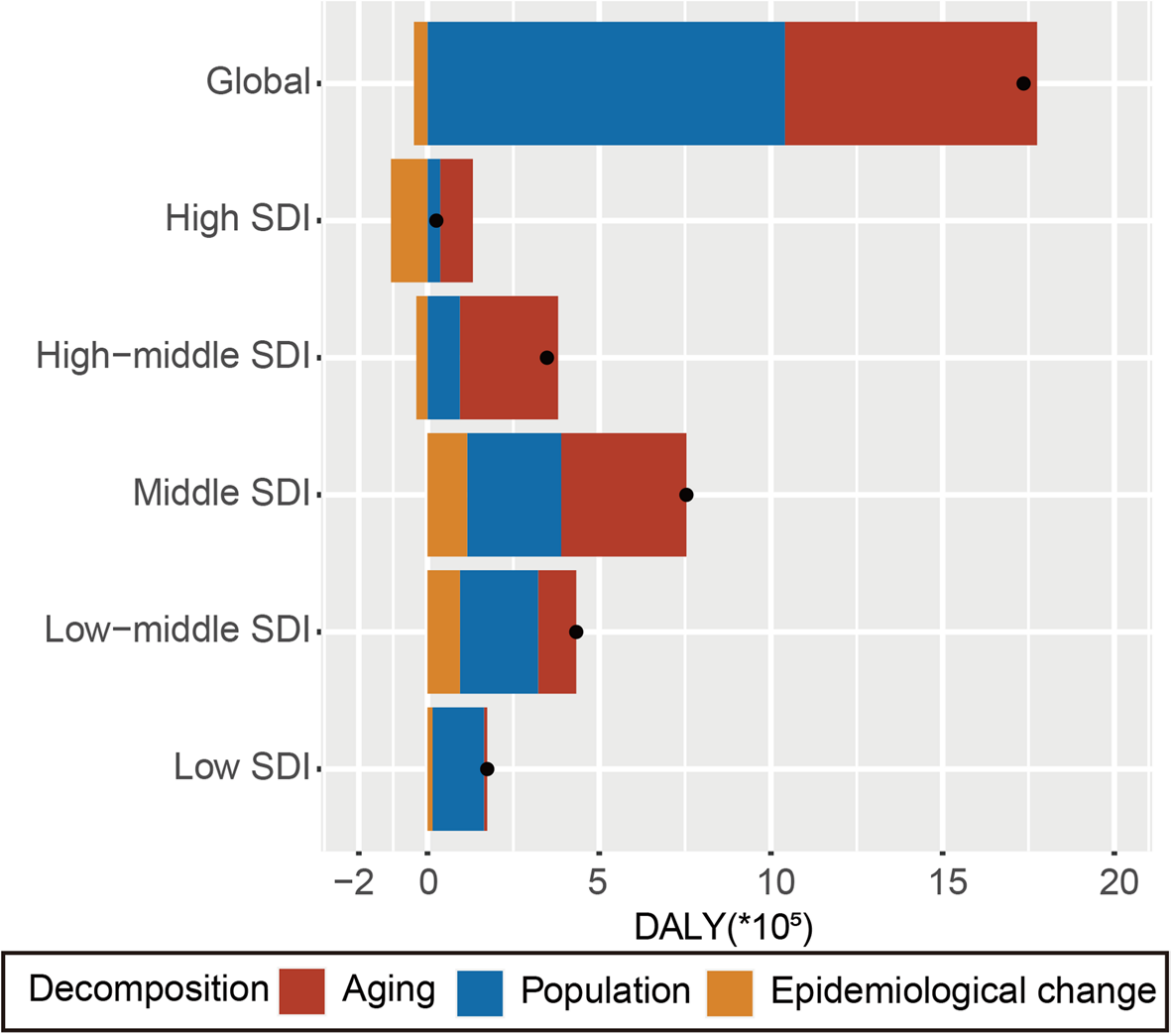
Changes in early-onset colorectal cancer DALYs according to population-level determinants of population growth, aging, and epidemiological change from 1990 to 2019 at the global level and by SDI quintile. The black dot represents the overall value of change contributed by all 3 components. For each component, the magnitude of a positive value indicates a corresponding increase in early-onset colorectal cancer DALYs attributed to the component; the magnitude of a negative value indicates a corresponding decrease in early-onset colorectal cancer DALYs attributed to the related component. SDI: Socio-demographic index; DALYs, Disability-Adjusted Life Years

The past 30 years have seen a significant global increase in DALYs, with the largest increase occurring in middle SDI quintile regions (Fig. 7). Aging and population growth accounted for 42.31% and 59.96%, of the worldwide increase in DALYs, respectively (Table S5), with the most significant aging contribution occurring in the high SDI quintile (370.5%), where population growth had the largest effect on DALYs growth (143.28%). The effect of epidemiological change on DALYs growth was negative (-2.27%) worldwide, and this effect was the most pronounced in the high SDI quintile (-413.78%). The effects of demography and epidemiology on DALYs differed across countries and regions (Table S5).

### Frontier analysis of age-standardized DALYs rates

**Fig. 8:**
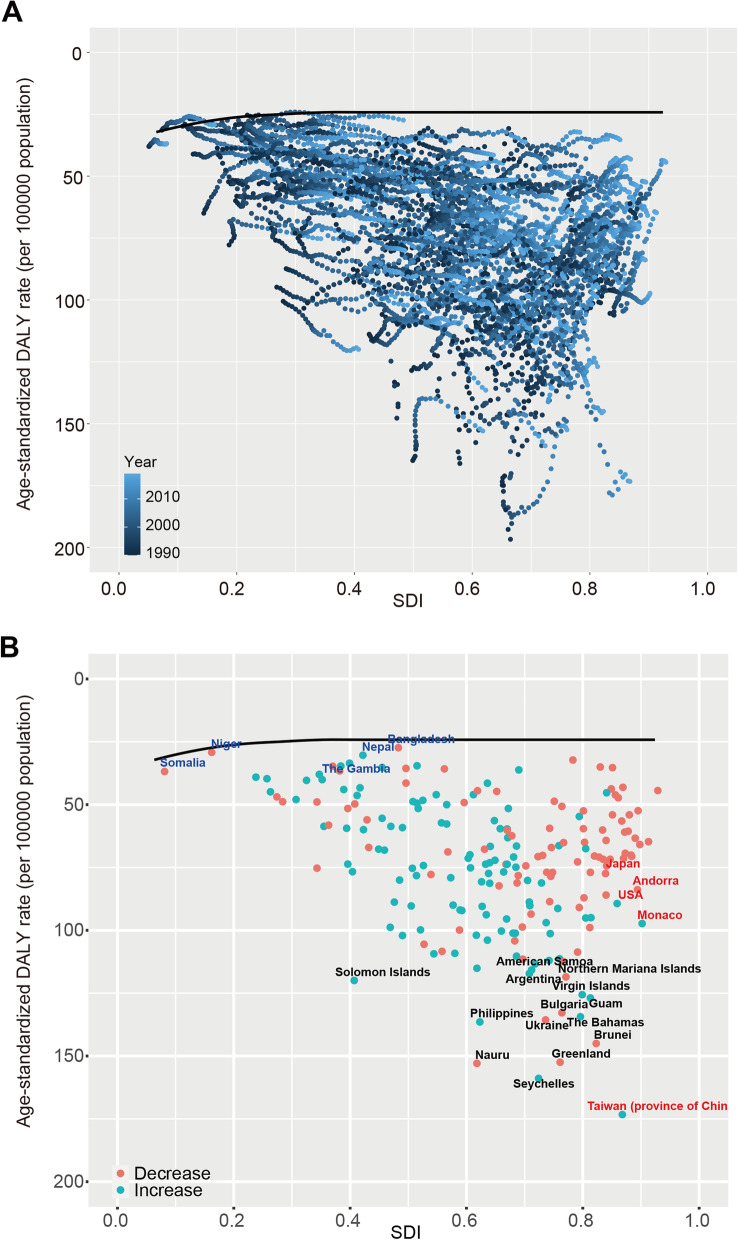
Frontier analysis based on SDI and age-standardized early-onset colorectal cancer DALY rate in 2019. The frontier is delineated in solid black color; countries and territories are represented as dots. The top 15 countries with the largest effective difference (largest early-onset colorectal cancer DALYs gap from the frontier) are labeled in black; examples of frontier countries with low SDI (< 0.5) and low effective difference are labeled in blue (e.g., Somalia, Niger, Nepal, Bangladesh, and the Gambia), and examples of countries and territories with high SDI (> 0.85) and relatively high effective difference for their level of development are labeled in red (e.g., USA, Japan, Andorra, Monaco, Taiwan (province of China). Red dots indicate an increase in age-standardized early-onset colorectal cancer DALYs rate from 1990 to 2019; blue dots indicate a decrease in age-standardized early-onset colorectal cancer DALYs rate between 1990 and 2019. SDI: Socio-demographic index; DALYs: Disability-Adjusted Life Years

The unrealized health gains of countries or regions at different levels of development during the period 1990–2019 are shown in Fig. 8A. Figure 8B and Table S6 show the DALYs burden and the effective difference in countries or regions with different sociodemographic development levels in 2019. With sociodemographic development, effective difference generally increased to some extent, indicating that countries or regions with a higher SDI have greater burden improvement potential (Fig. 8B).

### Risk factors of EO-CRC

In figure S5, we identified the contribution of 11 risk factors to DALYs due to EO-CRC for 21 GBD regions in 2019. Globally, a diet low in milk (32.8%), a diet low in whole grains (30.1%) played the main contributor to EO-CRC DALYs, while low physical activity (3.2%) and a diet low in fiber (4.5%) had less contribution to DALYs. Differences in regions’ development status could also affect the contribution of various risk factor to DALYs. In the high SDI region, a diet low in whole grains (31.2%) was the main risk factors. While in the low SDI region, the main risk factors was a diet low in calcium (39.8%). A diet low in calcium and milk were the main risk factor in Sub − Saharan Africa and Asia. In high-income North America, the main risk factor was a diet low in whole grains, while a diet low in milk was the main risk factor in the high-income Asia Pacific. (Figure S5).

## Discussion

Similar to previous studies, [3] we found 136.9% new EO-CRC incidence worldwide in 2019 compared to 30 years ago. The global EO-CRC incidence generally increased, but the cause was unclear, possibly due to the birth cohort effect [20]. As a result of economic and industrial development, developing world lifestyles have gradually westernized, increasing the probability of exposure to behavioral risk factors including high red or processed meat intake, obesity, lack of involvement in sports, sedentariness, and premature smoking and drinking among those born in the second half of the 20^th^ century [3, 5, 9, 21–24]. Studies have also shown that an increased EO-CRC risk in American women is associated with diet and lifestyle factors that lead to hyperinsulinemia [25]. Antibiotic use and changes in the gut microbiome may also play a role [26, 27].

The overall trend in age-standardized mortality rates worldwide was stable, and regions with a higher SDI quintile (high and high-middle SDI) had a decreasing mortality rate. Differences in global morbidity and mortality trends may be due to the normalization of screening tests (colonoscopy and fecal occult blood tests) and cancer registries. However, the corresponding increase in incidence due to the increased detection rate may be short-lived, as more adenomatous polyps will be excised during colonoscopy. Widespread use of early screening programs can significantly reduce mortality [28]. Improvements in medical technology, such as surgery, radiotherapy and chemotherapy, and targeted therapies, are also main reasons for the decline [29, 30].

In 2019, EO-CRC DALYs increased by 67.8% compared to 1990, with East Asia, Southeast Asia, and Southern Latin America bearing the heaviest burdens. Although developed regions have a higher EO-CRC incidence, we found that the increase in DALYs over the past 30 years occurred mainly in less developed regions, particularly middle- and low-SDI regions. We should pay attention to the frontier countries with low SDI in the frontier analysis, which showed excellent performance with limited resources. These countries’ practices and models could be valuable reference points for other countries with limited resources and a large burden. Conversely, some high SDI countries and regions such as Taiwan, Monaco, and the United States, performed poorly, suggesting that other factors have overwhelmed the health benefits of development. Future research is needed to further explore drivers in leading countries and barriers in lagging countries.

We found significantly more EO-CRC cases in men than in women, as well as age-standardized rates. Further, the gendered differences became more pronounced over time. According to the literature, men account for the majority of the colorectal cancer burden, mainly due to a higher prevalence of visceral fat, smoking, and drinking [31, 32]. In women, endogenous estrogens and oral contraceptives have been shown to reduce the cancer risk [33, 34]. We also found gendered differences in mortality, with EO-CRC-related mortality in males increasing and continuing to outpace that of females, while the female mortality rate declined, and women tended to have better survival outcomes [35]. Given the continued increase in the incidence of EO-CRC, recent recommendations to modify screening protocols are a good start. Additionally, the recommended age for screening could be lowered to 45 years, and the screening plan could be further modified for young adults with high-risk factors such as being male, smoking, obesity, a personal history of polyps or adenoma, and related family history [36].

The limitations of GBD research, as described in previous literature, are mainly in the following aspects. The first is that the inevitable loss of data severely affected the accuracy of the research. Inadequate cancer registries in some underdeveloped countries in Africa and Asia lead to underestimation of cases, as well as misdiagnosis or missed diagnosis due to poor health resources, although robust statistical methods were used to overcome this effect. Secondly, the data in this study are from different countries, which will inevitably lead to uneven quality, such as measurement bias, reporting delay, or disease misclassification. Finally, a GBD data lag occurred because the current estimates were calculated based on past trends and covariates [20, 37]. To balance, the limitation in GBD study, more international collaborations should be encouraged, including annual searching of available data with national collaborators. And data from more sources should be included in the statistics for cancer data, including epidemiological studies, vital registration systems, as well as cancer registries. In addition, the detailed cleaning, correction, and smoothing routines developed by GBD collaborators are also effective measures to overcome these limitations, including a series of advanced statistical modeling methods.

## Conclusion

This study provided a comprehensive estimate of the global EO-CRC burden. Age-standardized incidence and prevalence rates increased sharply worldwide between 1990 and 2019, while age-standardized death and DALYs rates changed less dramatically. The EO-CRC burden varied significantly with gender, sociodemographic development, and geographical location. We found that areas with a higher level of sociodemographic development tend to have a higher burden. This study can provide a basis for the formulation of relevant policies and the rational allocation of limited resources.

## Supplementary Information

**Additional file 1:** **Figure S1.** Age-standardized incidence rate at the global, regional, and national level. (A) Age-standardized incidence s rate of early-onset colorectal cancer globally and for 21 GBD regions by SDI, 1990–2019. (B) Age-standardized incidence rates of early-onset colorectal cancer for 204 countries and territories in 2019. The black line represents the expected age-standardized incidence rate of rheumatic heart disease based solely on SDI. GBD: Global Burden of Diseases, Injuries, and Risk Factors Study; SDI: Socio-demographic index; DALYs, Disability-Adjusted Life Years.

**Additional file 2:** **Figure S2.** Age-standardized prevalence rate at the global, regional, and national level. (A) Age-standardized prevalence rate of early-onset colorectal cancer globally and for 21 GBD regions by SDI, 1990–2019. (B) Age-standardized prevalence rates of early-onset colorectal cancer for 204 countries and territories in 2019. The black line represents the expected age-standardized prevalence rate of rheumatic heart disease based solely on SDI. GBD: Global Burden of Diseases, Injuries, and Risk Factors Study; SDI: Socio-demographic index; DALYs, Disability-Adjusted Life Years.

**Additional file 3:** **Figure S3.** Age-standardized mortality rate at the global, regional, and national level. (A) Age-standardized mortality rate of early-onset colorectal cancer globally and for 21 GBD regions by SDI, 1990–2019. (B) Age-standardized mortality rates of early-onset colorectal cancer for 204 countries and territories in 2019. The black line represents the expected age-standardized mortality rate of rheumatic heart disease based solely on SDI. GBD: Global Burden of Diseases, Injuries, and Risk Factors Study; SDI: Socio-demographic index; DALYs, Disability-Adjusted Life Years.

**Additional file 4:** **Figure S4.** Age-standardized DALYs rate at the global, regional, and national level. (A) Age-standardized DALYs rate of early-onset colorectal cancer globally and for 21 GBD regions by SDI, 1990–2019. (B) Age-standardized DALYs rates of early-onset colorectal cancer for 204 countries and territories in 2019. The black line represents the expected age-standardized DALYs rate of rheumatic heart disease based solely on SDI. GBD: Global Burden of Diseases, Injuries, and Risk Factors Study; SDI: Socio-demographic index; DALYs, Disability-Adjusted Life Years.

**Additional file 5:** **Figure S5**. Percentage of age-standardised DALYs due to early-onset colorectal cancer attributable to risk factors for 21 GBD regions, both sexes, 2019. GBD: Global Burden of Diseases, Injuries, and Risk Factors Study; SDI: Socio-demographic index; DALYs, Disability-Adjusted Life Years.

**Additional file 6:** **Table S1.** Incidence of early-onset colorectal cancer in 1990 and 2019 with AAPC from 2009 and 2019 at countries/territories level, both sexes.

**Additional file 7:** **Table S2.** Prevalence of early-onset colorectal cancer in 1990 and 2019 with AAPC from 2009 and 2019 at countries/territories level, both sexes.

**Additional file 8:** **Table S3.** Mortality of early-onset colorectal cancer in 1990 and 2019 with AAPC from 2009 and 2019 at countries/territories level, both sexes.

**Additional file 9:** **Table S4.** DALY of early-onset colorectal cancer in 1990 and 2019 with AAPC from 2009 and 2019 at countries/territories level, both sexes.

**Additional file 10:** **Table S5.** Changes in DALYs number according to population-level determinants and causes from 1990 to 2019.

**Additional file 11:** **Table S6.** Frontier DALYs, and effective difference by country or territory.

## Data Availability

Publicly available datasets were analyzed in this study. The data can be found here: http://ghdx.healthdata.org/gbd-results-tool.
