# Supplementary figures and images for: The global, regional, and national early-onset colorectal cancer burden and trends from 1990 to 2019: results from the Global Burden of Disease Study 2019
Source: BMC Public Health. 2022 Oct 12;22:1896. doi: 10.1186/s12889-022-14274-7 (PMC9555189; doi:10.1186/s12889-022-14274-7)

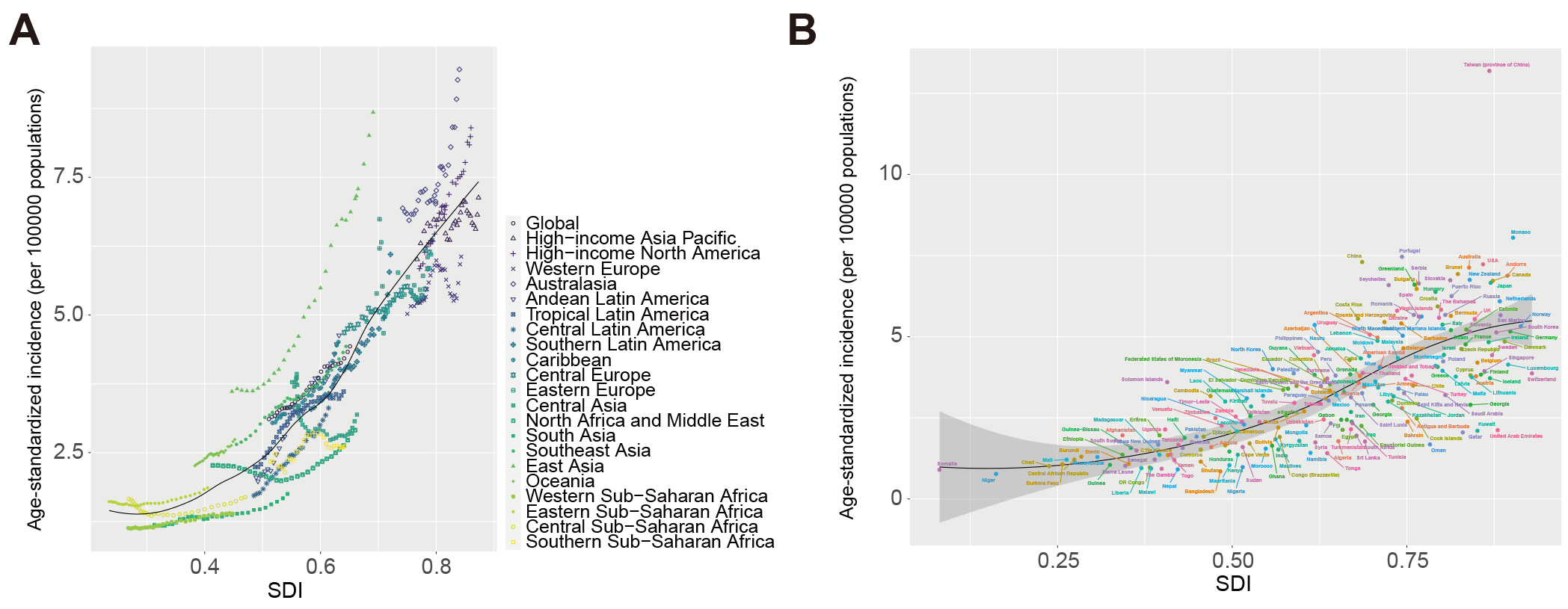

Supplement: Supplementary file 1 — Additional file 1: Figure S1. Age-standardized incidence rate at the global, regional, and national level. (A) Age-standardized incidence s rate of early-onset colorectal cancer globally and for 21 GBD regions by SDI, 1990–2019. (B) Age-standardized incidence rates of early-onset colorectal cancer for 204 countries and territories in 2019. The black line represents the expected age-standardized incidence rate of rheumatic heart disease based solely on SDI. GBD: Global Burden of Diseases, Injuries, and Risk Factors Study; SDI: Socio-demographic index; DALYs, Disability-Adjusted Life Years. [file 12889_2022_14274_MOESM1_ESM.tif]

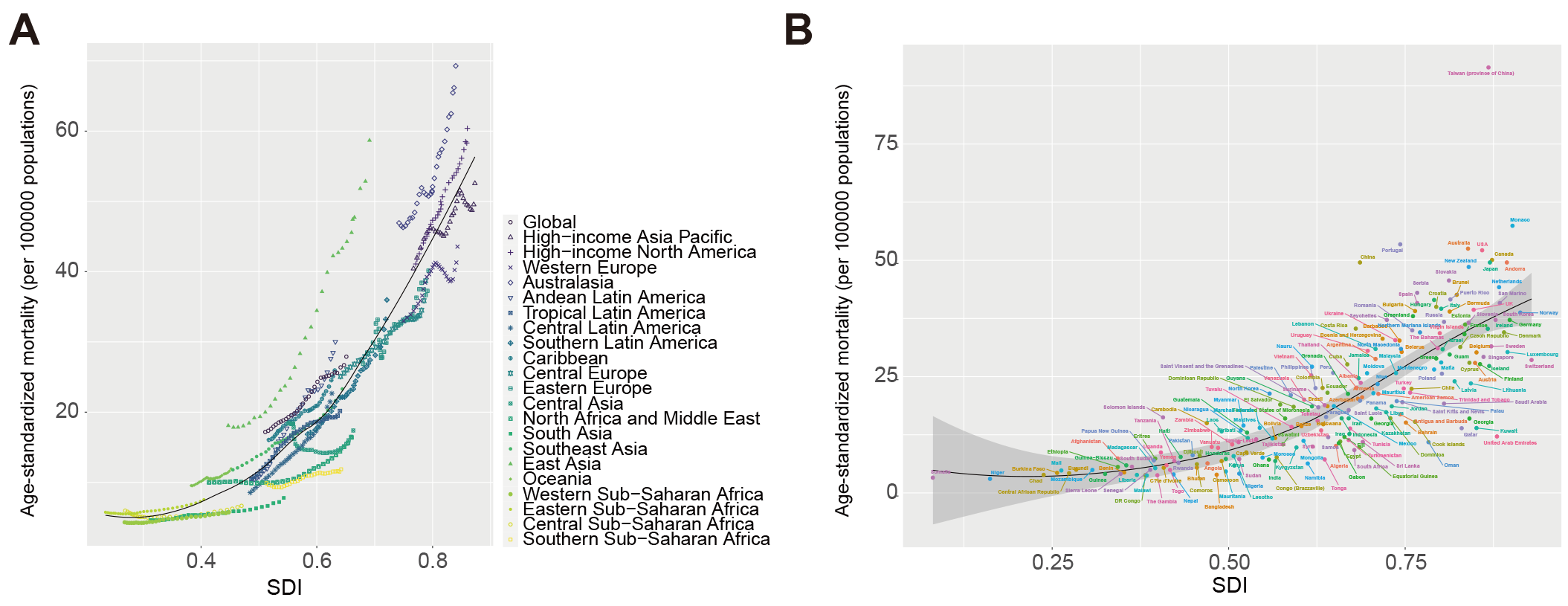

Supplement: Supplementary file 2 — Additional file 2: Figure S2. Age-standardized prevalence rate at the global, regional, and national level. (A) Age-standardized prevalence rate of early-onset colorectal cancer globally and for 21 GBD regions by SDI, 1990–2019. (B) Age-standardized prevalence rates of early-onset colorectal cancer for 204 countries and territories in 2019. The black line represents the expected age-standardized prevalence rate of rheumatic heart disease based solely on SDI. GBD: Global Burden of Diseases, Injuries, and Risk Factors Study; SDI: Socio-demographic index; DALYs, Disability-Adjusted Life Years. [file 12889_2022_14274_MOESM2_ESM.tif]

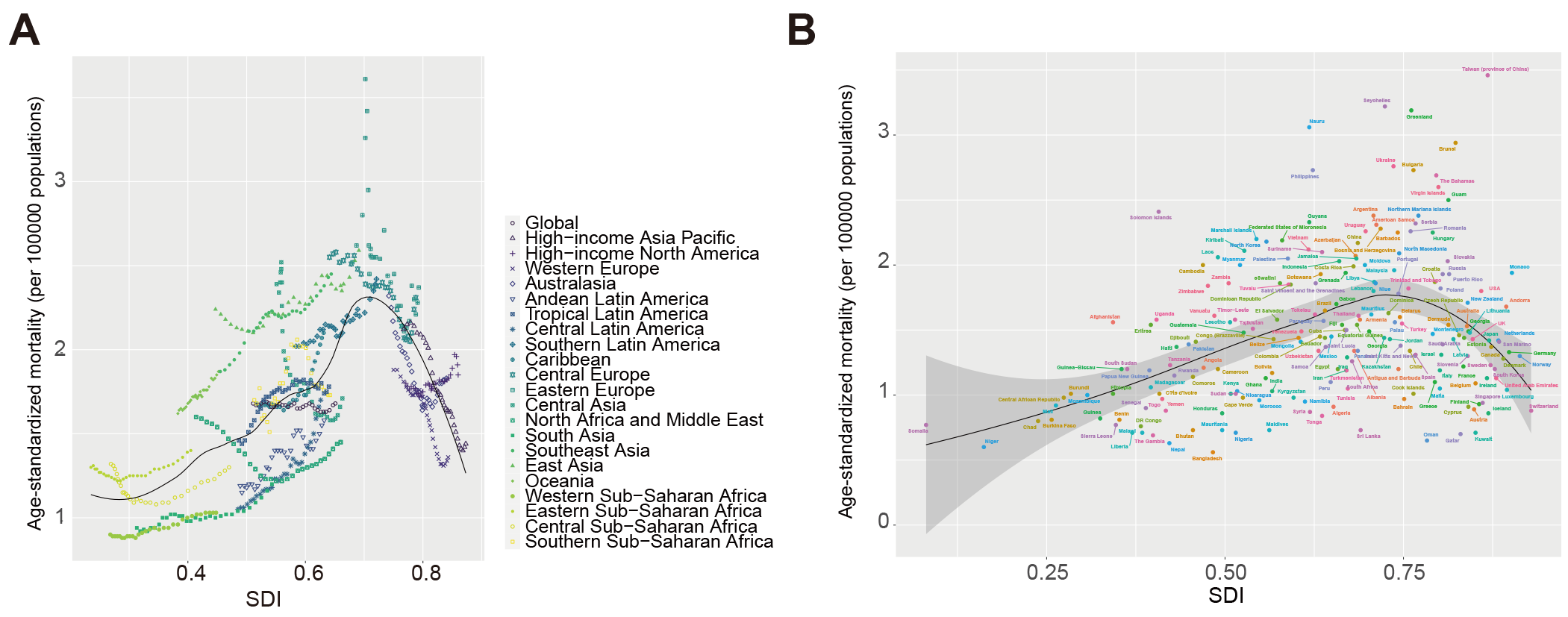

Supplement: Supplementary file 3 — Additional file 3: Figure S3. Age-standardized mortality rate at the global, regional, and national level. (A) Age-standardized mortality rate of early-onset colorectal cancer globally and for 21 GBD regions by SDI, 1990–2019. (B) Age-standardized mortality rates of early-onset colorectal cancer for 204 countries and territories in 2019. The black line represents the expected age-standardized mortality rate of rheumatic heart disease based solely on SDI. GBD: Global Burden of Diseases, Injuries, and Risk Factors Study; SDI: Socio-demographic index; DALYs, Disability-Adjusted Life Years. [file 12889_2022_14274_MOESM3_ESM.tif]

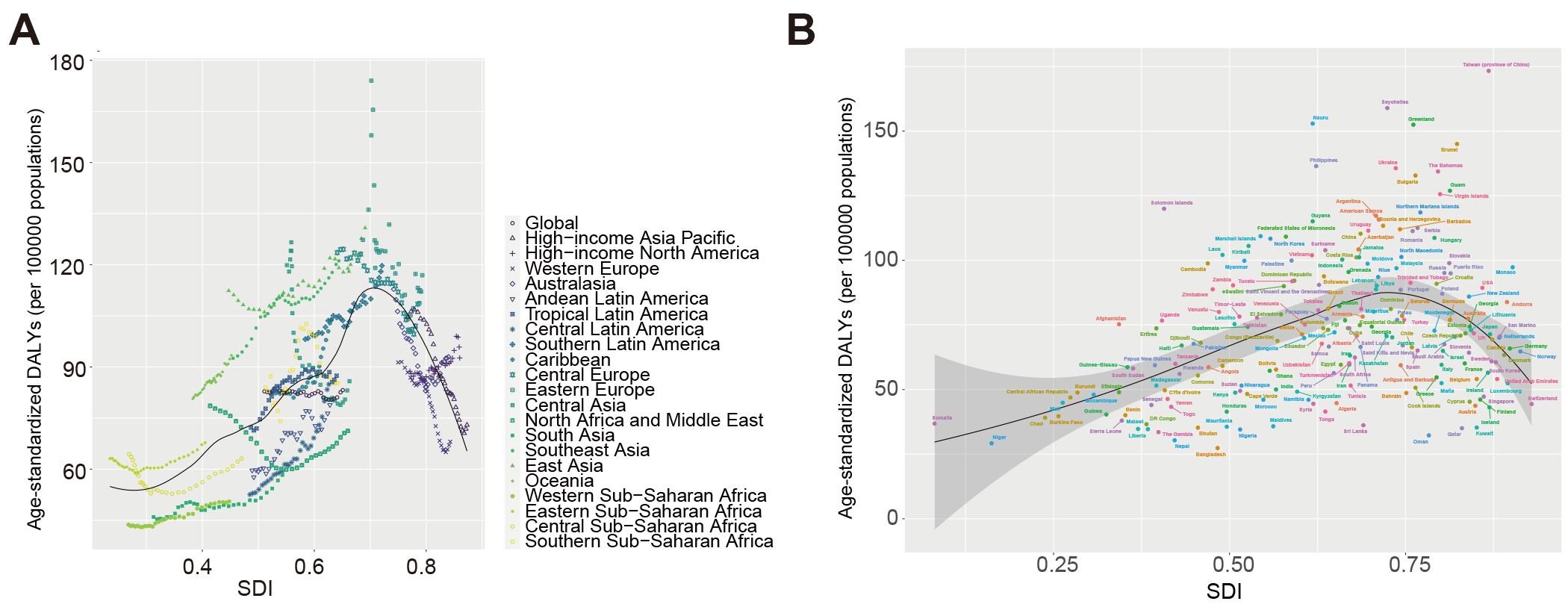

Supplement: Supplementary file 4 — Additional file 4: Figure S4. Age-standardized DALYs rate at the global, regional, and national level. (A) Age-standardized DALYs rate of early-onset colorectal cancer globally and for 21 GBD regions by SDI, 1990–2019. (B) Age-standardized DALYs rates of early-onset colorectal cancer for 204 countries and territories in 2019. The black line represents the expected age-standardized DALYs rate of rheumatic heart disease based solely on SDI. GBD: Global Burden of Diseases, Injuries, and Risk Factors Study; SDI: Socio-demographic index; DALYs, Disability-Adjusted Life Years. [file 12889_2022_14274_MOESM4_ESM.tif]

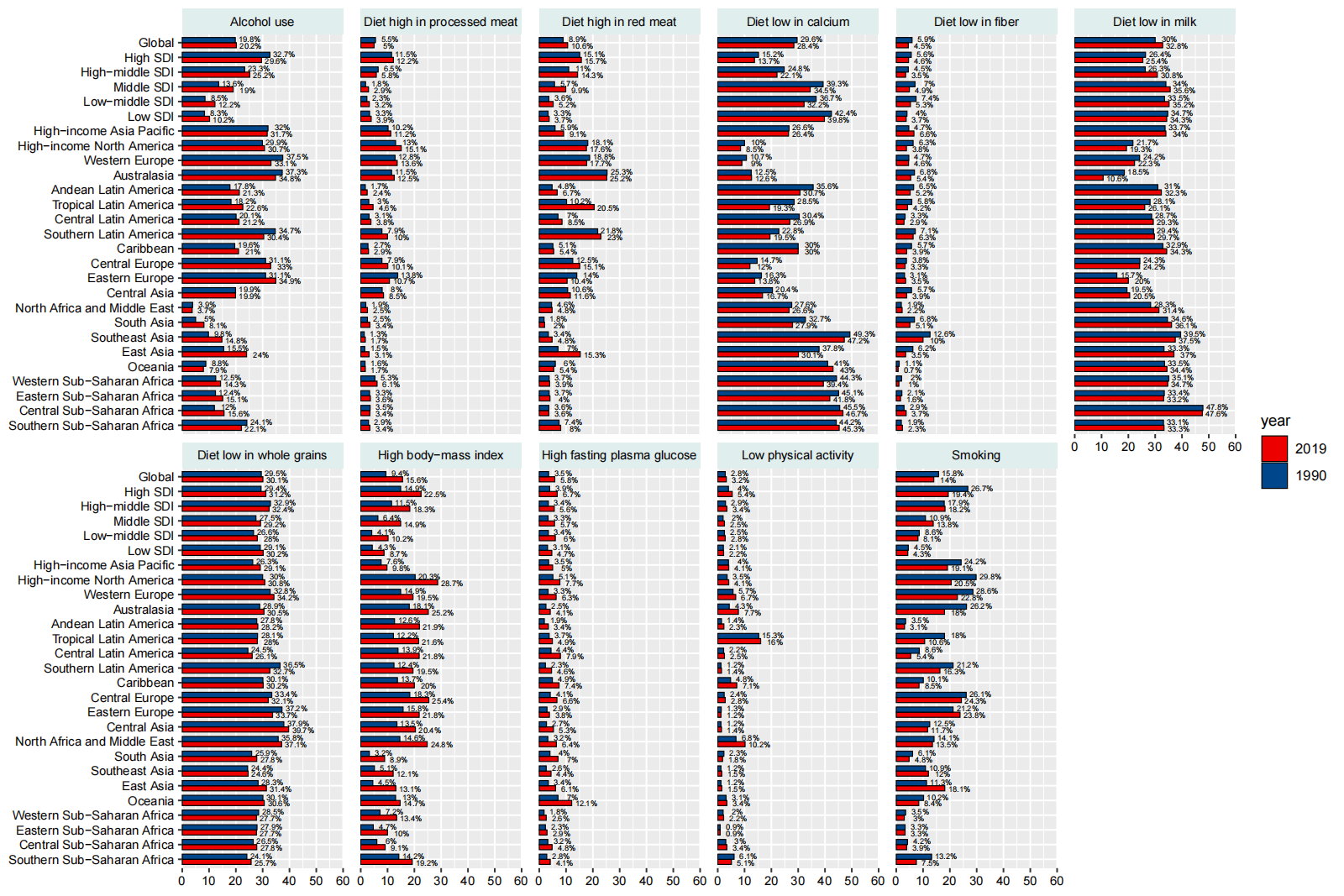

Supplement: Supplementary file 5 — Additional file 5: Figure S5. Percentage of age-standardised DALYs due to early-onset colorectal cancer attributable to risk factors for 21 GBD regions, both sexes, 2019. GBD: Global Burden of Diseases, Injuries, and Risk Factors Study; SDI: Socio-demographic index; DALYs, Disability-Adjusted Life Years. [file 12889_2022_14274_MOESM5_ESM.tif]
