# Supplementary material for: The global, regional, and national early-onset colorectal cancer burden and trends from 1990 to 2019: results from the Global Burden of Disease Study 2019
Source: BMC Public Health. 2022 Oct 12;22:1896. doi: 10.1186/s12889-022-14274-7 (PMC9555189; doi:10.1186/s12889-022-14274-7)
Supplement: Supplementary file 6 — Additional file 6: Table S1. Incidence of early-onset colorectal cancer in 1990 and 2019 with AAPC from 2009 and 2019 at countries/territories level, both sexes. [file 12889_2022_14274_MOESM6_ESM.docx]

Table S1. Incidence of early-onset colorectal cancer in 1990 and 2019 with AAPC from 2009 and 2019 at countries/territories level, both sexes.

| Countries/territories | 1990 | |  | 2019 | | AAPC % (95% CI)  1990-2019 |
| --- | --- | --- | --- | --- | --- | --- |
|  | Cases (95% UI) | Age-standardized incidence per  100 000 population (95% UI) |  | Cases (95% UI) | Age-standardized incidence per  100 000 population (95% UI) |  |
| Afghanistan | 101 (40 to 186) | 1.92 (0.75 to 3.56) |  | 388 (197 to 635) | 1.96 (0.99 to 3.22) | 0.1 (0.03 to 0.16) |
| Albania | 44 (35 to 56) | 2.16 (1.71 to 2.68) |  | 65 (43 to 93) | 3.57 (2.36 to 5.16) | 1.62 (1.25 to 2) |
| Algeria | 183 (127 to 257) | 1.55 (1.09 to 2.16) |  | 565 (375 to 818) | 1.78 (1.18 to 2.58) | 0.56 (0.39 to 0.72) |
| American Samoa | 1 (1 to 1) | 3.33 (2.25 to 4.74) |  | 1 (1 to 2) | 4.08 (2.65 to 6.11) | 0.93 (0.37 to 1.5) |
| Andorra | 3 (2 to 4) | 6.13 (3.77 to 9.3) |  | 6 (4 to 8) | 6.88 (4.31 to 10.22) | 0.33 (0.18 to 0.48) |
| Angola | 67 (39 to 109) | 1.32 (0.77 to 2.15) |  | 238 (143 to 358) | 1.6 (0.97 to 2.4) | 0.68 (0.47 to 0.9) |
| Antigua and Barbuda | 1 (1 to 1) | 2.25 (1.76 to 2.86) |  | 2 (1 to 3) | 2.8 (2.04 to 3.79) | 0.69 (0.45 to 0.93) |
| Argentina | 695 (610 to 793) | 3.24 (2.84 to 3.69) |  | 1640 (1162 to 2246) | 4.97 (3.52 to 6.81) | 1.37 (1.19 to 1.55) |
| Armenia | 80 (67 to 95) | 4.06 (3.39 to 4.76) |  | 75 (57 to 96) | 3.46 (2.63 to 4.44) | -0.61 (-1.72 to 0.5) |
| Australia | 770 (686 to 862) | 6.11 (5.44 to 6.83) |  | 1310 (927 to 1814) | 7.13 (5.03 to 9.86) | 0.8 (0.44 to 1.15) |
| Austria | 412 (351 to 476) | 6.98 (5.95 to 8.08) |  | 262 (187 to 356) | 3.78 (2.69 to 5.14) | -2.32 (-2.72 to -1.92) |
| Azerbaijan | 176 (143 to 217) | 4.38 (3.58 to 5.35) |  | 298 (207 to 423) | 3.79 (2.63 to 5.39) | -0.26 (-0.92 to 0.4) |
| Bahamas | 7 (6 to 9) | 4.35 (3.52 to 5.32) |  | 17 (12 to 23) | 5.58 (4.1 to 7.55) | 0.83 (0.11 to 1.55) |
| Bahrain | 6 (4 to 8) | 1.78 (1.24 to 2.44) |  | 39 (25 to 57) | 2.38 (1.57 to 3.5) | 1.15 (0.82 to 1.49) |
| Bangladesh | 421 (260 to 630) | 0.79 (0.5 to 1.18) |  | 940 (552 to 1520) | 0.85 (0.5 to 1.37) | 0.29 (0.02 to 0.56) |
| Barbados | 7 (5 to 8) | 3.97 (3.28 to 4.74) |  | 12 (9 to 16) | 5.41 (3.99 to 7.22) | 1.16 (0.72 to 1.6) |
| Belarus | 349 (301 to 403) | 5.17 (4.46 to 5.97) |  | 342 (230 to 483) | 4.64 (3.12 to 6.56) | -0.47 (-1.23 to 0.29) |
| Belgium | 327 (278 to 383) | 4.63 (3.94 to 5.43) |  | 354 (246 to 495) | 4.2 (2.91 to 5.87) | -0.48 (-0.83 to -0.14) |
| Belize | 1 (1 to 1) | 1.09 (0.87 to 1.34) |  | 8 (6 to 10) | 2.7 (2.07 to 3.44) | 2.87 (1.72 to 4.04) |
| Benin | 20 (13 to 28) | 0.91 (0.62 to 1.29) |  | 68 (42 to 105) | 1.08 (0.67 to 1.65) | 0.6 (0.49 to 0.72) |
| Bermuda | 2 (2 to 3) | 4.72 (3.65 to 6.01) |  | 3 (2 to 4) | 5.64 (3.93 to 7.82) | 0.52 (0.06 to 0.98) |
| Bhutan | 2 (1 to 4) | 0.84 (0.41 to 1.42) |  | 6 (3 to 10) | 1.15 (0.53 to 1.93) | 1.08 (1 to 1.16) |
| Bolivia (Plurinational State of) | 51 (33 to 72) | 1.49 (0.97 to 2.1) |  | 170 (98 to 264) | 2.2 (1.27 to 3.41) | 1.4 (1.22 to 1.58) |
| Bosnia and Herzegovina | 100 (82 to 122) | 3.21 (2.64 to 3.89) |  | 137 (97 to 187) | 5.45 (3.85 to 7.45) | 1.65 (1.25 to 2.05) |
| Botswana | 10 (6 to 17) | 1.72 (1.02 to 2.72) |  | 52 (29 to 85) | 3.09 (1.71 to 5.06) | 1.98 (1.7 to 2.26) |
| Brazil | 1918 (1802 to 2042) | 2.13 (2 to 2.27) |  | 5639 (5187 to 6103) | 3.34 (3.08 to 3.62) | 1.64 (1.49 to 1.79) |
| Brunei Darussalam | 9 (6 to 12) | 5.46 (3.83 to 7.5) |  | 26 (19 to 36) | 6.93 (4.96 to 9.49) | 0.92 (0.34 to 1.51) |
| Bulgaria | 355 (306 to 410) | 5.5 (4.73 to 6.36) |  | 364 (254 to 504) | 6.47 (4.51 to 8.96) | 0.7 (0.19 to 1.22) |
| Burkina Faso | 34 (22 to 50) | 0.8 (0.52 to 1.18) |  | 121 (76 to 180) | 1.07 (0.68 to 1.59) | 1.19 (0.8 to 1.57) |
| Burundi | 38 (24 to 57) | 1.53 (0.96 to 2.3) |  | 75 (46 to 118) | 1.3 (0.79 to 2.05) | -0.65 (-0.88 to -0.42) |
| Cabo Verde | 1 (1 to 2) | 1.05 (0.75 to 1.43) |  | 7 (4 to 10) | 1.71 (1.11 to 2.57) | 1.64 (1.46 to 1.82) |
| Cambodia | 98 (60 to 145) | 1.98 (1.23 to 2.93) |  | 343 (226 to 508) | 3.17 (2.09 to 4.68) | 1.57 (1.31 to 1.83) |
| Cameroon | 66 (45 to 92) | 1.36 (0.93 to 1.89) |  | 257 (152 to 407) | 1.65 (0.98 to 2.61) | 0.64 (0.58 to 0.71) |
| Canada | 1171 (1025 to 1335) | 5.57 (4.88 to 6.36) |  | 1767 (1226 to 2470) | 6.72 (4.66 to 9.4) | 0.75 (0.51 to 0.99) |
| Central African Republic | 18 (11 to 27) | 1.35 (0.82 to 2.02) |  | 34 (20 to 55) | 1.21 (0.7 to 1.93) | -0.46 (-0.79 to -0.12) |
| Chad | 20 (13 to 29) | 0.75 (0.5 to 1.08) |  | 72 (46 to 108) | 1.01 (0.64 to 1.51) | 1.1 (0.97 to 1.23) |
| Chile | 173 (145 to 204) | 2 (1.68 to 2.35) |  | 479 (332 to 662) | 3.49 (2.42 to 4.82) | 2.43 (1.72 to 3.14) |
| China | 24194 (20726 to 28159) | 3.05 (2.62 to 3.55) |  | 87551 (72074 to 104615) | 7.3 (6.02 to 8.71) | 3 (2.75 to 3.24) |
| Colombia | 385 (332 to 444) | 1.97 (1.71 to 2.26) |  | 1248 (853 to 1770) | 3.66 (2.5 to 5.19) | 2.25 (2.1 to 2.4) |
| Comoros | 3 (1 to 5) | 1.24 (0.34 to 2.12) |  | 7 (4 to 11) | 1.52 (0.81 to 2.37) | 0.26 (-1.53 to 2.08) |
| Congo | 24 (13 to 39) | 2.18 (1.16 to 3.55) |  | 65 (38 to 103) | 1.91 (1.1 to 3.01) | -0.28 (-0.67 to 0.11) |
| Cook Islands | 0 (0 to 0) | 2.1 (1.34 to 3.17) |  | 0 (0 to 0) | 2.48 (1.32 to 3.8) | 0.61 (0.47 to 0.76) |
| Costa Rica | 42 (35 to 51) | 2.44 (2.01 to 2.93) |  | 192 (135 to 269) | 5.55 (3.9 to 7.77) | 3.12 (2.89 to 3.35) |
| Croatia | 181 (151 to 215) | 4.98 (4.15 to 5.92) |  | 189 (130 to 267) | 5.93 (4.08 to 8.36) | 0.55 (0.26 to 0.83) |
| Cuba | 262 (223 to 307) | 3.45 (2.95 to 4.05) |  | 394 (287 to 531) | 4.28 (3.11 to 5.8) | 0.75 (0.54 to 0.95) |
| Cyprus | 11 (8 to 15) | 1.95 (1.39 to 2.62) |  | 42 (30 to 56) | 3.81 (2.71 to 5.15) | 2.21 (1.71 to 2.71) |
| Czechia | 511 (453 to 579) | 6.2 (5.48 to 7.04) |  | 429 (310 to 579) | 4.62 (3.32 to 6.23) | -1.11 (-1.36 to -0.86) |
| C么te d'Ivoire | 77 (50 to 114) | 1.33 (0.87 to 1.95) |  | 204 (125 to 308) | 1.35 (0.83 to 2.03) | 0.03 (-0.13 to 0.18) |
| Democratic People's Republic of Korea | 568 (343 to 874) | 4.02 (2.42 to 6.17) |  | 864 (491 to 1422) | 4 (2.26 to 6.6) | -0.13 (-0.3 to 0.03) |
| Democratic Republic of the Congo | 177 (112 to 267) | 1.02 (0.66 to 1.53) |  | 432 (252 to 697) | 0.97 (0.57 to 1.56) | -0.22 (-0.37 to -0.07) |
| Denmark | 188 (163 to 216) | 4.49 (3.88 to 5.17) |  | 203 (142 to 280) | 4.85 (3.4 to 6.71) | 0 (-0.44 to 0.43) |
| Djibouti | 3 (2 to 5) | 1.42 (0.83 to 2.22) |  | 16 (9 to 27) | 1.92 (1.05 to 3.18) | 1.06 (0.85 to 1.26) |
| Dominica | 1 (1 to 1) | 2.4 (1.74 to 3.18) |  | 1 (1 to 2) | 3.03 (2.03 to 4.34) | 0.78 (0.57 to 0.99) |
| Dominican Republic | 63 (47 to 83) | 1.6 (1.2 to 2.09) |  | 265 (166 to 397) | 3.48 (2.18 to 5.21) | 3.04 (2.89 to 3.19) |
| Ecuador | 80 (65 to 97) | 1.42 (1.17 to 1.72) |  | 422 (288 to 595) | 3.53 (2.41 to 4.96) | 3.02 (2.6 to 3.43) |
| Egypt | 462 (348 to 607) | 1.44 (1.1 to 1.87) |  | 1383 (849 to 2099) | 2.13 (1.3 to 3.23) | 1.35 (0.93 to 1.77) |
| El Salvador | 36 (29 to 44) | 1.3 (1.05 to 1.6) |  | 140 (93 to 204) | 3.36 (2.22 to 4.88) | 3.44 (3.04 to 3.84) |
| Equatorial Guinea | 2 (1 to 4) | 1.12 (0.65 to 1.79) |  | 16 (8 to 28) | 2.25 (1.13 to 3.98) | 2.51 (2.26 to 2.77) |
| Eritrea | 18 (11 to 27) | 1.31 (0.79 to 1.95) |  | 72 (44 to 112) | 1.96 (1.2 to 3.01) | 1.39 (1.18 to 1.6) |
| Estonia | 50 (41 to 59) | 4.47 (3.69 to 5.36) |  | 52 (35 to 74) | 5.22 (3.52 to 7.43) | 0.68 (-0.41 to 1.77) |
| Eswatini | 5 (3 to 8) | 1.47 (0.95 to 2.2) |  | 17 (9 to 29) | 2.57 (1.36 to 4.35) | 1.9 (1.68 to 2.13) |
| Ethiopia | 376 (226 to 573) | 1.63 (0.99 to 2.48) |  | 720 (504 to 1029) | 1.36 (0.95 to 1.94) | -0.71 (-0.88 to -0.54) |
| Fiji | 9 (6 to 13) | 2.01 (1.38 to 2.84) |  | 16 (10 to 23) | 2.44 (1.61 to 3.55) | 0.69 (0.4 to 0.98) |
| Finland | 151 (128 to 178) | 3.65 (3.08 to 4.29) |  | 142 (97 to 198) | 3.83 (2.61 to 5.35) | -0.07 (-0.45 to 0.32) |
| France | 1728 (1521 to 1954) | 4.24 (3.73 to 4.79) |  | 2268 (1564 to 3182) | 4.76 (3.29 to 6.67) | 0.31 (0.12 to 0.51) |
| Gabon | 11 (6 to 19) | 2.46 (1.32 to 4.26) |  | 27 (16 to 43) | 2.44 (1.41 to 3.9) | 0.01 (-0.27 to 0.29) |
| Gambia | 3 (2 to 5) | 0.67 (0.41 to 1.01) |  | 11 (6 to 17) | 0.93 (0.55 to 1.47) | 1.33 (0.57 to 2.1) |
| Georgia | 157 (126 to 193) | 3.77 (3.03 to 4.63) |  | 113 (81 to 153) | 2.9 (2.09 to 3.93) | -1.51 (-2.28 to -0.74) |
| Germany | 2959 (2609 to 3337) | 4.96 (4.36 to 5.6) |  | 3045 (2129 to 4278) | 5.16 (3.61 to 7.25) | -0.54 (-1.15 to 0.06) |
| Ghana | 88 (58 to 126) | 1.14 (0.76 to 1.63) |  | 327 (202 to 494) | 1.64 (1.02 to 2.47) | 1.23 (1.07 to 1.4) |
| Greece | 213 (181 to 249) | 2.9 (2.47 to 3.41) |  | 340 (245 to 461) | 4.07 (2.93 to 5.52) | 0.98 (0.53 to 1.43) |
| Greenland | 3 (2 to 3) | 5.8 (4.1 to 7.93) |  | 2 (2 to 4) | 6.6 (4.55 to 9.53) | 0.33 (0.12 to 0.53) |
| Grenada | 1 (1 to 2) | 2.86 (2.18 to 3.71) |  | 3 (2 to 4) | 3.84 (2.78 to 5.13) | 0.89 (0.66 to 1.12) |
| Guam | 3 (2 to 5) | 3.68 (2.53 to 5.25) |  | 6 (4 to 9) | 5.2 (3.52 to 7.43) | 1.32 (0.82 to 1.82) |
| Guatemala | 42 (34 to 53) | 1.07 (0.86 to 1.33) |  | 283 (204 to 380) | 2.55 (1.84 to 3.42) | 3.12 (2.67 to 3.58) |
| Guinea | 24 (17 to 33) | 0.8 (0.55 to 1.1) |  | 65 (41 to 98) | 1.05 (0.67 to 1.58) | 1.03 (0.9 to 1.15) |
| Guinea-Bissau | 7 (4 to 10) | 1.5 (0.91 to 2.29) |  | 15 (10 to 23) | 1.57 (0.98 to 2.33) | 0.14 (0.06 to 0.21) |
| Guyana | 10 (7 to 13) | 2.35 (1.76 to 3.06) |  | 20 (14 to 30) | 3.82 (2.55 to 5.56) | 1.61 (0.61 to 2.63) |
| Haiti | 64 (36 to 96) | 1.9 (1.1 to 2.86) |  | 148 (86 to 232) | 1.88 (1.09 to 2.94) | -0.03 (-0.21 to 0.14) |
| Honduras | 28 (20 to 39) | 1.26 (0.89 to 1.72) |  | 86 (47 to 146) | 1.46 (0.81 to 2.46) | 0.55 (0.28 to 0.81) |
| Hungary | 476 (412 to 548) | 5.97 (5.15 to 6.88) |  | 527 (384 to 703) | 6.38 (4.64 to 8.52) | 0.05 (-0.76 to 0.86) |
| Iceland | 6 (5 to 8) | 3.61 (2.86 to 4.49) |  | 9 (7 to 12) | 3.72 (2.77 to 4.87) | 0.01 (-0.24 to 0.27) |
| India | 5262 (4469 to 6132) | 1.05 (0.89 to 1.22) |  | 14907 (12165 to 17810) | 1.52 (1.24 to 1.81) | 1.31 (1.04 to 1.59) |
| Indonesia | 2253 (1605 to 2842) | 2.06 (1.48 to 2.6) |  | 6812 (4774 to 9014) | 3.29 (2.31 to 4.35) | 1.59 (1.51 to 1.66) |
| Iran (Islamic Republic of) | 460 (368 to 576) | 1.67 (1.34 to 2.09) |  | 1884 (1659 to 2145) | 2.68 (2.36 to 3.05) | 1.61 (1.45 to 1.78) |
| Iraq | 133 (81 to 209) | 1.61 (0.99 to 2.52) |  | 658 (427 to 995) | 2.38 (1.55 to 3.6) | 1.57 (1.16 to 1.98) |
| Ireland | 119 (99 to 140) | 5.03 (4.18 to 5.94) |  | 193 (131 to 272) | 4.83 (3.29 to 6.84) | -0.34 (-0.75 to 0.07) |
| Israel | 113 (94 to 136) | 3.69 (3.07 to 4.43) |  | 284 (197 to 395) | 4.44 (3.07 to 6.17) | 0.54 (0.12 to 0.96) |
| Italy | 2265 (2056 to 2490) | 5.44 (4.94 to 5.99) |  | 2652 (2081 to 3299) | 5.37 (4.22 to 6.67) | -0.23 (-0.36 to -0.09) |
| Jamaica | 19 (15 to 24) | 1.54 (1.21 to 1.94) |  | 88 (60 to 124) | 4.33 (2.94 to 6.14) | 4.39 (2.16 to 6.67) |
| Japan | 6835 (6477 to 7213) | 6.36 (6.03 to 6.72) |  | 6662 (5440 to 8009) | 6.66 (5.45 to 7.99) | 0.26 (-0.03 to 0.54) |
| Jordan | 41 (29 to 57) | 2.51 (1.76 to 3.46) |  | 253 (180 to 348) | 3.16 (2.25 to 4.33) | 0.89 (0.66 to 1.12) |
| Kazakhstan | 369 (320 to 421) | 3.78 (3.29 to 4.3) |  | 409 (321 to 517) | 3 (2.35 to 3.79) | -0.81 (-1.62 to 0.01) |
| Kenya | 82 (55 to 105) | 0.85 (0.57 to 1.08) |  | 380 (286 to 493) | 1.36 (1.03 to 1.76) | 1.52 (1.33 to 1.71) |
| Kiribati | 1 (1 to 2) | 2.96 (2 to 4.31) |  | 2 (1 to 3) | 2.85 (1.79 to 4.45) | -0.16 (-0.23 to -0.09) |
| Kuwait | 18 (14 to 23) | 1.51 (1.15 to 1.91) |  | 98 (69 to 135) | 2.1 (1.47 to 2.9) | 1.55 (0.84 to 2.25) |
| Kyrgyzstan | 77 (62 to 93) | 3.34 (2.71 to 4.06) |  | 78 (59 to 102) | 1.79 (1.35 to 2.34) | -2.36 (-2.82 to -1.89) |
| Lao People's Democratic Republic | 50 (27 to 79) | 2.42 (1.32 to 3.78) |  | 143 (84 to 219) | 3 (1.76 to 4.6) | 0.76 (0.69 to 0.83) |
| Latvia | 78 (67 to 92) | 4.07 (3.46 to 4.79) |  | 53 (38 to 73) | 3.77 (2.67 to 5.19) | -0.05 (-2.1 to 2.04) |
| Lebanon | 47 (30 to 69) | 2.6 (1.68 to 3.79) |  | 184 (120 to 271) | 4.87 (3.18 to 7.17) | 2.26 (2.04 to 2.48) |
| Lesotho | 8 (5 to 13) | 0.86 (0.52 to 1.42) |  | 27 (15 to 42) | 2.08 (1.19 to 3.24) | 3.33 (2.91 to 3.76) |
| Liberia | 9 (6 to 13) | 0.93 (0.61 to 1.35) |  | 28 (16 to 46) | 0.95 (0.53 to 1.52) | -0.12 (-0.4 to 0.17) |
| Libya | 54 (33 to 87) | 2.75 (1.68 to 4.35) |  | 211 (135 to 314) | 3.43 (2.2 to 5.12) | 0.6 (0.28 to 0.93) |
| Lithuania | 108 (92 to 127) | 4.2 (3.57 to 4.93) |  | 76 (54 to 104) | 3.75 (2.67 to 5.11) | -0.94 (-1.98 to 0.12) |
| Luxembourg | 15 (13 to 18) | 5.16 (4.26 to 6.21) |  | 21 (16 to 28) | 4.14 (3.08 to 5.43) | -0.9 (-1.14 to -0.66) |
| Madagascar | 82 (51 to 121) | 1.47 (0.94 to 2.16) |  | 199 (124 to 299) | 1.36 (0.84 to 2.05) | -0.43 (-0.66 to -0.21) |
| Malawi | 34 (23 to 48) | 0.76 (0.52 to 1.06) |  | 87 (52 to 137) | 0.94 (0.57 to 1.47) | 0.69 (0.48 to 0.9) |
| Malaysia | 278 (206 to 365) | 2.7 (2.02 to 3.53) |  | 984 (652 to 1420) | 4.34 (2.88 to 6.27) | 1.72 (1.38 to 2.05) |
| Maldives | 2 (1 to 2) | 1.67 (0.9 to 2.57) |  | 8 (5 to 11) | 1.84 (1.24 to 2.62) | 0.3 (0.12 to 0.47) |
| Mali | 42 (30 to 58) | 1.04 (0.74 to 1.42) |  | 120 (74 to 182) | 1.21 (0.75 to 1.82) | 0.58 (0.39 to 0.77) |
| Malta | 8 (6 to 10) | 2.66 (2.09 to 3.36) |  | 13 (9 to 17) | 3.95 (2.84 to 5.32) | 1.46 (1.11 to 1.82) |
| Marshall Islands | 1 (0 to 1) | 2.4 (1.63 to 3.36) |  | 1 (1 to 2) | 3.18 (1.91 to 4.89) | 0.96 (0.86 to 1.06) |
| Mauritania | 11 (8 to 17) | 1.18 (0.77 to 1.7) |  | 22 (13 to 36) | 1.04 (0.61 to 1.65) | -0.32 (-0.44 to -0.2) |
| Mauritius | 13 (11 to 16) | 1.91 (1.57 to 2.28) |  | 36 (26 to 48) | 3.62 (2.64 to 4.87) | 2.14 (1.89 to 2.39) |
| Mexico | 695 (662 to 729) | 1.48 (1.41 to 1.55) |  | 2960 (2506 to 3466) | 3.2 (2.71 to 3.75) | 3.01 (2.6 to 3.41) |
| Micronesia (Federated States of) | 1 (1 to 2) | 2.88 (1.76 to 4.3) |  | 2 (1 to 4) | 3.4 (0.98 to 5.68) | 0.5 (0.26 to 0.73) |
| Monaco | 1 (1 to 2) | 6.04 (4.18 to 8.55) |  | 2 (1 to 3) | 8.05 (5.21 to 11.93) | 1 (0.94 to 1.05) |
| Mongolia | 19 (13 to 26) | 1.82 (1.26 to 2.55) |  | 61 (40 to 89) | 2.27 (1.5 to 3.35) | 0.63 (0.41 to 0.85) |
| Montenegro | 15 (11 to 20) | 3.69 (2.75 to 4.82) |  | 19 (14 to 25) | 4.1 (3 to 5.41) | 0.22 (-0.24 to 0.68) |
| Morocco | 149 (104 to 207) | 1.11 (0.78 to 1.53) |  | 413 (256 to 653) | 1.55 (0.96 to 2.46) | 1.2 (1.09 to 1.32) |
| Mozambique | 43 (28 to 62) | 0.67 (0.44 to 0.97) |  | 176 (103 to 274) | 1.29 (0.75 to 2.02) | 2.38 (2.17 to 2.6) |
| Myanmar | 510 (307 to 781) | 2.26 (1.37 to 3.47) |  | 1231 (766 to 1857) | 3.1 (1.93 to 4.68) | 1.21 (0.98 to 1.45) |
| Namibia | 6 (3 to 9) | 0.92 (0.54 to 1.38) |  | 21 (12 to 33) | 1.42 (0.82 to 2.27) | 1.83 (1.38 to 2.27) |
| Nauru | 0 (0 to 1) | 5.15 (2.68 to 8.6) |  | 0 (0 to 1) | 5.36 (2.91 to 8.31) | 0.1 (-0.15 to 0.36) |
| Nepal | 76 (45 to 120) | 0.75 (0.44 to 1.17) |  | 175 (100 to 277) | 0.91 (0.53 to 1.44) | 0.75 (0.59 to 0.91) |
| Netherlands | 613 (534 to 707) | 5.26 (4.58 to 6.07) |  | 771 (546 to 1064) | 6.09 (4.3 to 8.41) | 0.42 (0.32 to 0.51) |
| New Zealand | 175 (149 to 206) | 7.21 (6.13 to 8.46) |  | 215 (161 to 278) | 6.75 (5.05 to 8.76) | -0.18 (-0.41 to 0.04) |
| Nicaragua | 25 (18 to 34) | 1.39 (1.01 to 1.86) |  | 100 (69 to 140) | 2.29 (1.59 to 3.2) | 1.59 (1.15 to 2.02) |
| Niger | 29 (19 to 44) | 0.79 (0.51 to 1.18) |  | 73 (43 to 117) | 0.77 (0.47 to 1.22) | -0.01 (-0.21 to 0.19) |
| Nigeria | 339 (227 to 492) | 0.76 (0.51 to 1.1) |  | 1065 (730 to 1504) | 0.98 (0.67 to 1.38) | 0.87 (0.65 to 1.1) |
| Niue | 0 (0 to 0) | 3.32 (2.07 to 4.96) |  | 0 (0 to 0) | 4.05 (2.08 to 6.84) | 0.75 (0.49 to 1.01) |
| North Macedonia | 46 (37 to 56) | 3.26 (2.62 to 4.02) |  | 89 (63 to 123) | 5.03 (3.55 to 6.96) | 1.52 (1.33 to 1.7) |
| Northern Mariana Islands | 2 (1 to 3) | 5.43 (3.31 to 8.35) |  | 2 (1 to 3) | 5.62 (3.51 to 8.63) | 0.02 (-0.34 to 0.38) |
| Norway | 160 (146 to 175) | 5.05 (4.6 to 5.54) |  | 218 (175 to 267) | 5.32 (4.26 to 6.52) | 0.15 (-0.2 to 0.5) |
| Oman | 13 (8 to 21) | 1.22 (0.71 to 1.93) |  | 66 (41 to 108) | 1.68 (1.06 to 2.74) | 0.99 (0.69 to 1.29) |
| Pakistan | 582 (431 to 767) | 1.07 (0.79 to 1.4) |  | 2459 (1708 to 3418) | 1.92 (1.34 to 2.66) | 2.05 (1.82 to 2.27) |
| Palau | 0 (0 to 0) | 2.98 (1.85 to 4.53) |  | 1 (0 to 1) | 3.4 (2.15 to 5.06) | 0.31 (0.09 to 0.53) |
| Palestine | 27 (16 to 41) | 3.2 (1.94 to 4.95) |  | 110 (80 to 147) | 3.87 (2.82 to 5.14) | 0.7 (0.48 to 0.93) |
| Panama | 26 (21 to 32) | 1.85 (1.48 to 2.29) |  | 95 (64 to 135) | 3.26 (2.18 to 4.63) | 1.93 (1.55 to 2.31) |
| Papua New Guinea | 31 (19 to 47) | 1.38 (0.84 to 2.11) |  | 104 (64 to 161) | 1.67 (1.03 to 2.58) | 0.64 (0.53 to 0.75) |
| Paraguay | 28 (21 to 35) | 1.26 (0.96 to 1.62) |  | 141 (92 to 206) | 3.03 (1.98 to 4.43) | 3.24 (2.68 to 3.8) |
| Peru | 229 (172 to 299) | 1.89 (1.43 to 2.44) |  | 930 (593 to 1382) | 3.8 (2.42 to 5.64) | 2.64 (1.95 to 3.33) |
| Philippines | 1340 (1160 to 1513) | 3.81 (3.3 to 4.29) |  | 3395 (2689 to 4305) | 4.53 (3.59 to 5.75) | 0.68 (0.51 to 0.85) |
| Poland | 967 (908 to 1028) | 3.66 (3.44 to 3.89) |  | 1322 (1072 to 1611) | 4.24 (3.44 to 5.17) | 0.2 (-0.16 to 0.57) |
| Portugal | 364 (309 to 426) | 5.25 (4.45 to 6.15) |  | 649 (443 to 910) | 7.46 (5.09 to 10.47) | 0.86 (0.46 to 1.25) |
| Puerto Rico | 91 (74 to 110) | 3.65 (2.98 to 4.41) |  | 158 (107 to 228) | 6.25 (4.24 to 8.99) | 1.62 (1.27 to 1.98) |
| Qatar | 5 (3 to 7) | 1.26 (0.82 to 1.89) |  | 60 (38 to 91) | 2.05 (1.3 to 3.15) | 1.79 (1.55 to 2.04) |
| Republic of Korea | 1010 (855 to 1185) | 3.25 (2.75 to 3.8) |  | 2413 (1771 to 3213) | 5.13 (3.75 to 6.85) | 1.31 (0.43 to 2.2) |
| Republic of Moldova | 149 (129 to 173) | 5.09 (4.4 to 5.89) |  | 130 (101 to 164) | 4.4 (3.39 to 5.55) | -0.58 (-1.56 to 0.41) |
| Romania | 562 (489 to 646) | 3.59 (3.12 to 4.13) |  | 891 (659 to 1177) | 5.67 (4.19 to 7.5) | 1.56 (0.62 to 2.51) |
| Russian Federation | 3927 (3561 to 4204) | 3.97 (3.61 to 4.25) |  | 6565 (5528 to 7718) | 5.67 (4.77 to 6.66) | 2.03 (-0.24 to 4.35) |
| Rwanda | 54 (33 to 80) | 1.74 (1.05 to 2.57) |  | 111 (67 to 174) | 1.57 (0.96 to 2.45) | -0.46 (-0.8 to -0.1) |
| Saint Kitts and Nevis | 1 (1 to 1) | 4.33 (3.48 to 5.3) |  | 2 (1 to 3) | 3.26 (1.01 to 5.36) | -0.88 (-1.21 to -0.55) |
| Saint Lucia | 2 (1 to 2) | 2.41 (1.98 to 2.9) |  | 5 (3 to 6) | 3.13 (2.37 to 4.05) | 0.75 (0.32 to 1.17) |
| Saint Vincent and the Grenadines | 1 (1 to 2) | 2.49 (2 to 3.07) |  | 3 (2 to 4) | 3.48 (2.69 to 4.44) | 0.95 (0.82 to 1.09) |
| Samoa | 2 (1 to 2) | 2.02 (1.3 to 3.04) |  | 3 (2 to 5) | 2.33 (1.3 to 3.64) | 0.46 (0.28 to 0.63) |
| San Marino | 1 (1 to 1) | 4.38 (3.12 to 6.02) |  | 1 (1 to 2) | 5.66 (3.66 to 8.33) | 0.91 (0.83 to 1) |
| Sao Tome and Principe | 1 (0 to 1) | 1.27 (0.72 to 1.89) |  | 3 (2 to 4) | 2.11 (1.2 to 3.43) | 1.93 (1.5 to 2.36) |
| Saudi Arabia | 96 (58 to 150) | 1.09 (0.67 to 1.7) |  | 1149 (730 to 1753) | 3.2 (2.04 to 4.87) | 3.83 (3.67 to 3.99) |
| Senegal | 38 (25 to 54) | 1.1 (0.73 to 1.56) |  | 97 (58 to 147) | 1.21 (0.73 to 1.83) | 0.19 (-0.26 to 0.65) |
| Serbia | 328 (238 to 441) | 4.86 (3.52 to 6.55) |  | 436 (304 to 609) | 6.64 (4.63 to 9.29) | 1.09 (0.85 to 1.33) |
| Seychelles | 1 (1 to 2) | 3.71 (2.74 to 4.93) |  | 6 (4 to 8) | 6.59 (4.72 to 8.95) | 2.06 (1.8 to 2.32) |
| Sierra Leone | 15 (10 to 23) | 0.82 (0.52 to 1.21) |  | 46 (28 to 69) | 1.02 (0.64 to 1.54) | 0.82 (0.43 to 1.21) |
| Singapore | 127 (104 to 152) | 5.25 (4.31 to 6.27) |  | 211 (150 to 287) | 3.94 (2.8 to 5.36) | -1.08 (-1.57 to -0.58) |
| Slovakia | 213 (177 to 255) | 5.75 (4.78 to 6.87) |  | 311 (210 to 444) | 6.74 (4.55 to 9.63) | 0.78 (0.27 to 1.29) |
| Slovenia | 73 (51 to 100) | 4.92 (3.43 to 6.77) |  | 85 (57 to 122) | 5.16 (3.5 to 7.44) | 0.01 (-0.28 to 0.29) |
| Solomon Islands | 4 (2 to 7) | 2.75 (1.36 to 4.48) |  | 15 (7 to 23) | 3.6 (1.84 to 5.6) | 1.01 (0.89 to 1.14) |
| Somalia | 34 (20 to 56) | 0.95 (0.56 to 1.57) |  | 90 (44 to 177) | 0.9 (0.44 to 1.75) | -0.16 (-0.21 to -0.11) |
| South Africa | 479 (404 to 562) | 2.26 (1.92 to 2.64) |  | 795 (561 to 1070) | 1.95 (1.38 to 2.62) | -0.71 (-0.92 to -0.49) |
| South Sudan | 40 (20 to 75) | 1.5 (0.75 to 2.82) |  | 72 (40 to 122) | 1.49 (0.82 to 2.53) | -0.16 (-0.52 to 0.2) |
| Spain | 1396 (1224 to 1588) | 5.29 (4.64 to 6.02) |  | 2281 (1588 to 3155) | 5.62 (3.92 to 7.77) | -0.11 (-0.3 to 0.08) |
| Sri Lanka | 126 (92 to 168) | 1.12 (0.82 to 1.48) |  | 285 (182 to 422) | 1.77 (1.13 to 2.62) | 1.79 (1.24 to 2.36) |
| Sudan | 102 (61 to 160) | 1.06 (0.64 to 1.65) |  | 371 (197 to 619) | 1.6 (0.85 to 2.65) | 1.4 (1.32 to 1.48) |
| Suriname | 5 (4 to 7) | 2.4 (1.72 to 3.17) |  | 15 (11 to 22) | 3.71 (2.56 to 5.18) | 1.41 (0.39 to 2.45) |
| Sweden | 267 (230 to 307) | 3.9 (3.35 to 4.51) |  | 322 (251 to 404) | 4.43 (3.45 to 5.57) | 0.49 (0.39 to 0.59) |
| Switzerland | 205 (173 to 244) | 3.69 (3.09 to 4.39) |  | 263 (183 to 367) | 3.87 (2.68 to 5.4) | -0.07 (-0.45 to 0.32) |
| Syrian Arab Republic | 100 (67 to 142) | 1.66 (1.12 to 2.35) |  | 179 (118 to 262) | 1.74 (1.15 to 2.55) | 0.43 (-0.11 to 0.97) |
| Taiwan (Province of China) | 816 (714 to 927) | 5.93 (5.2 to 6.73) |  | 2668 (1875 to 3719) | 13.19 (9.26 to 18.38) | 2.61 (2.04 to 3.19) |
| Tajikistan | 96 (76 to 121) | 3.79 (3 to 4.72) |  | 148 (101 to 211) | 2.37 (1.62 to 3.38) | -1.69 (-2.25 to -1.13) |
| Thailand | 986 (742 to 1292) | 2.66 (2.01 to 3.47) |  | 2312 (1516 to 3370) | 3.88 (2.54 to 5.66) | 1.09 (0.57 to 1.62) |
| Timor-Leste | 6 (4 to 10) | 1.43 (0.83 to 2.24) |  | 17 (5 to 28) | 2.38 (0.63 to 3.8) | 1.8 (1.45 to 2.15) |
| Togo | 18 (12 to 25) | 1.07 (0.73 to 1.51) |  | 56 (33 to 88) | 1.2 (0.71 to 1.87) | 0.38 (0.27 to 0.49) |
| Tokelau | 0 (0 to 0) | 2.22 (1.34 to 3.44) |  | 0 (0 to 0) | 2.94 (1.55 to 4.74) | 0.99 (0.92 to 1.06) |
| Tonga | 1 (0 to 1) | 1.12 (0.77 to 1.57) |  | 1 (1 to 1) | 1.41 (0.89 to 2.13) | 0.77 (0.64 to 0.9) |
| Trinidad and Tobago | 23 (19 to 28) | 3.07 (2.56 to 3.66) |  | 40 (27 to 58) | 3.8 (2.52 to 5.46) | 0.38 (-1.05 to 1.83) |
| Tunisia | 60 (42 to 85) | 1.37 (0.95 to 1.91) |  | 214 (135 to 319) | 2.33 (1.47 to 3.48) | 1.79 (1.68 to 1.9) |
| Turkey | 1080 (705 to 1570) | 3.19 (2.09 to 4.62) |  | 2445 (1719 to 3360) | 3.7 (2.6 to 5.08) | 0.41 (-0.25 to 1.07) |
| Turkmenistan | 52 (43 to 62) | 2.76 (2.31 to 3.28) |  | 78 (56 to 106) | 2.15 (1.55 to 2.93) | -0.46 (-1.4 to 0.49) |
| Tuvalu | 0 (0 to 0) | 2.39 (1.57 to 3.49) |  | 0 (0 to 0) | 2.96 (1.75 to 4.62) | 0.71 (0.62 to 0.8) |
| Uganda | 71 (44 to 105) | 1 (0.63 to 1.47) |  | 397 (252 to 599) | 2.14 (1.37 to 3.21) | 2.62 (2.47 to 2.77) |
| Ukraine | 2186 (1912 to 2481) | 6.14 (5.37 to 6.97) |  | 2040 (1551 to 2635) | 5.8 (4.4 to 7.5) | 0.36 (-0.78 to 1.5) |
| United Arab Emirates | 24 (14 to 39) | 1.71 (1 to 2.76) |  | 289 (172 to 457) | 2.12 (1.26 to 3.33) | 0.72 (0.47 to 0.97) |
| United Kingdom | 1973 (1899 to 2051) | 4.67 (4.49 to 4.85) |  | 2715 (2220 to 3294) | 5.54 (4.53 to 6.71) | 0.65 (0.48 to 0.82) |
| United Republic of Tanzania | 148 (97 to 221) | 1.3 (0.86 to 1.93) |  | 491 (301 to 766) | 1.67 (1.03 to 2.6) | 0.83 (0.74 to 0.93) |
| United States of America | 10550 (10136 to 10969) | 5.63 (5.41 to 5.85) |  | 16780 (14177 to 19860) | 7.23 (6.12 to 8.56) | 1.1 (0.94 to 1.25) |
| United States Virgin Islands | 3 (2 to 5) | 4.07 (2.85 to 5.64) |  | 4 (3 to 7) | 5.83 (3.74 to 9.05) | 1.21 (0.94 to 1.47) |
| Uruguay | 90 (74 to 106) | 4.39 (3.64 to 5.2) |  | 124 (85 to 174) | 5.06 (3.49 to 7.09) | 0.32 (0.08 to 0.56) |
| Uzbekistan | 286 (246 to 331) | 2.73 (2.36 to 3.13) |  | 582 (448 to 745) | 2.45 (1.88 to 3.13) | -0.22 (-0.58 to 0.13) |
| Vanuatu | 1 (1 to 2) | 1.66 (0.95 to 2.62) |  | 4 (2 to 6) | 2.26 (1.28 to 3.49) | 1.04 (0.76 to 1.31) |
| Venezuela (Bolivarian Republic of) | 207 (178 to 240) | 1.93 (1.67 to 2.23) |  | 715 (495 to 1012) | 3.4 (2.35 to 4.82) | 2.04 (1.21 to 2.89) |
| Viet Nam | 530 (348 to 772) | 1.68 (1.11 to 2.43) |  | 3506 (2243 to 5217) | 4.43 (2.83 to 6.59) | 3.44 (3.35 to 3.52) |
| Yemen | 58 (30 to 101) | 1.04 (0.54 to 1.79) |  | 233 (136 to 366) | 1.37 (0.8 to 2.14) | 1.07 (0.93 to 1.21) |
| Zambia | 64 (39 to 95) | 1.87 (1.16 to 2.76) |  | 241 (144 to 369) | 2.54 (1.52 to 3.91) | 1.09 (0.96 to 1.21) |
| Zimbabwe | 67 (48 to 90) | 1.49 (1.08 to 2.01) |  | 209 (128 to 317) | 2.49 (1.53 to 3.76) | 1.66 (1.22 to 2.1) |

UI: uncertainty interval, CI: confidence interval, AAPC, average annual percent change.
