# Supplementary material for: The global, regional, and national early-onset colorectal cancer burden and trends from 1990 to 2019: results from the Global Burden of Disease Study 2019
Source: BMC Public Health. 2022 Oct 12;22:1896. doi: 10.1186/s12889-022-14274-7 (PMC9555189; doi:10.1186/s12889-022-14274-7)
Supplement: Supplementary file 7 — Additional file 7: Table S2. Prevalence of early-onset colorectal cancer in 1990 and 2019 with AAPC from 2009 and 2019 at countries/territories level, both sexes. [file 12889_2022_14274_MOESM7_ESM.docx]

Table S2. Prevalence of early-onset colorectal cancer in 1990 and 2019 with AAPC from 2009 and 2019 at countries/territories level, both sexes.

| Countries/territories | 1990 | |  | 2019 | | AAPC % (95% CI)  1990-2019 |
| --- | --- | --- | --- | --- | --- | --- |
|  | Cases (95% UI) | Age-standardized prevalence per  100 000 population (95% UI) |  | Cases (95% UI) | Age-standardized prevalence per  100 000 population (95% UI) |  |
| Afghanistan | 355 (148 to 645) | 6.71 (2.76 to 12.27) |  | 1480 (775 to 2385) | 7.4 (3.84 to 11.94) | 0.35 (0.27 to 0.44) |
| Albania | 229 (179 to 287) | 11.03 (8.71 to 13.7) |  | 408 (272 to 589) | 22.61 (14.99 to 32.62) | 2.56 (2.44 to 2.69) |
| Algeria | 872 (616 to 1204) | 7.25 (5.16 to 9.94) |  | 3209 (2162 to 4606) | 10.11 (6.81 to 14.52) | 1.2 (1.04 to 1.36) |
| American Samoa | 5 (3 to 6) | 16.25 (11.15 to 22.95) |  | 8 (5 to 11) | 21.29 (13.98 to 31.69) | 1.12 (0.87 to 1.37) |
| Andorra | 18 (11 to 27) | 40.51 (25.37 to 61.07) |  | 40 (26 to 59) | 49.54 (31.35 to 73.07) | 0.69 (0.58 to 0.8) |
| Angola | 246 (145 to 393) | 4.72 (2.82 to 7.55) |  | 963 (586 to 1447) | 6.36 (3.91 to 9.54) | 1.07 (0.99 to 1.15) |
| Antigua and Barbuda | 4 (3 to 6) | 11.95 (9.32 to 15.2) |  | 12 (9 to 17) | 16.89 (12.26 to 22.92) | 1.12 (0.99 to 1.25) |
| Argentina | 3484 (3025 to 4005) | 16.21 (14.08 to 18.62) |  | 9490 (6742 to 12888) | 28.8 (20.46 to 39.12) | 2.08 (1.91 to 2.24) |
| Armenia | 404 (335 to 480) | 20.2 (16.87 to 23.91) |  | 446 (339 to 572) | 20.56 (15.65 to 26.38) | -0.31 (-0.64 to 0.02) |
| Australia | 5212 (4599 to 5871) | 41.29 (36.45 to 46.52) |  | 9642 (6871 to 13241) | 52.51 (37.39 to 72.1) | 1.19 (0.91 to 1.46) |
| Austria | 2695 (2272 to 3149) | 45.84 (38.6 to 53.61) |  | 1931 (1397 to 2600) | 27.95 (20.21 to 37.7) | -1.9 (-2.12 to -1.68) |
| Azerbaijan | 826 (663 to 1022) | 20.1 (16.3 to 24.7) |  | 1588 (1112 to 2254) | 20.25 (14.17 to 28.76) | 0.14 (-0.37 to 0.66) |
| Bahamas | 35 (28 to 44) | 21.94 (17.6 to 27.06) |  | 94 (69 to 128) | 31.1 (22.71 to 42.21) | 1.12 (0.88 to 1.36) |
| Bahrain | 30 (21 to 41) | 8.87 (6.33 to 12.07) |  | 244 (162 to 357) | 15.12 (10.02 to 22.13) | 2.05 (1.71 to 2.39) |
| Bangladesh | 1604 (995 to 2393) | 2.94 (1.86 to 4.35) |  | 4333 (2559 to 7012) | 3.91 (2.31 to 6.31) | 0.95 (0.86 to 1.04) |
| Barbados | 35 (29 to 43) | 21.26 (17.42 to 25.61) |  | 73 (54 to 98) | 32.75 (24.03 to 44.14) | 1.46 (1.36 to 1.57) |
| Belarus | 1920 (1631 to 2249) | 28.38 (24.14 to 33.22) |  | 2227 (1510 to 3117) | 30.31 (20.55 to 42.48) | -0.07 (-0.29 to 0.15) |
| Belgium | 2103 (1765 to 2485) | 29.75 (24.97 to 35.16) |  | 2540 (1773 to 3512) | 30.2 (21.09 to 41.82) | -0.2 (-0.34 to -0.06) |
| Belize | 5 (4 to 6) | 5.33 (4.22 to 6.55) |  | 40 (31 to 52) | 14.34 (10.94 to 18.45) | 3.22 (2.92 to 3.52) |
| Benin | 76 (52 to 107) | 3.46 (2.38 to 4.83) |  | 282 (176 to 434) | 4.4 (2.78 to 6.71) | 0.79 (0.71 to 0.87) |
| Bermuda | 13 (10 to 17) | 27.52 (21.12 to 35.26) |  | 20 (14 to 27) | 38.99 (27.28 to 54.02) | 1.01 (0.76 to 1.26) |
| Bhutan | 9 (5 to 16) | 3.07 (1.54 to 5.16) |  | 29 (13 to 49) | 5.44 (2.52 to 9.08) | 1.97 (1.89 to 2.04) |
| Bolivia (Plurinational State of) | 218 (144 to 307) | 6.29 (4.16 to 8.82) |  | 916 (530 to 1418) | 11.81 (6.87 to 18.22) | 2.28 (2.12 to 2.45) |
| Bosnia and Herzegovina | 512 (417 to 627) | 16.32 (13.3 to 19.94) |  | 829 (587 to 1135) | 33.15 (23.39 to 45.39) | 2.52 (2.3 to 2.75) |
| Botswana | 44 (27 to 69) | 7.08 (4.36 to 11.02) |  | 250 (139 to 410) | 14.76 (8.26 to 24.14) | 2.52 (2.3 to 2.74) |
| Brazil | 9039 (8462 to 9655) | 9.95 (9.32 to 10.61) |  | 31395 (28797 to 34124) | 18.64 (17.1 to 20.27) | 2.18 (2.12 to 2.24) |
| Brunei Darussalam | 49 (35 to 67) | 29.6 (21.06 to 40.62) |  | 162 (116 to 222) | 42.58 (30.56 to 58.34) | 1.28 (0.89 to 1.68) |
| Bulgaria | 1918 (1631 to 2239) | 29.78 (25.26 to 34.84) |  | 2189 (1527 to 3030) | 39.1 (27.29 to 54.17) | 1.03 (0.73 to 1.32) |
| Burkina Faso | 129 (85 to 189) | 2.98 (1.97 to 4.34) |  | 493 (315 to 730) | 4.31 (2.78 to 6.32) | 1.34 (1.1 to 1.58) |
| Burundi | 137 (87 to 203) | 5.37 (3.42 to 7.98) |  | 294 (182 to 460) | 4.99 (3.08 to 7.81) | -0.3 (-0.41 to -0.2) |
| Cabo Verde | 6 (5 to 9) | 4.49 (3.22 to 6.05) |  | 34 (22 to 51) | 8.72 (5.69 to 13.1) | 2.4 (2.3 to 2.5) |
| Cambodia | 389 (244 to 572) | 7.72 (4.9 to 11.28) |  | 1635 (1086 to 2423) | 15 (9.97 to 22.22) | 2.22 (2.01 to 2.42) |
| Cameroon | 255 (175 to 353) | 5.13 (3.54 to 7.09) |  | 1087 (646 to 1707) | 6.85 (4.1 to 10.75) | 1.03 (0.88 to 1.19) |
| Canada | 8267 (7154 to 9534) | 39.29 (34.01 to 45.34) |  | 13148 (9222 to 18237) | 50.08 (35.11 to 69.51) | 1 (0.89 to 1.1) |
| Central African Republic | 63 (39 to 94) | 4.68 (2.89 to 6.91) |  | 123 (73 to 194) | 4.26 (2.54 to 6.72) | -0.32 (-0.38 to -0.27) |
| Chad | 77 (50 to 110) | 2.79 (1.85 to 3.98) |  | 285 (181 to 424) | 3.88 (2.49 to 5.75) | 1.16 (1.09 to 1.23) |
| Chile | 914 (763 to 1084) | 10.54 (8.82 to 12.47) |  | 3069 (2151 to 4239) | 22.38 (15.69 to 30.89) | 2.73 (2.54 to 2.92) |
| China | 122167 (105478 to 141442) | 15.3 (13.22 to 17.7) |  | 591944 (489497 to 705006) | 49.54 (41.06 to 58.91) | 4.13 (3.91 to 4.36) |
| Colombia | 1937 (1648 to 2264) | 9.8 (8.37 to 11.41) |  | 7696 (5244 to 10932) | 22.56 (15.37 to 32.05) | 2.74 (2.14 to 3.34) |
| Comoros | 10 (3 to 17) | 4.56 (1.35 to 7.67) |  | 29 (16 to 45) | 6.17 (3.36 to 9.54) | 0.62 (-0.24 to 1.49) |
| Congo | 88 (47 to 142) | 7.84 (4.24 to 12.55) |  | 263 (155 to 411) | 7.67 (4.51 to 11.96) | -0.07 (-0.14 to 0.01) |
| Cook Islands | 1 (1 to 2) | 11.57 (7.5 to 17.28) |  | 2 (1 to 3) | 15.3 (8.36 to 23.44) | 1 (0.95 to 1.05) |
| Costa Rica | 245 (198 to 297) | 14.05 (11.41 to 17) |  | 1223 (855 to 1723) | 35.32 (24.69 to 49.78) | 3.4 (3.25 to 3.56) |
| Croatia | 1085 (894 to 1299) | 29.88 (24.62 to 35.82) |  | 1273 (877 to 1798) | 40 (27.57 to 56.46) | 0.92 (0.67 to 1.16) |
| Cuba | 1504 (1265 to 1787) | 19.79 (16.66 to 23.47) |  | 2529 (1847 to 3421) | 27.64 (20.14 to 37.56) | 1.25 (1.14 to 1.37) |
| Cyprus | 70 (52 to 93) | 12.59 (9.26 to 16.59) |  | 306 (221 to 411) | 27.98 (20.15 to 37.61) | 2.52 (2.2 to 2.84) |
| Czechia | 2898 (2520 to 3347) | 35.25 (30.59 to 40.77) |  | 2903 (2086 to 3900) | 31.37 (22.53 to 42.18) | -0.68 (-0.84 to -0.51) |
| C么te d'Ivoire | 298 (197 to 436) | 4.98 (3.31 to 7.27) |  | 831 (515 to 1246) | 5.41 (3.37 to 8.07) | 0.21 (0.12 to 0.29) |
| Democratic People's Republic of Korea | 2907 (1783 to 4441) | 20.52 (12.59 to 31.35) |  | 4593 (2650 to 7485) | 21.35 (12.25 to 34.87) | -0.02 (-0.26 to 0.22) |
| Democratic Republic of the Congo | 670 (433 to 1001) | 3.77 (2.48 to 5.58) |  | 1725 (1035 to 2744) | 3.81 (2.29 to 6.01) | 0.04 (-0.02 to 0.1) |
| Denmark | 1152 (988 to 1337) | 27.61 (23.61 to 32.14) |  | 1440 (1016 to 1981) | 34.52 (24.34 to 47.57) | 0.7 (0.29 to 1.1) |
| Djibouti | 12 (7 to 19) | 5.38 (3.21 to 8.28) |  | 67 (37 to 111) | 8.02 (4.48 to 13.2) | 1.27 (1.01 to 1.52) |
| Dominica | 5 (4 to 6) | 12.21 (8.9 to 16.18) |  | 8 (5 to 11) | 16.06 (10.79 to 22.89) | 0.92 (0.84 to 1.01) |
| Dominican Republic | 289 (216 to 381) | 7.2 (5.42 to 9.43) |  | 1412 (881 to 2119) | 18.51 (11.56 to 27.78) | 3.35 (2.99 to 3.72) |
| Ecuador | 396 (321 to 485) | 6.97 (5.69 to 8.49) |  | 2583 (1752 to 3655) | 21.54 (14.62 to 30.46) | 3.8 (3.61 to 3.99) |
| Egypt | 2002 (1511 to 2632) | 6.14 (4.7 to 7.99) |  | 7225 (4464 to 11002) | 11.05 (6.82 to 16.82) | 2.03 (1.85 to 2.2) |
| El Salvador | 165 (131 to 205) | 5.88 (4.69 to 7.25) |  | 796 (526 to 1165) | 19 (12.57 to 27.8) | 4.19 (3.83 to 4.55) |
| Equatorial Guinea | 8 (5 to 13) | 3.96 (2.39 to 6.2) |  | 71 (36 to 125) | 9.81 (4.99 to 17.24) | 3.27 (3.13 to 3.41) |
| Eritrea | 61 (37 to 91) | 4.28 (2.62 to 6.37) |  | 277 (172 to 426) | 7.37 (4.6 to 11.26) | 1.77 (1.49 to 2.05) |
| Estonia | 290 (238 to 350) | 26.14 (21.44 to 31.63) |  | 359 (245 to 509) | 36.2 (24.68 to 51.32) | 1.08 (0.88 to 1.28) |
| Eswatini | 21 (14 to 32) | 5.82 (3.87 to 8.57) |  | 70 (38 to 117) | 10.44 (5.65 to 17.42) | 1.99 (1.86 to 2.13) |
| Ethiopia | 1339 (816 to 2005) | 5.68 (3.5 to 8.49) |  | 3011 (2131 to 4262) | 5.58 (3.94 to 7.91) | -0.14 (-0.25 to -0.04) |
| Fiji | 44 (31 to 62) | 9.34 (6.59 to 13) |  | 77 (52 to 110) | 12.04 (8.13 to 17.19) | 0.9 (0.85 to 0.95) |
| Finland | 995 (836 to 1175) | 23.96 (20.11 to 28.38) |  | 1025 (705 to 1422) | 27.68 (19.03 to 38.47) | 0.58 (0.31 to 0.84) |
| France | 10969 (9508 to 12615) | 26.86 (23.29 to 30.88) |  | 16214 (11245 to 22565) | 34.12 (23.7 to 47.43) | 0.69 (0.56 to 0.81) |
| Gabon | 44 (24 to 76) | 9.47 (5.22 to 16.19) |  | 120 (70 to 190) | 10.7 (6.25 to 17) | 0.39 (0.22 to 0.57) |
| Gambia | 12 (7 to 18) | 2.58 (1.63 to 3.85) |  | 45 (27 to 70) | 3.8 (2.28 to 5.95) | 1.43 (0.91 to 1.94) |
| Georgia | 827 (662 to 1027) | 19.82 (15.9 to 24.62) |  | 619 (447 to 834) | 15.99 (11.55 to 21.56) | -1.07 (-1.61 to -0.53) |
| Germany | 19041 (16536 to 21757) | 31.95 (27.72 to 36.54) |  | 21873 (15470 to 30528) | 37.18 (26.32 to 51.85) | 0.08 (-0.31 to 0.46) |
| Ghana | 357 (240 to 509) | 4.51 (3.06 to 6.4) |  | 1451 (894 to 2184) | 7.17 (4.46 to 10.75) | 1.57 (1.45 to 1.69) |
| Greece | 1434 (1212 to 1691) | 19.58 (16.52 to 23.11) |  | 2408 (1751 to 3264) | 28.9 (20.98 to 39.24) | 1.12 (0.95 to 1.29) |
| Greenland | 12 (9 to 17) | 28.78 (20.66 to 39.03) |  | 14 (10 to 21) | 38 (26.46 to 54.38) | 0.81 (0.64 to 0.98) |
| Grenada | 6 (4 to 8) | 13.78 (10.52 to 17.87) |  | 16 (12 to 22) | 21.29 (15.44 to 28.64) | 1.41 (1.21 to 1.62) |
| Guam | 19 (13 to 27) | 20.57 (14.26 to 29.12) |  | 34 (23 to 49) | 29.75 (20.15 to 42.57) | 1.32 (1.15 to 1.49) |
| Guatemala | 185 (149 to 228) | 4.6 (3.71 to 5.65) |  | 1455 (1052 to 1957) | 12.97 (9.38 to 17.41) | 3.71 (3.32 to 4.11) |
| Guinea | 90 (63 to 125) | 2.94 (2.06 to 4.05) |  | 257 (165 to 386) | 4.08 (2.63 to 6.11) | 1.19 (1.13 to 1.25) |
| Guinea-Bissau | 25 (15 to 38) | 5.29 (3.27 to 7.99) |  | 60 (38 to 89) | 5.93 (3.74 to 8.77) | 0.36 (0.3 to 0.42) |
| Guyana | 45 (33 to 58) | 10.3 (7.67 to 13.32) |  | 99 (67 to 143) | 18.6 (12.49 to 26.95) | 2.22 (1.67 to 2.76) |
| Haiti | 240 (139 to 363) | 7.04 (4.14 to 10.58) |  | 616 (360 to 962) | 7.74 (4.54 to 12.1) | 0.45 (0.04 to 0.86) |
| Honduras | 123 (86 to 170) | 5.33 (3.78 to 7.33) |  | 435 (240 to 736) | 7.31 (4.07 to 12.29) | 1.02 (0.83 to 1.22) |
| Hungary | 2634 (2250 to 3068) | 33.16 (28.25 to 38.7) |  | 3413 (2487 to 4543) | 41.48 (30.23 to 55.3) | 0.57 (0.23 to 0.92) |
| Iceland | 42 (34 to 53) | 24.68 (19.55 to 30.7) |  | 68 (51 to 88) | 27.28 (20.57 to 35.38) | 0.26 (-0.02 to 0.54) |
| India | 21234 (18122 to 24618) | 4.17 (3.58 to 4.82) |  | 67661 (55689 to 80411) | 6.87 (5.65 to 8.16) | 1.79 (1.57 to 2.01) |
| Indonesia | 9775 (7114 to 12201) | 8.85 (6.49 to 11.01) |  | 33166 (23618 to 43726) | 16.04 (11.44 to 21.14) | 2.01 (1.95 to 2.08) |
| Iran (Islamic Republic of) | 2289 (1825 to 2867) | 8.15 (6.53 to 10.2) |  | 11272 (9712 to 13073) | 16.03 (13.81 to 18.62) | 2.31 (2.21 to 2.41) |
| Iraq | 587 (364 to 924) | 6.98 (4.36 to 10.94) |  | 3515 (2278 to 5359) | 12.66 (8.21 to 19.29) | 2.26 (1.91 to 2.62) |
| Ireland | 752 (618 to 899) | 31.83 (26.19 to 38.04) |  | 1403 (967 to 1962) | 35.28 (24.31 to 49.36) | 0.3 (-0.1 to 0.71) |
| Israel | 691 (569 to 836) | 22.54 (18.61 to 27.16) |  | 1974 (1383 to 2747) | 30.88 (21.63 to 42.96) | 1.03 (0.84 to 1.21) |
| Italy | 15274 (13554 to 17130) | 36.74 (32.6 to 41.21) |  | 19518 (15329 to 24290) | 39.6 (31.16 to 49.25) | 0.15 (-0.01 to 0.31) |
| Jamaica | 101 (78 to 127) | 8.21 (6.43 to 10.3) |  | 500 (340 to 711) | 24.66 (16.74 to 35.08) | 4.69 (3.74 to 5.64) |
| Japan | 46609 (43634 to 49821) | 43.4 (40.62 to 46.42) |  | 49430 (40386 to 59498) | 49.54 (40.52 to 59.47) | 0.4 (0.3 to 0.5) |
| Jordan | 191 (135 to 264) | 11.41 (8.08 to 15.65) |  | 1496 (1061 to 2069) | 18.62 (13.22 to 25.72) | 1.81 (1.69 to 1.93) |
| Kazakhstan | 1831 (1570 to 2113) | 18.57 (15.98 to 21.36) |  | 2362 (1848 to 2975) | 17.35 (13.57 to 21.85) | -0.32 (-0.57 to -0.07) |
| Kenya | 343 (240 to 433) | 3.46 (2.4 to 4.35) |  | 1664 (1265 to 2153) | 5.88 (4.48 to 7.58) | 1.74 (1.6 to 1.88) |
| Kiribati | 5 (3 to 7) | 11.55 (7.93 to 16.63) |  | 9 (6 to 14) | 11.8 (7.54 to 18.17) | 0.09 (0.04 to 0.14) |
| Kuwait | 106 (81 to 136) | 8.69 (6.61 to 11.08) |  | 648 (455 to 897) | 13.95 (9.77 to 19.35) | 1.52 (1.08 to 1.96) |
| Kyrgyzstan | 363 (293 to 445) | 15.52 (12.59 to 18.94) |  | 426 (322 to 552) | 9.77 (7.38 to 12.65) | -1.91 (-2.27 to -1.55) |
| Lao People's Democratic Republic | 190 (107 to 295) | 9.01 (5.1 to 13.91) |  | 637 (378 to 972) | 13.24 (7.89 to 20.23) | 1.32 (1.27 to 1.37) |
| Latvia | 425 (358 to 504) | 22.16 (18.64 to 26.35) |  | 338 (241 to 462) | 23.99 (17.1 to 32.89) | 0.25 (-0.77 to 1.28) |
| Lebanon | 229 (150 to 333) | 12.56 (8.24 to 18.2) |  | 1170 (758 to 1734) | 30.95 (20.07 to 45.88) | 3.26 (3.07 to 3.45) |
| Lesotho | 31 (19 to 50) | 3.39 (2.11 to 5.5) |  | 103 (60 to 161) | 7.91 (4.63 to 12.31) | 3.2 (2.9 to 3.5) |
| Liberia | 34 (23 to 50) | 3.42 (2.31 to 4.96) |  | 117 (67 to 186) | 3.87 (2.22 to 6.17) | 0.44 (0.35 to 0.53) |
| Libya | 256 (157 to 403) | 12.71 (7.86 to 19.92) |  | 1113 (714 to 1659) | 18.16 (11.64 to 27.07) | 1.04 (0.87 to 1.21) |
| Lithuania | 603 (506 to 716) | 23.41 (19.64 to 27.82) |  | 476 (339 to 645) | 23.54 (16.76 to 31.99) | -0.33 (-1.12 to 0.47) |
| Luxembourg | 97 (80 to 117) | 32.74 (26.88 to 39.65) |  | 156 (117 to 204) | 30.28 (22.65 to 39.47) | -0.41 (-0.53 to -0.28) |
| Madagascar | 311 (198 to 457) | 5.46 (3.54 to 7.91) |  | 788 (500 to 1175) | 5.32 (3.37 to 7.95) | -0.18 (-0.41 to 0.05) |
| Malawi | 130 (89 to 181) | 2.84 (1.97 to 3.93) |  | 355 (216 to 557) | 3.76 (2.31 to 5.85) | 0.95 (0.87 to 1.02) |
| Malaysia | 1357 (1017 to 1769) | 13.08 (9.87 to 16.98) |  | 5849 (3911 to 8389) | 25.77 (17.25 to 36.96) | 2.31 (2.19 to 2.43) |
| Maldives | 7 (4 to 11) | 7.5 (4.25 to 11.34) |  | 51 (34 to 73) | 11.7 (7.94 to 16.64) | 1.56 (1.05 to 2.08) |
| Mali | 157 (113 to 215) | 3.78 (2.73 to 5.13) |  | 490 (306 to 739) | 4.83 (3.04 to 7.22) | 0.86 (0.67 to 1.04) |
| Malta | 50 (40 to 63) | 17.18 (13.61 to 21.6) |  | 91 (66 to 122) | 28.05 (20.36 to 37.62) | 1.66 (1.48 to 1.84) |
| Marshall Islands | 2 (1 to 3) | 10.01 (6.9 to 13.87) |  | 6 (3 to 8) | 13.99 (8.49 to 21.14) | 1.17 (1.01 to 1.32) |
| Mauritania | 44 (29 to 63) | 4.45 (2.95 to 6.35) |  | 100 (60 to 158) | 4.61 (2.77 to 7.24) | 0.19 (0.11 to 0.27) |
| Mauritius | 72 (59 to 87) | 10.12 (8.31 to 12.18) |  | 213 (155 to 286) | 21.43 (15.57 to 28.84) | 2.63 (2.45 to 2.81) |
| Mexico | 3492 (3307 to 3688) | 7.32 (6.94 to 7.72) |  | 17261 (14640 to 20230) | 18.67 (15.84 to 21.88) | 3.48 (3.34 to 3.61) |
| Micronesia (Federated States of) | 6 (4 to 9) | 11.94 (7.52 to 17.5) |  | 11 (4 to 18) | 16 (5.08 to 26.36) | 0.9 (0.62 to 1.17) |
| Monaco | 10 (7 to 14) | 40.75 (28.33 to 57.53) |  | 15 (10 to 22) | 57.43 (37.36 to 84.81) | 1.19 (1.15 to 1.22) |
| Mongolia | 81 (57 to 113) | 7.72 (5.49 to 10.7) |  | 299 (200 to 436) | 11.2 (7.5 to 16.35) | 1.2 (1.07 to 1.33) |
| Montenegro | 91 (68 to 120) | 21.88 (16.29 to 28.72) |  | 124 (92 to 164) | 26.53 (19.53 to 35.14) | 0.63 (0.48 to 0.77) |
| Morocco | 622 (437 to 862) | 4.56 (3.22 to 6.28) |  | 2015 (1271 to 3183) | 7.59 (4.79 to 11.99) | 1.74 (1.7 to 1.78) |
| Mozambique | 159 (107 to 226) | 2.45 (1.66 to 3.48) |  | 683 (406 to 1052) | 4.94 (2.93 to 7.61) | 2.59 (2.35 to 2.83) |
| Myanmar | 2023 (1244 to 3060) | 8.82 (5.47 to 13.32) |  | 5744 (3596 to 8659) | 14.49 (9.07 to 21.84) | 1.84 (1.59 to 2.09) |
| Namibia | 24 (14 to 36) | 3.53 (2.13 to 5.24) |  | 93 (54 to 148) | 6.32 (3.69 to 10.06) | 2.29 (1.93 to 2.65) |
| Nauru | 1 (1 to 2) | 24.04 (12.95 to 39.67) |  | 2 (1 to 3) | 27.13 (15.08 to 41.79) | 0.38 (0.2 to 0.56) |
| Nepal | 280 (166 to 441) | 2.7 (1.6 to 4.24) |  | 764 (439 to 1213) | 3.97 (2.29 to 6.27) | 1.3 (1.08 to 1.52) |
| Netherlands | 4097 (3516 to 4780) | 35.14 (30.14 to 41.01) |  | 5583 (3962 to 7623) | 44.23 (31.38 to 60.55) | 0.76 (0.54 to 0.98) |
| New Zealand | 1149 (966 to 1358) | 47.15 (39.66 to 55.67) |  | 1544 (1165 to 1993) | 48.58 (36.64 to 62.87) | 0.16 (0.04 to 0.27) |
| Nicaragua | 124 (89 to 167) | 6.61 (4.86 to 8.8) |  | 588 (405 to 828) | 13.43 (9.27 to 18.84) | 2.65 (2.48 to 2.82) |
| Niger | 108 (70 to 160) | 2.87 (1.88 to 4.22) |  | 295 (179 to 471) | 3.03 (1.88 to 4.79) | 0.25 (0.09 to 0.41) |
| Nigeria | 1372 (946 to 1928) | 3.02 (2.09 to 4.23) |  | 4571 (3174 to 6427) | 4.15 (2.89 to 5.82) | 1.09 (1.07 to 1.12) |
| Niue | 0 (0 to 0) | 16.81 (10.72 to 25.03) |  | 0 (0 to 0) | 23.4 (12.43 to 39.32) | 1.13 (1.05 to 1.21) |
| North Macedonia | 237 (189 to 293) | 16.9 (13.54 to 20.92) |  | 544 (385 to 753) | 30.95 (21.9 to 42.9) | 2.25 (2.04 to 2.45) |
| Northern Mariana Islands | 11 (7 to 17) | 30.61 (18.95 to 46.62) |  | 11 (7 to 17) | 34.51 (21.79 to 52.75) | 0.29 (-0.01 to 0.59) |
| Norway | 1046 (932 to 1168) | 33.05 (29.47 to 36.92) |  | 1586 (1274 to 1941) | 38.77 (31.14 to 47.43) | 0.53 (0.26 to 0.8) |
| Oman | 69 (43 to 106) | 6.32 (3.87 to 9.67) |  | 433 (276 to 699) | 10.91 (6.96 to 17.62) | 1.58 (1.27 to 1.89) |
| Pakistan | 2297 (1720 to 3010) | 4.15 (3.13 to 5.41) |  | 10501 (7348 to 14550) | 8.11 (5.69 to 11.21) | 2.31 (2.21 to 2.4) |
| Palau | 2 (1 to 2) | 15.77 (10 to 23.76) |  | 3 (2 to 5) | 19.73 (12.64 to 29.42) | 0.65 (0.48 to 0.81) |
| Palestine | 126 (77 to 194) | 14.75 (9.06 to 22.54) |  | 602 (436 to 812) | 21.01 (15.25 to 28.25) | 1.27 (1.16 to 1.38) |
| Panama | 139 (111 to 174) | 9.76 (7.81 to 12.12) |  | 578 (386 to 824) | 19.83 (13.26 to 28.29) | 2.49 (2.27 to 2.72) |
| Papua New Guinea | 125 (77 to 189) | 5.45 (3.4 to 8.22) |  | 442 (278 to 679) | 6.98 (4.39 to 10.73) | 0.84 (0.79 to 0.88) |
| Paraguay | 128 (96 to 165) | 5.78 (4.36 to 7.42) |  | 762 (495 to 1120) | 16.33 (10.61 to 23.98) | 3.7 (3.61 to 3.78) |
| Peru | 1242 (934 to 1631) | 10.11 (7.67 to 13.18) |  | 6323 (4025 to 9421) | 25.81 (16.43 to 38.46) | 3.26 (2.87 to 3.66) |
| Philippines | 6238 (5356 to 7145) | 17.53 (15.09 to 20.07) |  | 16930 (13385 to 21431) | 22.53 (17.8 to 28.54) | 0.87 (0.76 to 0.97) |
| Poland | 4682 (4278 to 5109) | 17.66 (16.15 to 19.25) |  | 7963 (6377 to 9772) | 25.63 (20.54 to 31.44) | 1.17 (1.05 to 1.28) |
| Portugal | 2230 (1870 to 2639) | 32.18 (26.96 to 38.11) |  | 4632 (3182 to 6486) | 53.44 (36.66 to 74.86) | 1.36 (1.07 to 1.66) |
| Puerto Rico | 525 (429 to 639) | 21.11 (17.25 to 25.69) |  | 1051 (714 to 1515) | 41.59 (28.26 to 59.8) | 2.24 (1.95 to 2.52) |
| Qatar | 25 (17 to 37) | 6.75 (4.62 to 9.76) |  | 407 (263 to 613) | 13.91 (8.99 to 20.99) | 2.26 (1.63 to 2.9) |
| Republic of Korea | 5701 (4789 to 6752) | 18.28 (15.38 to 21.62) |  | 17472 (12893 to 23216) | 37.19 (27.36 to 49.61) | 2.22 (1.24 to 3.21) |
| Republic of Moldova | 750 (638 to 879) | 25.54 (21.8 to 29.88) |  | 759 (585 to 966) | 25.73 (19.8 to 32.77) | -0.24 (-0.8 to 0.34) |
| Romania | 2871 (2459 to 3343) | 18.33 (15.69 to 21.37) |  | 5450 (4013 to 7216) | 34.95 (25.68 to 46.37) | 1.82 (1.27 to 2.38) |
| Russian Federation | 22670 (20567 to 24333) | 22.83 (20.74 to 24.49) |  | 42513 (35948 to 49925) | 36.77 (31.08 to 43.17) | 1.3 (0.42 to 2.18) |
| Rwanda | 195 (120 to 286) | 6.11 (3.75 to 8.94) |  | 464 (286 to 725) | 6.49 (4.03 to 10.07) | 0.11 (-0.17 to 0.39) |
| Saint Kitts and Nevis | 5 (4 to 6) | 22.66 (18.04 to 28.01) |  | 10 (3 to 16) | 19.51 (6.35 to 32.09) | -0.41 (-0.61 to -0.21) |
| Saint Lucia | 8 (7 to 10) | 11.75 (9.59 to 14.17) |  | 26 (19 to 33) | 17.8 (13.37 to 23.19) | 1.34 (0.97 to 1.71) |
| Saint Vincent and the Grenadines | 7 (5 to 8) | 12.24 (9.76 to 15.14) |  | 16 (12 to 20) | 18.41 (14.14 to 23.64) | 1.32 (0.89 to 1.75) |
| Samoa | 7 (5 to 11) | 9.46 (6.23 to 13.98) |  | 15 (9 to 23) | 11.99 (6.82 to 18.48) | 0.86 (0.69 to 1.02) |
| San Marino | 5 (4 to 7) | 29.58 (21.3 to 40.57) |  | 11 (7 to 15) | 40.81 (26.77 to 59.73) | 1.17 (1.1 to 1.25) |
| Sao Tome and Principe | 3 (2 to 4) | 5.09 (2.94 to 7.52) |  | 13 (7 to 21) | 9.58 (5.51 to 15.53) | 2.42 (2.12 to 2.71) |
| Saudi Arabia | 463 (293 to 714) | 5.2 (3.31 to 7.98) |  | 6908 (4356 to 10624) | 19.2 (12.15 to 29.44) | 4.61 (4.51 to 4.72) |
| Senegal | 146 (97 to 207) | 4.17 (2.78 to 5.85) |  | 407 (247 to 614) | 4.97 (3.05 to 7.46) | 0.43 (-0.38 to 1.25) |
| Serbia | 1762 (1278 to 2381) | 26.18 (18.93 to 35.44) |  | 2816 (1961 to 3947) | 43.01 (29.93 to 60.33) | 1.59 (1.47 to 1.71) |
| Seychelles | 7 (5 to 10) | 17.83 (13.23 to 23.57) |  | 32 (23 to 43) | 37.19 (26.65 to 50.51) | 2.5 (2.33 to 2.67) |
| Sierra Leone | 58 (38 to 86) | 3.09 (2 to 4.51) |  | 187 (117 to 280) | 4.11 (2.58 to 6.14) | 0.97 (0.77 to 1.17) |
| Singapore | 799 (654 to 961) | 32.89 (26.98 to 39.51) |  | 1564 (1124 to 2100) | 29.24 (21.02 to 39.29) | -0.51 (-0.83 to -0.2) |
| Slovakia | 1243 (1025 to 1499) | 33.43 (27.61 to 40.28) |  | 2100 (1420 to 2986) | 45.66 (30.87 to 64.94) | 1.28 (0.99 to 1.57) |
| Slovenia | 446 (313 to 615) | 30.24 (21.21 to 41.68) |  | 598 (408 to 861) | 36.58 (24.96 to 52.55) | 0.6 (0.21 to 1) |
| Solomon Islands | 18 (9 to 29) | 11.28 (5.85 to 18.18) |  | 66 (35 to 103) | 16.24 (8.51 to 25.11) | 1.34 (1.21 to 1.48) |
| Somalia | 121 (73 to 197) | 3.33 (2.01 to 5.43) |  | 335 (171 to 645) | 3.28 (1.67 to 6.29) | -0.04 (-0.09 to 0.02) |
| South Africa | 2065 (1744 to 2415) | 9.64 (8.19 to 11.21) |  | 3773 (2698 to 5029) | 9.22 (6.63 to 12.26) | -0.18 (-0.4 to 0.05) |
| South Sudan | 149 (76 to 275) | 5.47 (2.8 to 10.1) |  | 274 (156 to 462) | 5.63 (3.21 to 9.46) | 0.07 (-0.1 to 0.23) |
| Spain | 9153 (7915 to 10525) | 34.71 (30.01 to 39.92) |  | 16514 (11555 to 22738) | 40.81 (28.59 to 56.19) | 0.3 (0.1 to 0.51) |
| Sri Lanka | 635 (470 to 840) | 5.59 (4.16 to 7.38) |  | 1767 (1141 to 2600) | 10.96 (7.07 to 16.14) | 2.24 (2.06 to 2.41) |
| Sudan | 404 (245 to 623) | 4.12 (2.51 to 6.33) |  | 1712 (924 to 2858) | 7.27 (3.92 to 12.11) | 1.95 (1.91 to 1.99) |
| Suriname | 25 (18 to 33) | 10.93 (7.84 to 14.47) |  | 78 (54 to 110) | 18.85 (12.95 to 26.45) | 1.91 (1.8 to 2.02) |
| Sweden | 1759 (1515 to 2034) | 25.81 (22.14 to 29.95) |  | 2283 (1786 to 2852) | 31.51 (24.64 to 39.43) | 0.83 (0.72 to 0.95) |
| Switzerland | 1413 (1179 to 1680) | 25.44 (21.17 to 30.32) |  | 1942 (1364 to 2687) | 28.58 (20.04 to 39.61) | 0.13 (-0.12 to 0.39) |
| Syrian Arab Republic | 472 (319 to 669) | 7.67 (5.24 to 10.78) |  | 1021 (681 to 1483) | 9.96 (6.64 to 14.48) | 1.15 (0.68 to 1.62) |
| Taiwan (Province of China) | 4946 (4298 to 5682) | 35.87 (31.23 to 41.13) |  | 18484 (12967 to 25849) | 91.46 (64.15 to 127.86) | 3.19 (3.03 to 3.35) |
| Tajikistan | 441 (346 to 556) | 16.87 (13.38 to 21.1) |  | 723 (496 to 1022) | 11.53 (7.93 to 16.27) | -1.52 (-1.86 to -1.18) |
| Thailand | 4779 (3612 to 6262) | 12.78 (9.7 to 16.71) |  | 14049 (9241 to 20496) | 23.67 (15.56 to 34.54) | 1.91 (1.68 to 2.13) |
| Timor-Leste | 25 (15 to 38) | 5.62 (3.39 to 8.64) |  | 80 (24 to 127) | 10.91 (3.15 to 17.25) | 2.32 (2.15 to 2.48) |
| Togo | 70 (48 to 100) | 4.13 (2.85 to 5.83) |  | 234 (140 to 363) | 4.95 (2.96 to 7.66) | 0.68 (0.59 to 0.76) |
| Tokelau | 0 (0 to 0) | 9.9 (6.06 to 15.19) |  | 0 (0 to 0) | 15.5 (8.38 to 24.64) | 1.58 (1.51 to 1.66) |
| Tonga | 3 (2 to 3) | 5.32 (3.72 to 7.27) |  | 4 (3 to 7) | 7.2 (4.64 to 10.79) | 1.02 (0.93 to 1.12) |
| Trinidad and Tobago | 115 (95 to 139) | 15.25 (12.59 to 18.35) |  | 228 (151 to 327) | 21.56 (14.24 to 30.87) | 0.82 (0.26 to 1.38) |
| Tunisia | 298 (208 to 415) | 6.68 (4.69 to 9.24) |  | 1269 (807 to 1882) | 13.85 (8.81 to 20.57) | 2.52 (2.48 to 2.56) |
| Turkey | 4770 (3129 to 6924) | 13.91 (9.15 to 20.11) |  | 14829 (10374 to 20589) | 22.44 (15.7 to 31.18) | 1.48 (1.31 to 1.65) |
| Turkmenistan | 244 (202 to 294) | 12.62 (10.52 to 15.11) |  | 412 (297 to 558) | 11.38 (8.23 to 15.42) | -0.45 (-1.14 to 0.24) |
| Tuvalu | 1 (0 to 1) | 10.15 (6.81 to 14.67) |  | 1 (1 to 2) | 14.19 (8.55 to 22.06) | 1.13 (1.04 to 1.22) |
| Uganda | 266 (167 to 388) | 3.62 (2.33 to 5.24) |  | 1649 (1057 to 2482) | 8.7 (5.61 to 13.02) | 3.11 (2.97 to 3.26) |
| Ukraine | 11144 (9664 to 12758) | 31.31 (27.14 to 35.87) |  | 11500 (8758 to 14805) | 32.84 (24.95 to 42.36) | 0.58 (-0.02 to 1.17) |
| United Arab Emirates | 125 (77 to 194) | 8.54 (5.3 to 13.26) |  | 1656 (1022 to 2588) | 12.14 (7.51 to 18.94) | 1.17 (1.08 to 1.25) |
| United Kingdom | 12647 (12056 to 13295) | 29.95 (28.55 to 31.5) |  | 19270 (15839 to 23263) | 39.37 (32.41 to 47.48) | 1.12 (1.01 to 1.24) |
| United Republic of Tanzania | 573 (382 to 847) | 4.92 (3.3 to 7.23) |  | 2049 (1269 to 3177) | 6.88 (4.28 to 10.62) | 1.19 (1.03 to 1.36) |
| United States of America | 73533 (70124 to 77065) | 39.19 (37.38 to 41.06) |  | 120799 (102690 to 142297) | 52.16 (44.37 to 61.41) | 1 (0.6 to 1.42) |
| United States Virgin Islands | 18 (12 to 25) | 21.55 (15.12 to 29.89) |  | 25 (16 to 38) | 34.33 (21.95 to 53.4) | 1.66 (1.51 to 1.81) |
| Uruguay | 472 (388 to 563) | 23.1 (19.01 to 27.6) |  | 746 (523 to 1039) | 30.55 (21.38 to 42.54) | 0.9 (0.65 to 1.14) |
| Uzbekistan | 1426 (1220 to 1660) | 13.34 (11.5 to 15.42) |  | 3206 (2482 to 4072) | 13.43 (10.4 to 17.04) | -0.09 (-0.27 to 0.09) |
| Vanuatu | 5 (3 to 9) | 6.89 (4.04 to 10.64) |  | 17 (10 to 26) | 9.69 (5.63 to 14.82) | 1.12 (0.92 to 1.32) |
| Venezuela (Bolivarian Republic of) | 1034 (881 to 1212) | 9.55 (8.18 to 11.15) |  | 4204 (2903 to 5957) | 20.05 (13.84 to 28.42) | 2.77 (2.46 to 3.09) |
| Viet Nam | 2464 (1676 to 3511) | 7.66 (5.25 to 10.86) |  | 19965 (12894 to 29715) | 25.26 (16.3 to 37.61) | 4.21 (4.13 to 4.29) |
| Yemen | 229 (121 to 392) | 4.03 (2.16 to 6.84) |  | 1028 (604 to 1622) | 5.95 (3.53 to 9.35) | 1.4 (1.25 to 1.56) |
| Zambia | 236 (148 to 348) | 6.72 (4.25 to 9.87) |  | 1002 (609 to 1527) | 10.42 (6.32 to 15.9) | 1.52 (1.45 to 1.6) |
| Zimbabwe | 281 (205 to 376) | 6.14 (4.53 to 8.14) |  | 840 (521 to 1267) | 9.89 (6.16 to 14.84) | 1.61 (1.3 to 1.92) |

UI: uncertainty interval, CI: confidence interval, AAPC, average annual percent change.
