# Supplementary material for: The global, regional, and national early-onset colorectal cancer burden and trends from 1990 to 2019: results from the Global Burden of Disease Study 2019
Source: BMC Public Health. 2022 Oct 12;22:1896. doi: 10.1186/s12889-022-14274-7 (PMC9555189; doi:10.1186/s12889-022-14274-7)
Supplement: Supplementary file 8 — Additional file 8: Table S3. Mortality of early-onset colorectal cancer in 1990 and 2019 with AAPC from 2009 and 2019 at countries/territories level, both sexes. [file 12889_2022_14274_MOESM8_ESM.docx]

Table S3. Mortality of early-onset colorectal cancer in 1990 and 2019 with AAPC from 2009 and 2019 at countries/territories level, both sexes.

| Countries/territories | 1990 | |  | 2019 | | AAPC % (95% CI)  1990-2019 |
| --- | --- | --- | --- | --- | --- | --- |
|  | Cases (95% UI) | Age-standardized mortality per  100 000 population (95% UI) |  | Cases (95% UI) | Age-standardized mortality per  100 000 population (95% UI) |  |
| Afghanistan | 83 (33 to 155) | 1.6 (0.63 to 3) |  | 305 (154 to 501) | 1.56 (0.78 to 2.57) | -0.06 (-0.12 to 0) |
| Albania | 26 (20 to 32) | 1.28 (1.02 to 1.57) |  | 24 (16 to 35) | 1.34 (0.9 to 1.93) | 0.04 (-0.32 to 0.4) |
| Algeria | 122 (85 to 172) | 1.06 (0.74 to 1.48) |  | 289 (191 to 417) | 0.91 (0.6 to 1.32) | -0.53 (-0.61 to -0.46) |
| American Samoa | 1 (0 to 1) | 2.11 (1.43 to 3) |  | 1 (1 to 1) | 2.31 (1.51 to 3.46) | 0.45 (0.11 to 0.78) |
| Andorra | 1 (1 to 1) | 2.16 (1.34 to 3.24) |  | 1 (1 to 2) | 1.68 (1.07 to 2.48) | -0.92 (-1.04 to -0.8) |
| Angola | 54 (32 to 90) | 1.09 (0.64 to 1.81) |  | 177 (107 to 266) | 1.21 (0.74 to 1.81) | 0.31 (0.04 to 0.59) |
| Antigua and Barbuda | 0 (0 to 1) | 1.24 (0.97 to 1.56) |  | 1 (1 to 1) | 1.2 (0.88 to 1.61) | -0.08 (-0.41 to 0.24) |
| Argentina | 436 (386 to 492) | 2.03 (1.8 to 2.29) |  | 785 (651 to 942) | 2.38 (1.97 to 2.85) | 0.48 (0.33 to 0.62) |
| Armenia | 48 (40 to 57) | 2.47 (2.09 to 2.88) |  | 34 (26 to 44) | 1.58 (1.21 to 2.01) | -1.63 (-2.63 to -0.61) |
| Australia | 258 (234 to 283) | 2.04 (1.86 to 2.25) |  | 283 (234 to 340) | 1.53 (1.26 to 1.84) | -0.87 (-1.04 to -0.7) |
| Austria | 145 (129 to 164) | 2.45 (2.16 to 2.76) |  | 62 (50 to 76) | 0.89 (0.71 to 1.08) | -3.66 (-3.97 to -3.36) |
| Azerbaijan | 114 (93 to 140) | 2.91 (2.39 to 3.55) |  | 163 (113 to 229) | 2.07 (1.44 to 2.91) | -0.93 (-1.52 to -0.34) |
| Bahamas | 4 (3 to 5) | 2.5 (2.05 to 3.03) |  | 8 (6 to 11) | 2.69 (1.98 to 3.64) | 0.14 (-0.35 to 0.64) |
| Bahrain | 4 (3 to 5) | 1.14 (0.81 to 1.55) |  | 16 (11 to 23) | 0.97 (0.65 to 1.4) | -0.48 (-0.76 to -0.21) |
| Bangladesh | 334 (207 to 496) | 0.64 (0.4 to 0.95) |  | 616 (364 to 992) | 0.56 (0.33 to 0.9) | -0.44 (-0.69 to -0.19) |
| Barbados | 3 (3 to 4) | 2.12 (1.77 to 2.52) |  | 5 (4 to 7) | 2.25 (1.67 to 2.98) | 0.44 (0.06 to 0.82) |
| Belarus | 179 (156 to 204) | 2.66 (2.32 to 3.02) |  | 118 (81 to 167) | 1.6 (1.09 to 2.25) | -2.01 (-2.76 to -1.26) |
| Belgium | 127 (111 to 146) | 1.8 (1.57 to 2.06) |  | 93 (75 to 114) | 1.09 (0.88 to 1.34) | -1.83 (-2.18 to -1.48) |
| Belize | 1 (0 to 1) | 0.7 (0.56 to 0.85) |  | 4 (3 to 5) | 1.44 (1.12 to 1.82) | 2.3 (1.86 to 2.73) |
| Benin | 15 (10 to 22) | 0.73 (0.5 to 1.03) |  | 50 (31 to 77) | 0.81 (0.5 to 1.24) | 0.43 (0.34 to 0.51) |
| Bermuda | 1 (1 to 1) | 2.18 (1.69 to 2.74) |  | 1 (1 to 1) | 1.54 (1.09 to 2.12) | -1.35 (-1.71 to -1) |
| Bhutan | 2 (1 to 3) | 0.68 (0.34 to 1.15) |  | 4 (2 to 6) | 0.73 (0.34 to 1.21) | 0.19 (0.06 to 0.32) |
| Bolivia (Plurinational State of) | 36 (23 to 51) | 1.07 (0.69 to 1.51) |  | 90 (53 to 140) | 1.17 (0.69 to 1.82) | 0.33 (0.23 to 0.44) |
| Bosnia and Herzegovina | 59 (49 to 71) | 1.91 (1.59 to 2.29) |  | 58 (41 to 79) | 2.28 (1.63 to 3.12) | 0.39 (-0.02 to 0.79) |
| Botswana | 8 (4 to 12) | 1.27 (0.76 to 2.01) |  | 32 (18 to 53) | 1.93 (1.07 to 3.14) | 1.38 (1.13 to 1.63) |
| Brazil | 1237 (1167 to 1313) | 1.39 (1.31 to 1.47) |  | 2795 (2593 to 3011) | 1.65 (1.53 to 1.78) | 0.66 (0.51 to 0.8) |
| Brunei Darussalam | 5 (3 to 7) | 3.06 (2.16 to 4.17) |  | 11 (8 to 15) | 2.94 (2.13 to 3.95) | -0.16 (-0.44 to 0.13) |
| Bulgaria | 189 (164 to 215) | 2.91 (2.52 to 3.32) |  | 156 (109 to 214) | 2.73 (1.91 to 3.77) | -0.14 (-0.6 to 0.32) |
| Burkina Faso | 27 (17 to 40) | 0.64 (0.42 to 0.95) |  | 90 (57 to 133) | 0.81 (0.52 to 1.2) | 0.95 (0.83 to 1.07) |
| Burundi | 30 (19 to 45) | 1.24 (0.77 to 1.86) |  | 57 (35 to 90) | 1.01 (0.61 to 1.59) | -0.79 (-1 to -0.57) |
| Cabo Verde | 1 (1 to 1) | 0.76 (0.55 to 1.03) |  | 4 (2 to 6) | 0.98 (0.64 to 1.46) | 0.82 (0.61 to 1.03) |
| Cambodia | 75 (46 to 111) | 1.54 (0.96 to 2.27) |  | 215 (144 to 318) | 2 (1.34 to 2.95) | 0.88 (0.74 to 1.01) |
| Cameroon | 51 (35 to 72) | 1.08 (0.73 to 1.5) |  | 184 (109 to 290) | 1.2 (0.72 to 1.89) | 0.35 (0.25 to 0.45) |
| Canada | 343 (313 to 377) | 1.64 (1.49 to 1.8) |  | 364 (293 to 446) | 1.37 (1.11 to 1.69) | -0.59 (-0.82 to -0.37) |
| Central African Republic | 14 (9 to 21) | 1.09 (0.66 to 1.62) |  | 27 (16 to 44) | 0.98 (0.57 to 1.56) | -0.45 (-0.76 to -0.14) |
| Chad | 16 (11 to 23) | 0.61 (0.4 to 0.88) |  | 56 (35 to 83) | 0.8 (0.51 to 1.18) | 0.98 (0.86 to 1.1) |
| Chile | 101 (86 to 119) | 1.18 (1 to 1.38) |  | 185 (149 to 226) | 1.34 (1.08 to 1.64) | 0.74 (0.25 to 1.23) |
| China | 14974 (12737 to 17466) | 1.91 (1.63 to 2.23) |  | 26320 (21827 to 31571) | 2.17 (1.8 to 2.59) | 0.29 (0.04 to 0.54) |
| Colombia | 227 (197 to 259) | 1.18 (1.03 to 1.34) |  | 493 (340 to 694) | 1.45 (1 to 2.03) | 0.85 (0.69 to 1.01) |
| Comoros | 2 (1 to 4) | 1.01 (0.28 to 1.73) |  | 5 (3 to 8) | 1.14 (0.62 to 1.79) | -0.07 (-1.89 to 1.79) |
| Congo | 19 (10 to 30) | 1.74 (0.93 to 2.83) |  | 49 (28 to 76) | 1.43 (0.82 to 2.24) | -0.5 (-0.79 to -0.21) |
| Cook Islands | 0 (0 to 0) | 1.11 (0.71 to 1.65) |  | 0 (0 to 0) | 1.01 (0.55 to 1.55) | -0.26 (-0.38 to -0.15) |
| Costa Rica | 20 (17 to 24) | 1.17 (0.97 to 1.38) |  | 69 (49 to 96) | 1.99 (1.41 to 2.76) | 2.04 (1.83 to 2.26) |
| Croatia | 81 (68 to 94) | 2.21 (1.87 to 2.6) |  | 60 (42 to 83) | 1.87 (1.29 to 2.59) | -0.49 (-0.81 to -0.17) |
| Cuba | 125 (108 to 144) | 1.65 (1.43 to 1.91) |  | 140 (103 to 186) | 1.5 (1.1 to 2) | -0.28 (-0.5 to -0.06) |
| Cyprus | 5 (4 to 7) | 0.89 (0.65 to 1.19) |  | 10 (7 to 13) | 0.91 (0.67 to 1.2) | -0.15 (-0.53 to 0.23) |
| Czechia | 251 (227 to 279) | 3.04 (2.74 to 3.37) |  | 138 (101 to 183) | 1.46 (1.07 to 1.94) | -2.73 (-2.97 to -2.5) |
| C么te d'Ivoire | 59 (39 to 87) | 1.04 (0.68 to 1.53) |  | 151 (92 to 229) | 1.01 (0.62 to 1.53) | -0.1 (-0.28 to 0.07) |
| Democratic People's Republic of Korea | 329 (198 to 504) | 2.33 (1.4 to 3.56) |  | 473 (270 to 781) | 2.18 (1.24 to 3.61) | -0.27 (-0.32 to -0.22) |
| Democratic Republic of the Congo | 140 (90 to 210) | 0.83 (0.53 to 1.23) |  | 331 (193 to 534) | 0.76 (0.44 to 1.21) | -0.34 (-0.51 to -0.16) |
| Denmark | 82 (73 to 93) | 1.96 (1.72 to 2.23) |  | 54 (44 to 66) | 1.28 (1.03 to 1.56) | -1.48 (-2.01 to -0.95) |
| Djibouti | 3 (1 to 4) | 1.14 (0.67 to 1.77) |  | 12 (6 to 19) | 1.41 (0.77 to 2.32) | 0.78 (0.68 to 0.87) |
| Dominica | 1 (0 to 1) | 1.4 (1.02 to 1.85) |  | 1 (1 to 1) | 1.63 (1.1 to 2.34) | 0.55 (0.26 to 0.84) |
| Dominican Republic | 42 (32 to 55) | 1.09 (0.82 to 1.42) |  | 140 (88 to 210) | 1.85 (1.16 to 2.77) | 2.18 (1.88 to 2.48) |
| Ecuador | 50 (41 to 60) | 0.9 (0.74 to 1.08) |  | 171 (119 to 241) | 1.44 (0.99 to 2.02) | 1.41 (1.07 to 1.75) |
| Egypt | 334 (253 to 436) | 1.05 (0.81 to 1.36) |  | 777 (482 to 1163) | 1.2 (0.75 to 1.8) | 0.47 (0.26 to 0.69) |
| El Salvador | 24 (19 to 29) | 0.88 (0.71 to 1.07) |  | 66 (44 to 95) | 1.58 (1.05 to 2.28) | 2.1 (1.71 to 2.49) |
| Equatorial Guinea | 2 (1 to 3) | 0.92 (0.53 to 1.47) |  | 11 (5 to 19) | 1.54 (0.78 to 2.72) | 1.88 (1.65 to 2.12) |
| Eritrea | 15 (9 to 22) | 1.08 (0.65 to 1.62) |  | 56 (35 to 87) | 1.54 (0.95 to 2.37) | 1.22 (0.91 to 1.53) |
| Estonia | 23 (19 to 27) | 2.08 (1.74 to 2.47) |  | 15 (10 to 20) | 1.44 (0.99 to 2.04) | -1.35 (-2.47 to -0.23) |
| Eswatini | 4 (3 to 6) | 1.13 (0.73 to 1.69) |  | 12 (6 to 21) | 1.86 (0.99 to 3.15) | 1.64 (1.41 to 1.86) |
| Ethiopia | 307 (185 to 469) | 1.35 (0.82 to 2.07) |  | 531 (374 to 758) | 1.01 (0.71 to 1.45) | -1.07 (-1.28 to -0.86) |
| Fiji | 6 (4 to 9) | 1.38 (0.95 to 1.95) |  | 10 (6 to 14) | 1.54 (1.02 to 2.24) | 0.43 (0.16 to 0.7) |
| Finland | 54 (47 to 63) | 1.3 (1.13 to 1.51) |  | 35 (27 to 43) | 0.93 (0.73 to 1.15) | -1.36 (-1.68 to -1.04) |
| France | 698 (629 to 773) | 1.72 (1.55 to 1.9) |  | 586 (474 to 714) | 1.22 (0.98 to 1.49) | -1.31 (-1.65 to -0.96) |
| Gabon | 9 (5 to 15) | 1.9 (1.02 to 3.27) |  | 19 (11 to 30) | 1.7 (0.99 to 2.71) | -0.41 (-0.62 to -0.21) |
| Gambia | 2 (1 to 3) | 0.52 (0.32 to 0.79) |  | 8 (5 to 12) | 0.69 (0.41 to 1.08) | 1.14 (0.42 to 1.88) |
| Georgia | 87 (70 to 105) | 2.09 (1.69 to 2.54) |  | 59 (42 to 79) | 1.49 (1.08 to 2) | -1.64 (-2.28 to -0.99) |
| Germany | 1149 (1043 to 1260) | 1.92 (1.74 to 2.11) |  | 793 (649 to 950) | 1.33 (1.08 to 1.6) | -1.88 (-2.55 to -1.21) |
| Ghana | 66 (43 to 94) | 0.87 (0.58 to 1.24) |  | 222 (139 to 335) | 1.13 (0.71 to 1.7) | 0.88 (0.67 to 1.08) |
| Greece | 77 (67 to 88) | 1.05 (0.91 to 1.19) |  | 94 (79 to 110) | 1.1 (0.93 to 1.3) | 0.03 (-0.16 to 0.22) |
| Greenland | 2 (1 to 2) | 3.57 (2.53 to 4.86) |  | 1 (1 to 2) | 3.19 (2.21 to 4.57) | -0.44 (-0.71 to -0.16) |
| Grenada | 1 (1 to 1) | 1.79 (1.37 to 2.31) |  | 1 (1 to 2) | 1.94 (1.41 to 2.57) | 0.24 (0.05 to 0.44) |
| Guam | 2 (1 to 2) | 1.9 (1.33 to 2.67) |  | 3 (2 to 4) | 2.5 (1.7 to 3.55) | 1.03 (0.93 to 1.13) |
| Guatemala | 31 (25 to 38) | 0.79 (0.64 to 0.97) |  | 161 (117 to 217) | 1.48 (1.07 to 1.98) | 2.25 (1.86 to 2.66) |
| Guinea | 19 (13 to 27) | 0.65 (0.45 to 0.9) |  | 50 (31 to 75) | 0.82 (0.52 to 1.24) | 0.82 (0.66 to 0.98) |
| Guinea-Bissau | 5 (3 to 8) | 1.2 (0.73 to 1.83) |  | 12 (7 to 17) | 1.2 (0.76 to 1.78) | -0.01 (-0.09 to 0.07) |
| Guyana | 7 (5 to 9) | 1.64 (1.22 to 2.13) |  | 12 (8 to 18) | 2.33 (1.55 to 3.36) | 1.15 (0.67 to 1.64) |
| Haiti | 50 (29 to 75) | 1.51 (0.88 to 2.27) |  | 107 (63 to 168) | 1.37 (0.8 to 2.14) | -0.32 (-0.47 to -0.16) |
| Honduras | 20 (14 to 27) | 0.89 (0.64 to 1.22) |  | 50 (28 to 84) | 0.86 (0.48 to 1.43) | -0.09 (-0.37 to 0.2) |
| Hungary | 242 (214 to 274) | 3.02 (2.66 to 3.43) |  | 187 (140 to 248) | 2.25 (1.68 to 2.98) | -1.22 (-2 to -0.42) |
| Iceland | 2 (2 to 2) | 1.16 (0.94 to 1.41) |  | 2 (2 to 3) | 0.86 (0.66 to 1.1) | -1.04 (-1.3 to -0.79) |
| India | 4107 (3508 to 4797) | 0.83 (0.71 to 0.96) |  | 10062 (8250 to 12148) | 1.03 (0.84 to 1.24) | 0.74 (0.38 to 1.09) |
| Indonesia | 1641 (1174 to 2063) | 1.52 (1.09 to 1.91) |  | 4216 (2962 to 5624) | 2.03 (1.43 to 2.7) | 1 (0.93 to 1.07) |
| Iran (Islamic Republic of) | 286 (232 to 353) | 1.06 (0.87 to 1.31) |  | 837 (760 to 925) | 1.19 (1.08 to 1.31) | 0.37 (0.25 to 0.5) |
| Iraq | 92 (56 to 144) | 1.13 (0.7 to 1.77) |  | 353 (231 to 528) | 1.29 (0.84 to 1.93) | 0.6 (0.39 to 0.81) |
| Ireland | 48 (41 to 56) | 2.02 (1.72 to 2.36) |  | 46 (36 to 57) | 1.13 (0.89 to 1.43) | -1.88 (-2.64 to -1.12) |
| Israel | 51 (43 to 61) | 1.7 (1.44 to 2.02) |  | 84 (68 to 103) | 1.31 (1.05 to 1.61) | -1 (-1.35 to -0.66) |
| Italy | 768 (733 to 801) | 1.84 (1.76 to 1.92) |  | 596 (551 to 640) | 1.19 (1.1 to 1.28) | -1.49 (-1.73 to -1.25) |
| Jamaica | 11 (8 to 13) | 0.88 (0.69 to 1.09) |  | 41 (28 to 58) | 2.05 (1.39 to 2.88) | 3.84 (2.54 to 5.15) |
| Japan | 2253 (2191 to 2315) | 2.1 (2.04 to 2.16) |  | 1429 (1340 to 1498) | 1.42 (1.33 to 1.49) | -1.45 (-1.75 to -1.14) |
| Jordan | 27 (19 to 38) | 1.7 (1.2 to 2.34) |  | 114 (83 to 155) | 1.43 (1.04 to 1.94) | -0.52 (-0.67 to -0.36) |
| Kazakhstan | 226 (197 to 255) | 2.34 (2.06 to 2.64) |  | 197 (155 to 247) | 1.44 (1.13 to 1.81) | -1.6 (-2.61 to -0.57) |
| Kenya | 61 (41 to 78) | 0.64 (0.43 to 0.82) |  | 281 (209 to 366) | 1.01 (0.76 to 1.32) | 1.54 (1.34 to 1.74) |
| Kiribati | 1 (1 to 1) | 2.3 (1.55 to 3.31) |  | 2 (1 to 2) | 2.11 (1.33 to 3.28) | -0.33 (-0.39 to -0.26) |
| Kuwait | 9 (7 to 11) | 0.76 (0.59 to 0.95) |  | 33 (24 to 45) | 0.71 (0.51 to 0.96) | 0.08 (-0.96 to 1.14) |
| Kyrgyzstan | 49 (40 to 60) | 2.19 (1.78 to 2.65) |  | 43 (32 to 55) | 0.98 (0.74 to 1.27) | -2.93 (-3.5 to -2.37) |
| Lao People's Democratic Republic | 40 (21 to 62) | 1.94 (1.06 to 3.03) |  | 98 (57 to 148) | 2.06 (1.21 to 3.14) | 0.22 (0.17 to 0.27) |
| Latvia | 42 (36 to 48) | 2.15 (1.84 to 2.5) |  | 20 (15 to 28) | 1.43 (1.03 to 1.95) | -1.21 (-3.15 to 0.77) |
| Lebanon | 30 (19 to 44) | 1.66 (1.08 to 2.41) |  | 68 (45 to 97) | 1.8 (1.2 to 2.57) | 0.33 (0.24 to 0.42) |
| Lesotho | 6 (4 to 10) | 0.67 (0.41 to 1.11) |  | 20 (11 to 31) | 1.56 (0.89 to 2.44) | 3.2 (2.78 to 3.62) |
| Liberia | 7 (5 to 11) | 0.75 (0.49 to 1.08) |  | 21 (12 to 34) | 0.71 (0.4 to 1.14) | -0.3 (-0.56 to -0.04) |
| Libya | 35 (22 to 56) | 1.82 (1.12 to 2.87) |  | 115 (74 to 172) | 1.87 (1.21 to 2.79) | -0.03 (-0.36 to 0.31) |
| Lithuania | 55 (47 to 63) | 2.12 (1.83 to 2.45) |  | 31 (22 to 41) | 1.48 (1.08 to 2.01) | -0.92 (-3.09 to 1.3) |
| Luxembourg | 6 (5 to 7) | 2.07 (1.75 to 2.43) |  | 5 (4 to 7) | 1.04 (0.83 to 1.29) | -2.38 (-2.57 to -2.18) |
| Madagascar | 64 (41 to 95) | 1.19 (0.76 to 1.74) |  | 153 (96 to 231) | 1.06 (0.66 to 1.6) | -0.43 (-0.86 to 0.01) |
| Malawi | 26 (18 to 37) | 0.61 (0.41 to 0.85) |  | 65 (39 to 102) | 0.71 (0.43 to 1.12) | 0.49 (0.24 to 0.75) |
| Malaysia | 179 (133 to 234) | 1.75 (1.32 to 2.29) |  | 444 (296 to 639) | 1.96 (1.31 to 2.83) | 0.36 (-0.09 to 0.82) |
| Maldives | 1 (1 to 2) | 1.17 (0.62 to 1.81) |  | 3 (2 to 4) | 0.73 (0.5 to 1.03) | -1.76 (-1.98 to -1.54) |
| Mali | 34 (24 to 47) | 0.85 (0.6 to 1.15) |  | 90 (55 to 136) | 0.92 (0.57 to 1.39) | 0.25 (0.05 to 0.45) |
| Malta | 3 (3 to 4) | 1.09 (0.88 to 1.35) |  | 3 (3 to 4) | 1.05 (0.79 to 1.35) | -0.06 (-0.36 to 0.24) |
| Marshall Islands | 0 (0 to 1) | 1.77 (1.21 to 2.47) |  | 1 (1 to 1) | 2.2 (1.33 to 3.38) | 0.76 (0.69 to 0.83) |
| Mauritania | 9 (6 to 13) | 0.94 (0.62 to 1.35) |  | 15 (9 to 24) | 0.73 (0.42 to 1.14) | -0.86 (-1.04 to -0.69) |
| Mauritius | 8 (6 to 9) | 1.09 (0.91 to 1.3) |  | 16 (12 to 22) | 1.62 (1.19 to 2.17) | 1.34 (0.61 to 2.08) |
| Mexico | 420 (401 to 439) | 0.91 (0.87 to 0.95) |  | 1341 (1126 to 1572) | 1.45 (1.22 to 1.7) | 1.95 (1.65 to 2.25) |
| Micronesia (Federated States of) | 1 (1 to 2) | 2.15 (1.31 to 3.2) |  | 1 (0 to 2) | 2.19 (0.62 to 3.65) | 0.02 (-0.06 to 0.1) |
| Monaco | 0 (0 to 1) | 1.95 (1.34 to 2.72) |  | 1 (0 to 1) | 1.94 (1.27 to 2.85) | -0.02 (-0.04 to 0.01) |
| Mongolia | 14 (10 to 19) | 1.36 (0.95 to 1.91) |  | 38 (25 to 56) | 1.41 (0.93 to 2.08) | 0.02 (-0.16 to 0.21) |
| Montenegro | 7 (5 to 9) | 1.66 (1.25 to 2.14) |  | 7 (5 to 9) | 1.47 (1.09 to 1.93) | -0.61 (-1.2 to -0.02) |
| Morocco | 111 (77 to 153) | 0.83 (0.58 to 1.15) |  | 254 (159 to 401) | 0.96 (0.6 to 1.51) | 0.44 (0.32 to 0.57) |
| Mozambique | 35 (23 to 51) | 0.56 (0.37 to 0.8) |  | 134 (78 to 209) | 1 (0.58 to 1.56) | 2.17 (1.93 to 2.41) |
| Myanmar | 391 (240 to 591) | 1.76 (1.09 to 2.67) |  | 794 (497 to 1198) | 2 (1.25 to 3.02) | 0.53 (0.33 to 0.73) |
| Namibia | 5 (3 to 7) | 0.72 (0.43 to 1.08) |  | 14 (8 to 22) | 0.95 (0.55 to 1.54) | 1.23 (0.84 to 1.63) |
| Nauru | 0 (0 to 0) | 3.33 (1.72 to 5.54) |  | 0 (0 to 0) | 3.06 (1.66 to 4.7) | -0.28 (-0.34 to -0.22) |
| Nepal | 61 (36 to 96) | 0.6 (0.36 to 0.95) |  | 119 (69 to 189) | 0.63 (0.37 to 0.99) | 0.21 (0.04 to 0.39) |
| Netherlands | 213 (191 to 236) | 1.83 (1.64 to 2.03) |  | 182 (148 to 219) | 1.42 (1.15 to 1.72) | -0.99 (-1.25 to -0.72) |
| New Zealand | 64 (56 to 74) | 2.64 (2.29 to 3.05) |  | 55 (46 to 66) | 1.71 (1.42 to 2.06) | -1.52 (-1.81 to -1.22) |
| Nicaragua | 16 (12 to 22) | 0.89 (0.66 to 1.19) |  | 45 (31 to 62) | 1.03 (0.72 to 1.43) | 0.32 (-0.15 to 0.8) |
| Niger | 23 (15 to 35) | 0.64 (0.41 to 0.96) |  | 55 (33 to 87) | 0.6 (0.37 to 0.94) | -0.14 (-0.28 to 0) |
| Nigeria | 264 (178 to 386) | 0.6 (0.4 to 0.87) |  | 768 (529 to 1092) | 0.71 (0.49 to 1.01) | 0.58 (0.33 to 0.83) |
| Niue | 0 (0 to 0) | 1.97 (1.23 to 2.96) |  | 0 (0 to 0) | 1.86 (0.96 to 3.13) | -0.25 (-0.51 to 0.01) |
| North Macedonia | 27 (22 to 33) | 1.92 (1.56 to 2.34) |  | 37 (26 to 51) | 2.09 (1.49 to 2.86) | 0.24 (-0.12 to 0.61) |
| Northern Mariana Islands | 1 (1 to 2) | 2.74 (1.67 to 4.22) |  | 1 (0 to 1) | 2.38 (1.51 to 3.62) | -0.42 (-0.73 to -0.11) |
| Norway | 61 (57 to 64) | 1.92 (1.81 to 2.03) |  | 54 (48 to 59) | 1.3 (1.17 to 1.42) | -1.38 (-1.63 to -1.12) |
| Oman | 8 (5 to 13) | 0.76 (0.45 to 1.2) |  | 25 (16 to 40) | 0.65 (0.42 to 1.04) | -0.66 (-0.92 to -0.39) |
| Pakistan | 458 (338 to 603) | 0.85 (0.63 to 1.11) |  | 1757 (1240 to 2443) | 1.39 (0.98 to 1.93) | 1.72 (1.52 to 1.92) |
| Palau | 0 (0 to 0) | 1.66 (1.04 to 2.48) |  | 0 (0 to 0) | 1.56 (1 to 2.32) | -0.3 (-0.49 to -0.11) |
| Palestine | 17 (11 to 27) | 2.14 (1.3 to 3.29) |  | 58 (43 to 76) | 2.05 (1.52 to 2.71) | 0 (-0.24 to 0.24) |
| Panama | 14 (12 to 18) | 1.03 (0.83 to 1.27) |  | 39 (26 to 55) | 1.34 (0.9 to 1.89) | 0.92 (0.53 to 1.32) |
| Papua New Guinea | 23 (14 to 35) | 1.04 (0.64 to 1.58) |  | 74 (46 to 114) | 1.19 (0.74 to 1.84) | 0.44 (0.4 to 0.48) |
| Paraguay | 18 (14 to 23) | 0.84 (0.64 to 1.08) |  | 73 (48 to 106) | 1.57 (1.04 to 2.3) | 2.39 (1.84 to 2.94) |
| Peru | 122 (92 to 158) | 1.02 (0.78 to 1.31) |  | 269 (173 to 395) | 1.1 (0.71 to 1.61) | 0.25 (-0.23 to 0.72) |
| Philippines | 891 (777 to 996) | 2.56 (2.24 to 2.86) |  | 2037 (1642 to 2535) | 2.73 (2.2 to 3.4) | 0.26 (0.13 to 0.4) |
| Poland | 611 (584 to 640) | 2.32 (2.22 to 2.43) |  | 569 (463 to 685) | 1.82 (1.48 to 2.19) | -1.23 (-1.9 to -0.56) |
| Portugal | 153 (133 to 176) | 2.21 (1.91 to 2.54) |  | 157 (127 to 191) | 1.78 (1.44 to 2.18) | -0.83 (-1.49 to -0.17) |
| Puerto Rico | 42 (34 to 50) | 1.68 (1.39 to 2) |  | 49 (34 to 71) | 1.93 (1.33 to 2.76) | 0.43 (0.02 to 0.85) |
| Qatar | 3 (2 to 4) | 0.79 (0.52 to 1.18) |  | 20 (13 to 30) | 0.7 (0.46 to 1.04) | -0.25 (-1.29 to 0.8) |
| Republic of Korea | 535 (459 to 618) | 1.73 (1.49 to 1.99) |  | 569 (452 to 707) | 1.2 (0.95 to 1.49) | -1.61 (-2.54 to -0.66) |
| Republic of Moldova | 87 (76 to 100) | 2.98 (2.61 to 3.41) |  | 60 (46 to 75) | 2 (1.56 to 2.51) | -1.51 (-2.57 to -0.44) |
| Romania | 327 (287 to 373) | 2.09 (1.83 to 2.38) |  | 361 (270 to 472) | 2.26 (1.69 to 2.96) | 0.18 (-0.81 to 1.19) |
| Russian Federation | 1874 (1698 to 2001) | 1.91 (1.73 to 2.04) |  | 2244 (1882 to 2638) | 1.93 (1.62 to 2.26) | -0.31 (-0.69 to 0.07) |
| Rwanda | 43 (26 to 64) | 1.42 (0.85 to 2.11) |  | 80 (49 to 126) | 1.15 (0.7 to 1.81) | -0.79 (-1.08 to -0.5) |
| Saint Kitts and Nevis | 0 (0 to 1) | 2.39 (1.93 to 2.89) |  | 1 (0 to 1) | 1.38 (0.43 to 2.25) | -1.8 (-2.06 to -1.54) |
| Saint Lucia | 1 (1 to 1) | 1.48 (1.23 to 1.78) |  | 2 (2 to 3) | 1.5 (1.14 to 1.94) | -0.04 (-0.43 to 0.35) |
| Saint Vincent and the Grenadines | 1 (1 to 1) | 1.52 (1.22 to 1.86) |  | 2 (1 to 2) | 1.86 (1.45 to 2.37) | 0.65 (0.04 to 1.27) |
| Samoa | 1 (1 to 2) | 1.36 (0.87 to 2.04) |  | 2 (1 to 3) | 1.37 (0.77 to 2.12) | 0.02 (-0.08 to 0.12) |
| San Marino | 0 (0 to 0) | 1.46 (1.02 to 2.03) |  | 0 (0 to 1) | 1.41 (0.78 to 2.37) | -0.13 (-0.2 to -0.06) |
| Sao Tome and Principe | 1 (0 to 1) | 0.99 (0.57 to 1.46) |  | 2 (1 to 3) | 1.42 (0.81 to 2.3) | 1.44 (1.03 to 1.86) |
| Saudi Arabia | 77 (46 to 122) | 0.89 (0.53 to 1.4) |  | 496 (322 to 742) | 1.39 (0.9 to 2.07) | 1.54 (1.46 to 1.62) |
| Senegal | 29 (19 to 42) | 0.87 (0.58 to 1.23) |  | 71 (43 to 108) | 0.9 (0.55 to 1.36) | 0 (-0.45 to 0.45) |
| Serbia | 177 (130 to 236) | 2.61 (1.92 to 3.5) |  | 153 (108 to 213) | 2.32 (1.64 to 3.23) | -0.51 (-0.85 to -0.18) |
| Seychelles | 1 (1 to 1) | 2.38 (1.76 to 3.13) |  | 3 (2 to 4) | 3.22 (2.32 to 4.36) | 1.09 (0.88 to 1.31) |
| Sierra Leone | 12 (8 to 18) | 0.66 (0.42 to 0.98) |  | 34 (21 to 51) | 0.77 (0.48 to 1.16) | 0.61 (0.37 to 0.86) |
| Singapore | 54 (45 to 64) | 2.24 (1.88 to 2.64) |  | 51 (41 to 62) | 0.94 (0.75 to 1.16) | -3.23 (-3.56 to -2.9) |
| Slovakia | 99 (83 to 116) | 2.67 (2.26 to 3.14) |  | 95 (65 to 132) | 2.03 (1.39 to 2.85) | -1 (-1.19 to -0.81) |
| Slovenia | 30 (21 to 41) | 2.03 (1.44 to 2.79) |  | 22 (15 to 31) | 1.3 (0.89 to 1.85) | -1.91 (-2.22 to -1.6) |
| Solomon Islands | 3 (2 to 5) | 2.04 (1.01 to 3.34) |  | 10 (5 to 15) | 2.41 (1.23 to 3.74) | 0.63 (0.55 to 0.71) |
| Somalia | 28 (17 to 46) | 0.79 (0.47 to 1.3) |  | 76 (41 to 148) | 0.77 (0.42 to 1.49) | -0.11 (-0.2 to -0.03) |
| South Africa | 342 (289 to 401) | 1.64 (1.39 to 1.91) |  | 513 (366 to 693) | 1.26 (0.9 to 1.7) | -0.97 (-1.19 to -0.75) |
| South Sudan | 32 (16 to 61) | 1.23 (0.61 to 2.32) |  | 57 (31 to 99) | 1.2 (0.65 to 2.07) | -0.22 (-0.54 to 0.09) |
| Spain | 500 (450 to 555) | 1.9 (1.71 to 2.1) |  | 537 (441 to 649) | 1.31 (1.07 to 1.59) | -1.35 (-1.66 to -1.04) |
| Sri Lanka | 78 (58 to 104) | 0.7 (0.52 to 0.93) |  | 118 (77 to 174) | 0.73 (0.47 to 1.08) | 0.31 (-0.21 to 0.83) |
| Sudan | 79 (47 to 123) | 0.84 (0.5 to 1.29) |  | 233 (125 to 385) | 1.01 (0.55 to 1.67) | 0.63 (0.57 to 0.7) |
| Suriname | 4 (3 to 5) | 1.6 (1.15 to 2.08) |  | 9 (6 to 12) | 2.1 (1.46 to 2.92) | 0.83 (-0.22 to 1.89) |
| Sweden | 101 (88 to 114) | 1.46 (1.28 to 1.66) |  | 90 (76 to 105) | 1.23 (1.04 to 1.44) | -0.44 (-0.59 to -0.29) |
| Switzerland | 69 (59 to 79) | 1.23 (1.05 to 1.42) |  | 61 (49 to 75) | 0.88 (0.7 to 1.1) | -1.3 (-1.81 to -0.79) |
| Syrian Arab Republic | 66 (44 to 94) | 1.13 (0.76 to 1.6) |  | 90 (60 to 131) | 0.87 (0.58 to 1.28) | -0.75 (-1.16 to -0.34) |
| Taiwan (Province of China) | 355 (316 to 397) | 2.59 (2.31 to 2.88) |  | 701 (503 to 952) | 3.46 (2.48 to 4.7) | 0.86 (0.31 to 1.41) |
| Tajikistan | 64 (51 to 80) | 2.61 (2.09 to 3.24) |  | 93 (64 to 133) | 1.51 (1.04 to 2.15) | -1.94 (-2.45 to -1.43) |
| Thailand | 626 (472 to 819) | 1.7 (1.29 to 2.22) |  | 968 (644 to 1405) | 1.61 (1.07 to 2.34) | -0.41 (-0.94 to 0.13) |
| Timor-Leste | 5 (3 to 8) | 1.13 (0.66 to 1.78) |  | 11 (3 to 18) | 1.58 (0.41 to 2.53) | 1.23 (0.92 to 1.53) |
| Togo | 13 (9 to 19) | 0.83 (0.57 to 1.18) |  | 41 (24 to 64) | 0.88 (0.52 to 1.37) | 0.23 (0.06 to 0.4) |
| Tokelau | 0 (0 to 0) | 1.54 (0.93 to 2.37) |  | 0 (0 to 0) | 1.62 (0.86 to 2.59) | 0.2 (0.14 to 0.26) |
| Tonga | 0 (0 to 0) | 0.76 (0.52 to 1.05) |  | 1 (0 to 1) | 0.84 (0.53 to 1.26) | 0.34 (0.24 to 0.45) |
| Trinidad and Tobago | 14 (11 to 16) | 1.82 (1.53 to 2.14) |  | 19 (13 to 28) | 1.82 (1.22 to 2.59) | -0.35 (-0.9 to 0.22) |
| Tunisia | 39 (27 to 54) | 0.89 (0.61 to 1.24) |  | 97 (61 to 143) | 1.05 (0.66 to 1.56) | 0.55 (0.43 to 0.66) |
| Turkey | 750 (496 to 1079) | 2.24 (1.49 to 3.21) |  | 1028 (742 to 1389) | 1.55 (1.12 to 2.1) | -1.35 (-1.7 to -1) |
| Turkmenistan | 34 (28 to 40) | 1.83 (1.54 to 2.16) |  | 43 (31 to 58) | 1.19 (0.86 to 1.61) | -1.33 (-2.4 to -0.25) |
| Tuvalu | 0 (0 to 0) | 1.73 (1.14 to 2.51) |  | 0 (0 to 0) | 1.85 (1.09 to 2.88) | 0.22 (0.16 to 0.28) |
| Uganda | 55 (34 to 82) | 0.8 (0.5 to 1.18) |  | 287 (182 to 430) | 1.58 (1.01 to 2.35) | 2.42 (2.21 to 2.63) |
| Ukraine | 1272 (1121 to 1439) | 3.57 (3.14 to 4.03) |  | 978 (742 to 1271) | 2.76 (2.09 to 3.59) | -0.33 (-1.49 to 0.84) |
| United Arab Emirates | 16 (10 to 26) | 1.16 (0.69 to 1.84) |  | 154 (94 to 239) | 1.13 (0.69 to 1.74) | -0.07 (-0.2 to 0.06) |
| United Kingdom | 785 (762 to 808) | 1.85 (1.8 to 1.91) |  | 709 (683 to 739) | 1.43 (1.38 to 1.49) | -0.78 (-0.98 to -0.59) |
| United Republic of Tanzania | 113 (75 to 170) | 1.02 (0.68 to 1.52) |  | 358 (221 to 557) | 1.23 (0.76 to 1.91) | 0.64 (0.57 to 0.72) |
| United States of America | 3275 (3158 to 3387) | 1.75 (1.69 to 1.81) |  | 4190 (3981 to 4438) | 1.8 (1.71 to 1.9) | 0.23 (0.1 to 0.36) |
| United States Virgin Islands | 2 (1 to 3) | 2.26 (1.59 to 3.13) |  | 2 (1 to 3) | 2.6 (1.68 to 4) | 0.48 (0.32 to 0.63) |
| Uruguay | 52 (43 to 60) | 2.52 (2.12 to 2.96) |  | 56 (44 to 69) | 2.26 (1.8 to 2.8) | -0.48 (-0.55 to -0.4) |
| Uzbekistan | 178 (155 to 205) | 1.75 (1.53 to 2) |  | 318 (248 to 404) | 1.34 (1.04 to 1.7) | -0.67 (-1.05 to -0.3) |
| Vanuatu | 1 (1 to 2) | 1.25 (0.71 to 1.97) |  | 3 (2 to 4) | 1.61 (0.91 to 2.49) | 0.91 (0.66 to 1.16) |
| Venezuela (Bolivarian Republic of) | 123 (107 to 141) | 1.17 (1.02 to 1.34) |  | 315 (219 to 441) | 1.49 (1.03 to 2.09) | 0.77 (0.05 to 1.5) |
| Viet Nam | 363 (239 to 525) | 1.17 (0.78 to 1.69) |  | 1680 (1092 to 2487) | 2.12 (1.38 to 3.14) | 2.12 (2.05 to 2.19) |
| Yemen | 45 (24 to 78) | 0.82 (0.43 to 1.4) |  | 163 (95 to 254) | 0.97 (0.57 to 1.5) | 0.64 (0.54 to 0.74) |
| Zambia | 50 (31 to 74) | 1.5 (0.93 to 2.22) |  | 174 (104 to 266) | 1.86 (1.12 to 2.85) | 0.79 (0.64 to 0.94) |
| Zimbabwe | 47 (34 to 63) | 1.07 (0.78 to 1.44) |  | 152 (93 to 232) | 1.84 (1.12 to 2.78) | 1.91 (1.57 to 2.26) |

UI: uncertainty interval, CI: confidence interval, AAPC, average annual percent change.
