# Supplementary material for: The global, regional, and national early-onset colorectal cancer burden and trends from 1990 to 2019: results from the Global Burden of Disease Study 2019
Source: BMC Public Health. 2022 Oct 12;22:1896. doi: 10.1186/s12889-022-14274-7 (PMC9555189; doi:10.1186/s12889-022-14274-7)
Supplement: Supplementary file 9 — Additional file 9: Table S4. DALY of early-onset colorectal cancer in 1990 and 2019 with AAPC from 2009 and 2019 at countries/territories level, both sexes. [file 12889_2022_14274_MOESM9_ESM.docx]

Table S4. DALY of early-onset colorectal cancer in 1990 and 2019 with AAPC from 2009 and 2019 at countries/territories level, both sexes.

| Countries/territories | 1990 | |  | 2019 | | AAPC % (95% CI)  1990-2019 |
| --- | --- | --- | --- | --- | --- | --- |
|  | Cases (95% UI) | Age-standardized DALY per  100 000 population (95% UI) |  | Cases (95% UI) | Age-standardized DALY per  100 000 population (95% UI) |  |
| Afghanistan | 4059 (1606 to 7558) | 77.78 (30.39 to 145.72) |  | 14874 (7594 to 24472) | 75.26 (38.05 to 124.12) | -0.08 (-0.13 to -0.03) |
| Albania | 1378 (1073 to 1723) | 65.82 (51.84 to 81.56) |  | 1272 (852 to 1832) | 70.83 (47.27 to 102.08) | 0.1 (-0.25 to 0.46) |
| Algeria | 6263 (4355 to 8824) | 51.95 (36.35 to 72.73) |  | 14218 (9413 to 20607) | 44.74 (29.62 to 64.86) | -0.54 (-0.64 to -0.44) |
| American Samoa | 29 (20 to 42) | 104.48 (70.44 to 148.85) |  | 42 (27 to 62) | 115.76 (75.24 to 174.15) | 0.48 (0.15 to 0.82) |
| Andorra | 47 (29 to 71) | 105.44 (65.27 to 159.02) |  | 67 (43 to 99) | 83.83 (53.11 to 124.19) | -0.84 (-0.96 to -0.73) |
| Angola | 2738 (1590 to 4514) | 53.12 (31.08 to 87.62) |  | 8865 (5326 to 13330) | 58.64 (35.6 to 87.88) | 0.32 (0.02 to 0.61) |
| Antigua and Barbuda | 23 (18 to 29) | 60.74 (47.58 to 76.8) |  | 43 (32 to 57) | 59.41 (43.76 to 79.4) | -0.04 (-0.43 to 0.35) |
| Argentina | 21181 (18677 to 23985) | 98.5 (86.89 to 111.49) |  | 38582 (31782 to 46413) | 117.22 (96.47 to 141.17) | 0.53 (0.39 to 0.68) |
| Armenia | 2465 (2062 to 2904) | 122.2 (102.94 to 142.91) |  | 1698 (1296 to 2162) | 78.23 (59.72 to 99.5) | -1.64 (-2.68 to -0.59) |
| Australia | 12518 (11300 to 13811) | 99.22 (89.56 to 109.47) |  | 14145 (11625 to 17118) | 77.42 (63.45 to 93.92) | -0.67 (-0.94 to -0.39) |
| Austria | 6992 (6156 to 7904) | 118.91 (104.51 to 134.65) |  | 3012 (2419 to 3682) | 43.72 (34.98 to 53.64) | -3.62 (-3.92 to -3.32) |
| Azerbaijan | 6059 (4902 to 7458) | 147.05 (120.16 to 179.68) |  | 8196 (5667 to 11545) | 104.19 (72.01 to 146.82) | -0.97 (-1.58 to -0.36) |
| Bahamas | 198 (161 to 242) | 122.15 (99.68 to 148.56) |  | 405 (298 to 548) | 134.37 (98.71 to 182.03) | 0.23 (-0.22 to 0.69) |
| Bahrain | 189 (132 to 259) | 55.69 (39.23 to 75.96) |  | 778 (520 to 1121) | 48.67 (32.58 to 70.38) | -0.34 (-0.63 to -0.05) |
| Bangladesh | 17021 (10407 to 25481) | 31.29 (19.49 to 46.35) |  | 30324 (17865 to 49240) | 27.35 (16.15 to 44.28) | -0.43 (-0.71 to -0.14) |
| Barbados | 174 (144 to 209) | 104.43 (86.44 to 124.56) |  | 248 (183 to 328) | 112.01 (82.79 to 148.33) | 0.45 (0.09 to 0.82) |
| Belarus | 8756 (7613 to 10024) | 128.95 (112.23 to 147.46) |  | 5746 (3927 to 8101) | 78.52 (53.59 to 110.78) | -1.99 (-2.74 to -1.24) |
| Belgium | 6179 (5341 to 7113) | 87.53 (75.62 to 100.8) |  | 4521 (3628 to 5573) | 54.05 (43.2 to 66.89) | -1.76 (-2.11 to -1.41) |
| Belize | 30 (24 to 37) | 34.12 (27.14 to 41.91) |  | 200 (154 to 254) | 71.35 (55.28 to 90.62) | 2.31 (1.88 to 2.75) |
| Benin | 778 (528 to 1112) | 35.5 (24.29 to 50.41) |  | 2568 (1564 to 3958) | 40.05 (24.69 to 61.17) | 0.44 (0.24 to 0.63) |
| Bermuda | 51 (40 to 64) | 106.22 (82.53 to 133.51) |  | 38 (27 to 53) | 76.96 (54.38 to 105.5) | -1.2 (-1.4 to -1) |
| Bhutan | 98 (47 to 167) | 32.51 (15.99 to 55.32) |  | 189 (85 to 316) | 35.24 (16.09 to 58.84) | 0.23 (0.13 to 0.34) |
| Bolivia (Plurinational State of) | 1824 (1178 to 2594) | 52.56 (33.98 to 74.53) |  | 4496 (2593 to 7013) | 57.72 (33.49 to 89.77) | 0.34 (0.23 to 0.46) |
| Bosnia and Herzegovina | 2973 (2467 to 3607) | 94.44 (78.58 to 114.25) |  | 2812 (2007 to 3837) | 113.36 (80.7 to 154.89) | 0.39 (-0.02 to 0.81) |
| Botswana | 381 (225 to 609) | 61.87 (36.74 to 98.08) |  | 1592 (875 to 2608) | 93.8 (51.73 to 153.03) | 1.38 (1.12 to 1.64) |
| Brazil | 62713 (59065 to 66663) | 68.72 (64.8 to 72.95) |  | 136695 (126596 to 147219) | 81.28 (75.25 to 87.56) | 0.64 (0.5 to 0.78) |
| Brunei Darussalam | 257 (180 to 352) | 151.97 (107.24 to 207.6) |  | 552 (399 to 743) | 145.02 (104.82 to 195.28) | -0.06 (-0.6 to 0.48) |
| Bulgaria | 9030 (7838 to 10343) | 140.56 (121.63 to 161.51) |  | 7376 (5181 to 10169) | 132.81 (93.31 to 183.19) | -0.12 (-0.56 to 0.33) |
| Burkina Faso | 1347 (868 to 2008) | 31.33 (20.37 to 46.38) |  | 4546 (2855 to 6771) | 39.68 (25.16 to 58.72) | 0.89 (0.64 to 1.14) |
| Burundi | 1511 (941 to 2276) | 59.93 (37.32 to 90.03) |  | 2873 (1747 to 4530) | 48.84 (29.65 to 77.03) | -0.78 (-1 to -0.57) |
| Cabo Verde | 54 (38 to 75) | 38.13 (27.09 to 51.66) |  | 187 (121 to 283) | 48.29 (31.38 to 72.78) | 0.77 (0.6 to 0.94) |
| Cambodia | 3818 (2333 to 5692) | 75.88 (46.9 to 112.33) |  | 10827 (7200 to 15998) | 98.8 (65.78 to 145.73) | 0.88 (0.74 to 1.02) |
| Cameroon | 2594 (1749 to 3649) | 52.51 (35.63 to 73.51) |  | 9390 (5528 to 14742) | 59.15 (35.04 to 92.87) | 0.41 (0.29 to 0.52) |
| Canada | 16829 (15284 to 18531) | 80.03 (72.67 to 88.18) |  | 18094 (14535 to 22216) | 69.34 (55.59 to 85.35) | -0.47 (-0.68 to -0.25) |
| Central African Republic | 700 (424 to 1048) | 52.26 (31.71 to 77.96) |  | 1344 (784 to 2148) | 46.91 (27.36 to 74.96) | -0.45 (-0.77 to -0.12) |
| Chad | 816 (528 to 1186) | 29.82 (19.51 to 43.06) |  | 2858 (1792 to 4245) | 39.07 (24.68 to 57.85) | 1 (0.89 to 1.12) |
| Chile | 5044 (4254 to 5934) | 57.93 (49.01 to 67.95) |  | 9072 (7279 to 11147) | 66.31 (53.11 to 81.64) | 0.72 (0.26 to 1.19) |
| China | 770542 (655628 to 898419) | 96.06 (81.75 to 112.05) |  | 1308591 (1096385 to 1556940) | 110.29 (92.57 to 130.86) | 0.61 (0.25 to 0.98) |
| Colombia | 11799 (10190 to 13524) | 59.41 (51.55 to 67.74) |  | 25123 (17396 to 35412) | 73.7 (51.03 to 103.89) | 0.88 (0.72 to 1.03) |
| Comoros | 105 (27 to 182) | 48.93 (13.25 to 83.99) |  | 259 (138 to 408) | 55.43 (29.7 to 86.95) | -0.08 (-1.96 to 1.84) |
| Congo | 931 (491 to 1516) | 83.93 (44.5 to 136.38) |  | 2364 (1360 to 3728) | 68.81 (39.61 to 108.4) | -0.49 (-0.79 to -0.19) |
| Cook Islands | 6 (4 to 9) | 54.9 (34.87 to 82.48) |  | 6 (3 to 9) | 50.7 (26.89 to 77.87) | -0.24 (-0.35 to -0.13) |
| Costa Rica | 1016 (842 to 1215) | 57.77 (48.04 to 68.75) |  | 3505 (2473 to 4857) | 101.12 (71.33 to 140.17) | 2.29 (1.88 to 2.71) |
| Croatia | 3898 (3278 to 4594) | 107.48 (90.3 to 126.8) |  | 2879 (2011 to 4001) | 90.93 (63.53 to 126.38) | -0.5 (-0.81 to -0.19) |
| Cuba | 6185 (5329 to 7201) | 81.37 (70.25 to 94.55) |  | 6682 (4914 to 8881) | 73.75 (54.08 to 98.46) | -0.23 (-0.55 to 0.09) |
| Cyprus | 240 (175 to 322) | 43.12 (31.43 to 57.77) |  | 494 (362 to 653) | 45.28 (33.19 to 59.95) | -0.06 (-0.4 to 0.28) |
| Czechia | 11803 (10616 to 13132) | 143.62 (128.87 to 160.15) |  | 6523 (4790 to 8617) | 70.88 (51.96 to 93.83) | -2.64 (-2.88 to -2.4) |
| C么te d'Ivoire | 3019 (1962 to 4446) | 50.9 (33.26 to 74.71) |  | 7650 (4649 to 11619) | 49.72 (30.39 to 75.28) | -0.08 (-0.24 to 0.08) |
| Democratic People's Republic of Korea | 16367 (9804 to 25124) | 115.91 (69.47 to 177.86) |  | 23101 (13198 to 38217) | 108.36 (61.67 to 179.84) | -0.27 (-0.32 to -0.22) |
| Democratic Republic of the Congo | 7081 (4496 to 10689) | 40.21 (25.85 to 60.06) |  | 16505 (9664 to 26770) | 36.58 (21.4 to 58.93) | -0.36 (-0.51 to -0.2) |
| Denmark | 3927 (3450 to 4464) | 94.6 (82.8 to 107.9) |  | 2620 (2116 to 3210) | 63.36 (50.92 to 77.95) | -1.39 (-1.93 to -0.84) |
| Djibouti | 126 (73 to 196) | 54.74 (32.06 to 85.28) |  | 573 (311 to 945) | 68.1 (37.12 to 112.1) | 0.79 (0.69 to 0.89) |
| Dominica | 28 (20 to 37) | 69 (50.33 to 91.47) |  | 39 (26 to 55) | 81.16 (54.52 to 116.81) | 0.56 (0.42 to 0.69) |
| Dominican Republic | 2191 (1629 to 2886) | 54.43 (40.89 to 71.21) |  | 7031 (4396 to 10536) | 92.12 (57.61 to 138.05) | 2.16 (1.87 to 2.46) |
| Ecuador | 2584 (2100 to 3147) | 45.15 (37.01 to 54.56) |  | 8788 (6080 to 12412) | 73.11 (50.62 to 103.2) | 1.45 (1.11 to 1.79) |
| Egypt | 17274 (12914 to 22820) | 52.95 (40.17 to 69.2) |  | 39031 (24247 to 58391) | 59.57 (36.98 to 89.1) | 0.42 (0.2 to 0.64) |
| El Salvador | 1225 (979 to 1517) | 43.69 (35.2 to 53.67) |  | 3311 (2196 to 4826) | 79.08 (52.48 to 115.16) | 2.12 (1.74 to 2.51) |
| Equatorial Guinea | 90 (52 to 144) | 44.45 (25.8 to 70.81) |  | 540 (268 to 956) | 74.9 (37.54 to 132.53) | 1.87 (1.62 to 2.13) |
| Eritrea | 725 (437 to 1088) | 51.77 (31.21 to 77.49) |  | 2759 (1686 to 4288) | 73.66 (45.27 to 113.81) | 1.22 (0.91 to 1.53) |
| Estonia | 1127 (938 to 1340) | 101.82 (84.7 to 121.14) |  | 708 (488 to 998) | 71.75 (49.36 to 101.2) | -1.29 (-2.38 to -0.19) |
| Eswatini | 200 (128 to 300) | 54.66 (35.1 to 81.53) |  | 604 (319 to 1021) | 89.98 (47.72 to 152.27) | 1.64 (1.41 to 1.87) |
| Ethiopia | 15199 (9113 to 23220) | 64.95 (39.45 to 99.25) |  | 26441 (18650 to 37676) | 48.94 (34.49 to 69.92) | -1.06 (-1.26 to -0.86) |
| Fiji | 326 (223 to 461) | 68.26 (46.92 to 96.49) |  | 490 (324 to 714) | 76.79 (50.82 to 111.85) | 0.47 (0.19 to 0.74) |
| Finland | 2634 (2273 to 3062) | 63.81 (54.95 to 74.33) |  | 1701 (1341 to 2126) | 46.12 (36.3 to 57.76) | -1.32 (-1.63 to -1.01) |
| France | 33900 (30439 to 37590) | 83.06 (74.6 to 92.06) |  | 28391 (22868 to 34626) | 60.1 (48.24 to 73.56) | -1.23 (-1.69 to -0.76) |
| Gabon | 426 (227 to 730) | 91.72 (49.2 to 157.45) |  | 919 (533 to 1464) | 82.3 (47.78 to 130.89) | -0.42 (-0.62 to -0.21) |
| Gambia | 116 (71 to 177) | 25.35 (15.64 to 38.44) |  | 392 (232 to 614) | 33.53 (19.89 to 52.26) | 1.14 (0.41 to 1.89) |
| Georgia | 4385 (3542 to 5351) | 104.83 (84.72 to 127.88) |  | 2869 (2081 to 3855) | 74.35 (53.88 to 99.9) | -1.71 (-2.42 to -0.98) |
| Germany | 54876 (49634 to 60298) | 92.27 (83.34 to 101.54) |  | 38697 (31558 to 46737) | 65.86 (53.54 to 79.78) | -1.76 (-2.42 to -1.1) |
| Ghana | 3426 (2264 to 4931) | 43.51 (28.89 to 62.22) |  | 11559 (7142 to 17485) | 57.25 (35.64 to 86.28) | 0.99 (0.84 to 1.14) |
| Greece | 3782 (3272 to 4331) | 51.8 (44.72 to 59.43) |  | 4511 (3797 to 5317) | 54.71 (45.79 to 64.89) | 0.02 (-0.18 to 0.23) |
| Greenland | 74 (52 to 102) | 171.09 (120.37 to 233.86) |  | 58 (40 to 83) | 152.47 (105.62 to 218.69) | -0.57 (-0.8 to -0.34) |
| Grenada | 37 (28 to 48) | 88.21 (67.14 to 113.69) |  | 72 (52 to 95) | 95.48 (69.3 to 126.93) | 0.23 (0.02 to 0.44) |
| Guam | 89 (62 to 127) | 95.95 (66.53 to 135.25) |  | 144 (98 to 206) | 126.91 (86.23 to 181.33) | 1.02 (0.92 to 1.12) |
| Guatemala | 1599 (1279 to 1972) | 39.76 (31.92 to 48.88) |  | 8355 (6032 to 11228) | 74.25 (53.69 to 99.7) | 2.25 (1.84 to 2.66) |
| Guinea | 976 (672 to 1358) | 31.81 (22.04 to 43.94) |  | 2537 (1594 to 3848) | 40.34 (25.49 to 61.04) | 0.85 (0.72 to 0.98) |
| Guinea-Bissau | 271 (165 to 412) | 58.09 (35.51 to 88.51) |  | 590 (374 to 872) | 58.64 (36.94 to 86.64) | 0.02 (-0.05 to 0.09) |
| Guyana | 346 (259 to 451) | 79.85 (59.72 to 103.89) |  | 611 (406 to 881) | 115.1 (76.42 to 166.02) | 1.19 (0.55 to 1.83) |
| Haiti | 2526 (1433 to 3844) | 74.2 (42.76 to 112.08) |  | 5355 (3123 to 8378) | 67.03 (39.18 to 104.87) | -0.33 (-0.48 to -0.18) |
| Honduras | 1027 (716 to 1429) | 44.56 (31.55 to 61.23) |  | 2469 (1342 to 4174) | 41.43 (22.74 to 69.52) | -0.2 (-0.45 to 0.06) |
| Hungary | 11607 (10217 to 13217) | 146.37 (128.44 to 167.19) |  | 8869 (6619 to 11761) | 108.66 (80.94 to 144.32) | -1.22 (-1.98 to -0.45) |
| Iceland | 99 (80 to 120) | 57.35 (46.34 to 69.78) |  | 107 (82 to 136) | 43.12 (33.11 to 55.02) | -0.98 (-1.18 to -0.78) |
| India | 205966 (174805 to 241390) | 40.42 (34.5 to 47.2) |  | 492973 (404840 to 593941) | 50 (41.06 to 60.23) | 0.78 (0.54 to 1.02) |
| Indonesia | 83531 (59455 to 105306) | 75.39 (53.96 to 94.81) |  | 206806 (145914 to 274673) | 100.28 (70.86 to 133.09) | 0.97 (0.92 to 1.03) |
| Iran (Islamic Republic of) | 14930 (12100 to 18435) | 52.8 (43.03 to 65.13) |  | 42021 (38136 to 46580) | 59.69 (54.14 to 66.28) | 0.44 (0.32 to 0.55) |
| Iraq | 4613 (2825 to 7253) | 54.83 (33.75 to 85.85) |  | 17566 (11476 to 26368) | 63.17 (41.33 to 94.72) | 0.63 (0.42 to 0.85) |
| Ireland | 2286 (1937 to 2680) | 96.75 (82.06 to 113.29) |  | 2232 (1738 to 2821) | 56.53 (43.89 to 71.68) | -1.74 (-2.47 to -1.01) |
| Israel | 2561 (2144 to 3065) | 83.22 (69.93 to 99.2) |  | 4140 (3323 to 5119) | 64.97 (52.09 to 80.43) | -0.85 (-1.2 to -0.5) |
| Italy | 37222 (35478 to 38959) | 89.92 (85.66 to 94.17) |  | 28849 (26613 to 31034) | 59.56 (54.87 to 64.18) | -1.42 (-1.63 to -1.21) |
| Jamaica | 530 (413 to 666) | 42.84 (33.47 to 53.57) |  | 2052 (1396 to 2889) | 101.16 (68.78 to 142.34) | 3.84 (2.54 to 5.15) |
| Japan | 108670 (105317 to 112007) | 103.17 (99.91 to 106.43) |  | 69779 (65112 to 73551) | 71.39 (66.54 to 75.34) | -1.35 (-1.63 to -1.07) |
| Jordan | 1369 (961 to 1896) | 81.95 (57.86 to 112.98) |  | 5644 (4076 to 7676) | 70.19 (50.76 to 95.33) | -0.43 (-0.6 to -0.27) |
| Kazakhstan | 11452 (9988 to 13024) | 115.56 (101.21 to 130.85) |  | 9633 (7552 to 12075) | 70.69 (55.43 to 88.6) | -1.71 (-2.16 to -1.25) |
| Kenya | 3064 (2079 to 3938) | 31 (20.79 to 39.74) |  | 13855 (10322 to 18035) | 48.79 (36.43 to 63.37) | 1.56 (1.38 to 1.74) |
| Kiribati | 48 (32 to 69) | 113.86 (76.77 to 163.91) |  | 80 (50 to 124) | 105.55 (66.43 to 164.09) | -0.28 (-0.34 to -0.22) |
| Kuwait | 456 (352 to 579) | 36.91 (28.54 to 46.69) |  | 1638 (1185 to 2208) | 35.29 (25.49 to 47.61) | 0.08 (-1.02 to 1.18) |
| Kyrgyzstan | 2583 (2088 to 3146) | 109.98 (89.23 to 133.34) |  | 2152 (1618 to 2796) | 49.18 (37 to 63.91) | -2.97 (-3.51 to -2.43) |
| Lao People's Democratic Republic | 1996 (1078 to 3144) | 94.83 (51.58 to 148.76) |  | 4923 (2881 to 7482) | 102.08 (59.89 to 155.21) | 0.26 (0.2 to 0.33) |
| Latvia | 1990 (1697 to 2322) | 103.95 (88.44 to 121.59) |  | 987 (708 to 1344) | 70.43 (50.49 to 96.13) | -1.15 (-3.09 to 0.83) |
| Lebanon | 1464 (945 to 2144) | 80.34 (52.02 to 117.4) |  | 3365 (2215 to 4832) | 88.76 (58.5 to 127.36) | 0.37 (0.27 to 0.48) |
| Lesotho | 292 (174 to 489) | 32.52 (19.55 to 53.9) |  | 978 (559 to 1532) | 75.32 (43.05 to 117.72) | 3.17 (2.74 to 3.6) |
| Liberia | 367 (240 to 537) | 36.57 (24.02 to 53.2) |  | 1047 (587 to 1697) | 34.73 (19.5 to 56.24) | -0.29 (-0.55 to -0.04) |
| Libya | 1774 (1089 to 2821) | 88.2 (54.45 to 139.48) |  | 5528 (3564 to 8271) | 90.19 (58.11 to 134.96) | -0.09 (-0.42 to 0.24) |
| Lithuania | 2636 (2269 to 3054) | 102.4 (88.07 to 118.72) |  | 1465 (1063 to 1981) | 72.95 (52.84 to 98.92) | -0.85 (-3.06 to 1.42) |
| Luxembourg | 298 (251 to 350) | 100.88 (84.82 to 118.78) |  | 269 (215 to 333) | 52.41 (41.74 to 64.97) | -2.28 (-2.46 to -2.11) |
| Madagascar | 3331 (2081 to 4950) | 58.63 (37.35 to 86.1) |  | 7618 (4773 to 11439) | 51.53 (32.17 to 77.54) | -0.48 (-0.92 to -0.04) |
| Malawi | 1353 (910 to 1912) | 29.73 (20.19 to 41.78) |  | 3272 (1945 to 5193) | 34.66 (20.79 to 54.7) | 0.5 (0.29 to 0.71) |
| Malaysia | 8962 (6639 to 11787) | 85.65 (63.88 to 112.19) |  | 22143 (14735 to 31931) | 96.94 (64.57 to 139.78) | 0.41 (-0.05 to 0.88) |
| Maldives | 53 (28 to 82) | 56.32 (29.84 to 86.56) |  | 158 (107 to 225) | 35.77 (24.38 to 50.76) | -1.67 (-1.88 to -1.47) |
| Mali | 1706 (1208 to 2339) | 41.1 (29.25 to 56.07) |  | 4546 (2800 to 6905) | 44.9 (27.87 to 67.65) | 0.35 (0.16 to 0.53) |
| Malta | 154 (124 to 191) | 53.16 (42.63 to 65.89) |  | 169 (128 to 220) | 52.47 (39.57 to 68.21) | 0.01 (-0.29 to 0.31) |
| Marshall Islands | 19 (12 to 27) | 87.64 (59.25 to 122.94) |  | 44 (26 to 67) | 109.34 (65.19 to 167.82) | 0.77 (0.72 to 0.82) |
| Mauritania | 454 (297 to 654) | 45.7 (29.95 to 65.72) |  | 775 (445 to 1232) | 35.59 (20.61 to 56.2) | -0.84 (-0.99 to -0.68) |
| Mauritius | 382 (316 to 458) | 53.09 (44.02 to 63.32) |  | 789 (581 to 1061) | 80.15 (58.95 to 107.73) | 1.4 (0.87 to 1.92) |
| Mexico | 21865 (20848 to 22908) | 45.61 (43.56 to 47.7) |  | 66582 (56352 to 77943) | 72.13 (61.07 to 84.42) | 1.91 (1.61 to 2.21) |
| Micronesia (Federated States of) | 55 (34 to 82) | 105.86 (64.61 to 158.11) |  | 74 (23 to 123) | 109.15 (32.17 to 182.34) | 0.05 (-0.03 to 0.12) |
| Monaco | 23 (16 to 32) | 96.01 (66.49 to 134.32) |  | 25 (16 to 36) | 97.31 (63.79 to 143.07) | 0.06 (0.04 to 0.08) |
| Mongolia | 701 (483 to 989) | 66.79 (46.23 to 93.91) |  | 1871 (1233 to 2760) | 69.93 (46.06 to 103.16) | 0.05 (-0.14 to 0.25) |
| Montenegro | 345 (259 to 449) | 82.52 (61.94 to 106.96) |  | 339 (251 to 443) | 72.77 (53.82 to 95.49) | -0.64 (-1.24 to -0.04) |
| Morocco | 5476 (3801 to 7624) | 40.2 (28.09 to 55.64) |  | 12227 (7615 to 19473) | 46.06 (28.69 to 73.35) | 0.47 (0.33 to 0.61) |
| Mozambique | 1732 (1134 to 2515) | 26.76 (17.61 to 38.76) |  | 6601 (3860 to 10318) | 47.98 (28 to 75) | 2.16 (1.97 to 2.36) |
| Myanmar | 20016 (12210 to 30351) | 87.38 (53.74 to 132.31) |  | 39539 (24609 to 59895) | 99.82 (62.12 to 151.21) | 0.55 (0.34 to 0.76) |
| Namibia | 233 (134 to 353) | 34.67 (20.37 to 52.09) |  | 680 (387 to 1091) | 46.04 (26.45 to 73.94) | 1.26 (0.85 to 1.66) |
| Nauru | 10 (5 to 17) | 164.85 (85.55 to 274.46) |  | 11 (6 to 16) | 152.91 (82.76 to 235.38) | -0.27 (-0.44 to -0.1) |
| Nepal | 3016 (1778 to 4770) | 29.24 (17.26 to 46.11) |  | 5832 (3334 to 9292) | 30.35 (17.45 to 48.11) | 0.17 (0.05 to 0.3) |
| Netherlands | 10267 (9165 to 11471) | 88.26 (78.74 to 98.68) |  | 8789 (7101 to 10672) | 70.07 (56.33 to 85.44) | -0.91 (-1.15 to -0.67) |
| New Zealand | 3093 (2669 to 3584) | 126.97 (109.66 to 146.98) |  | 2707 (2236 to 3253) | 85.97 (70.86 to 103.66) | -1.37 (-1.67 to -1.08) |
| Nicaragua | 842 (601 to 1148) | 44.75 (32.6 to 60.09) |  | 2266 (1572 to 3173) | 51.52 (35.9 to 71.84) | 0.31 (-0.16 to 0.77) |
| Niger | 1181 (747 to 1776) | 31.4 (20 to 46.99) |  | 2827 (1687 to 4512) | 29.19 (17.75 to 46.04) | -0.15 (-0.3 to 0) |
| Nigeria | 13114 (8800 to 19177) | 28.9 (19.48 to 42.13) |  | 38151 (26191 to 54337) | 34.54 (23.78 to 49.01) | 0.59 (0.37 to 0.81) |
| Niue | 1 (1 to 2) | 98.08 (61.08 to 148.08) |  | 1 (1 to 2) | 93.54 (47.9 to 158.11) | -0.19 (-0.43 to 0.06) |
| North Macedonia | 1302 (1051 to 1600) | 92.84 (75.01 to 113.94) |  | 1773 (1270 to 2439) | 101.31 (72.4 to 139.42) | 0.26 (-0.11 to 0.62) |
| Northern Mariana Islands | 50 (30 to 78) | 135.86 (82.29 to 209.45) |  | 38 (24 to 57) | 118.55 (74.76 to 181.16) | -0.38 (-0.7 to -0.06) |
| Norway | 2951 (2787 to 3126) | 93.56 (88.34 to 99.12) |  | 2634 (2360 to 2870) | 64.74 (57.92 to 70.64) | -1.29 (-1.58 to -1.01) |
| Oman | 408 (239 to 641) | 36.96 (21.64 to 57.98) |  | 1287 (833 to 2064) | 32.26 (20.94 to 51.75) | -0.53 (-0.73 to -0.33) |
| Pakistan | 22621 (16595 to 29945) | 41.06 (30.28 to 54.04) |  | 87646 (61954 to 121851) | 67.7 (47.95 to 93.99) | 1.77 (1.58 to 1.95) |
| Palau | 8 (5 to 13) | 81.35 (50.76 to 122) |  | 12 (8 to 18) | 76.93 (48.92 to 114.64) | -0.28 (-0.46 to -0.09) |
| Palestine | 883 (536 to 1365) | 103.35 (62.93 to 159.31) |  | 2870 (2116 to 3803) | 99.89 (73.95 to 131.77) | 0 (-0.15 to 0.14) |
| Panama | 732 (586 to 902) | 51 (40.94 to 62.72) |  | 1935 (1301 to 2738) | 66.49 (44.69 to 94.12) | 0.91 (0.52 to 1.31) |
| Papua New Guinea | 1175 (720 to 1798) | 51.52 (31.68 to 78.54) |  | 3761 (2334 to 5808) | 59.4 (36.81 to 91.78) | 0.45 (0.41 to 0.48) |
| Paraguay | 926 (694 to 1198) | 41.72 (31.49 to 53.59) |  | 3627 (2383 to 5313) | 77.37 (50.87 to 113.27) | 2.34 (1.79 to 2.9) |
| Peru | 6319 (4763 to 8205) | 51.22 (38.97 to 66.03) |  | 13803 (8839 to 20307) | 56.36 (36.09 to 82.94) | 0.34 (-0.12 to 0.8) |
| Philippines | 46152 (40221 to 51671) | 128.47 (112.21 to 143.57) |  | 102708 (83208 to 127187) | 136.45 (110.5 to 169.07) | 0.24 (0.1 to 0.37) |
| Poland | 29491 (28133 to 30907) | 110.85 (105.8 to 116.13) |  | 27000 (22186 to 32332) | 87.08 (71.59 to 104.2) | -1.19 (-1.85 to -0.53) |
| Portugal | 7606 (6543 to 8769) | 110 (94.52 to 126.94) |  | 7595 (6120 to 9334) | 88.6 (71.05 to 109.29) | -0.85 (-1.51 to -0.19) |
| Puerto Rico | 2027 (1672 to 2431) | 81.54 (67.26 to 97.8) |  | 2383 (1640 to 3414) | 94.95 (65.4 to 135.71) | 0.45 (0.04 to 0.87) |
| Qatar | 145 (94 to 217) | 38.56 (25.17 to 57.51) |  | 1025 (668 to 1516) | 35.03 (22.84 to 51.95) | -0.19 (-1.21 to 0.85) |
| Republic of Korea | 26959 (23040 to 31310) | 85.47 (73.21 to 99.07) |  | 27959 (22089 to 34848) | 60.57 (47.6 to 75.92) | -1.89 (-2.49 to -1.29) |
| Republic of Moldova | 4300 (3739 to 4953) | 145.9 (127.25 to 167.72) |  | 2905 (2255 to 3654) | 98.69 (76.49 to 124.3) | -1.49 (-2.52 to -0.45) |
| Romania | 16163 (14096 to 18488) | 103.2 (89.92 to 118.16) |  | 17204 (12880 to 22496) | 111.31 (83.09 to 145.76) | 0.18 (-0.76 to 1.14) |
| Russian Federation | 92106 (83451 to 98448) | 92.47 (83.85 to 98.78) |  | 109603 (92232 to 128224) | 95.09 (80.01 to 111.22) | -0.19 (-0.58 to 0.2) |
| Rwanda | 2176 (1308 to 3249) | 68.96 (41.42 to 102.34) |  | 4021 (2433 to 6341) | 56.06 (34.14 to 87.99) | -0.78 (-1.05 to -0.5) |
| Saint Kitts and Nevis | 24 (19 to 29) | 117.09 (94.17 to 142.16) |  | 33 (10 to 54) | 67.06 (20.7 to 110.13) | -1.74 (-2.06 to -1.42) |
| Saint Lucia | 50 (41 to 60) | 71.83 (59.48 to 86.55) |  | 105 (80 to 136) | 73.73 (55.98 to 95.47) | 0 (-0.34 to 0.34) |
| Saint Vincent and the Grenadines | 40 (32 to 50) | 74.12 (59.72 to 91.22) |  | 76 (59 to 97) | 90.49 (70.17 to 115.32) | 0.44 (0.27 to 0.62) |
| Samoa | 51 (32 to 77) | 65.29 (41.78 to 98.6) |  | 84 (46 to 130) | 66.61 (36.69 to 103.26) | 0.05 (-0.04 to 0.15) |
| San Marino | 12 (8 to 17) | 71.48 (50.02 to 99.62) |  | 18 (10 to 30) | 70.59 (39.79 to 118.52) | -0.04 (-0.1 to 0.01) |
| Sao Tome and Principe | 27 (14 to 42) | 49.68 (27.82 to 74.22) |  | 94 (53 to 154) | 71.25 (40.52 to 116.16) | 1.45 (1 to 1.9) |
| Saudi Arabia | 3823 (2277 to 6041) | 42.76 (25.54 to 67.38) |  | 24298 (15748 to 36415) | 67.5 (43.86 to 101.01) | 1.58 (1.52 to 1.64) |
| Senegal | 1490 (974 to 2128) | 42.62 (28.08 to 60.48) |  | 3593 (2167 to 5502) | 44 (26.75 to 67.04) | 0.01 (-0.44 to 0.46) |
| Serbia | 8587 (6293 to 11539) | 127.71 (93.39 to 171.91) |  | 7333 (5177 to 10209) | 112.52 (79.36 to 156.72) | -0.54 (-0.84 to -0.23) |
| Seychelles | 47 (35 to 63) | 116.69 (85.82 to 154.04) |  | 135 (98 to 183) | 158.91 (114.48 to 215.84) | 1.12 (0.89 to 1.35) |
| Sierra Leone | 607 (382 to 908) | 32.22 (20.37 to 47.97) |  | 1725 (1062 to 2611) | 37.98 (23.51 to 57.34) | 0.64 (0.42 to 0.87) |
| Singapore | 2688 (2237 to 3183) | 109.9 (91.61 to 129.87) |  | 2499 (1988 to 3084) | 47.26 (37.51 to 58.52) | -3.14 (-3.45 to -2.83) |
| Slovakia | 4716 (3967 to 5575) | 127.02 (106.97 to 149.94) |  | 4536 (3106 to 6372) | 98.9 (67.7 to 139.03) | -0.93 (-1.11 to -0.74) |
| Slovenia | 1444 (1022 to 1977) | 97.93 (69.31 to 134) |  | 1044 (719 to 1487) | 64.24 (44.31 to 91.41) | -1.8 (-2.09 to -1.5) |
| Solomon Islands | 161 (81 to 263) | 100.53 (50.15 to 164.6) |  | 492 (251 to 767) | 119.95 (61.25 to 186.97) | 0.66 (0.58 to 0.74) |
| Somalia | 1382 (819 to 2260) | 38 (22.48 to 62.18) |  | 3748 (2038 to 7334) | 36.83 (20.09 to 71.93) | -0.1 (-0.16 to -0.04) |
| South Africa | 17413 (14653 to 20502) | 81.08 (68.57 to 94.91) |  | 25633 (18225 to 34729) | 62.38 (44.61 to 84.35) | -1.28 (-1.82 to -0.75) |
| South Sudan | 1608 (792 to 3032) | 59.37 (29.34 to 111.74) |  | 2828 (1533 to 4903) | 58.16 (31.59 to 100.52) | -0.21 (-0.53 to 0.12) |
| Spain | 24731 (22149 to 27528) | 93.94 (84.11 to 104.61) |  | 25946 (21291 to 31575) | 65.1 (53.17 to 79.52) | -1.34 (-1.64 to -1.04) |
| Sri Lanka | 3973 (2911 to 5282) | 34.78 (25.61 to 46.1) |  | 5800 (3777 to 8609) | 36.18 (23.53 to 53.75) | 0.27 (-0.28 to 0.83) |
| Sudan | 3948 (2334 to 6122) | 40.36 (23.95 to 62.39) |  | 11653 (6287 to 19355) | 49.46 (26.72 to 81.91) | 0.69 (0.64 to 0.75) |
| Suriname | 179 (125 to 236) | 78.99 (56.29 to 103.65) |  | 431 (299 to 598) | 103.91 (72.13 to 144.58) | 0.85 (-0.16 to 1.87) |
| Sweden | 4854 (4258 to 5501) | 71.76 (62.67 to 81.66) |  | 4385 (3717 to 5152) | 60.92 (51.46 to 71.79) | -0.48 (-0.69 to -0.27) |
| Switzerland | 3375 (2892 to 3917) | 61.1 (52.2 to 71.16) |  | 2993 (2377 to 3700) | 44.4 (35.1 to 55.12) | -1.29 (-1.8 to -0.78) |
| Syrian Arab Republic | 3518 (2343 to 5039) | 56.99 (38.42 to 80.96) |  | 4512 (2988 to 6575) | 44.47 (29.37 to 64.96) | -0.69 (-1.08 to -0.29) |
| Taiwan (Province of China) | 18296 (16241 to 20495) | 129.97 (115.71 to 145.21) |  | 34536 (24839 to 47082) | 173.31 (124.55 to 236.4) | 0.81 (0.26 to 1.37) |
| Tajikistan | 3472 (2726 to 4358) | 133.22 (105.75 to 165.64) |  | 4901 (3349 to 6980) | 77.8 (53.25 to 110.7) | -1.93 (-2.43 to -1.43) |
| Thailand | 31532 (23727 to 41360) | 83.79 (63.3 to 109.66) |  | 47375 (31550 to 68585) | 81.14 (53.99 to 117.56) | -0.36 (-0.91 to 0.2) |
| Timor-Leste | 243 (138 to 388) | 55.32 (32.02 to 87.81) |  | 576 (155 to 918) | 78.22 (20.07 to 125) | 1.25 (0.86 to 1.64) |
| Togo | 692 (468 to 990) | 40.8 (27.88 to 57.96) |  | 2048 (1202 to 3188) | 43.32 (25.49 to 67.33) | 0.23 (0.07 to 0.4) |
| Tokelau | 1 (0 to 1) | 75.85 (45.61 to 117.4) |  | 1 (0 to 1) | 80.69 (42.56 to 129.86) | 0.24 (0.18 to 0.3) |
| Tonga | 17 (12 to 24) | 36.75 (25.19 to 51.28) |  | 26 (16 to 39) | 41.47 (26.1 to 62.41) | 0.42 (0.32 to 0.51) |
| Trinidad and Tobago | 681 (567 to 808) | 89.68 (75.02 to 106.12) |  | 968 (648 to 1379) | 91.3 (61.16 to 130.18) | -0.28 (-0.83 to 0.28) |
| Tunisia | 1950 (1341 to 2748) | 43.54 (30.06 to 60.98) |  | 4716 (2971 to 6967) | 51.53 (32.46 to 76.19) | 0.57 (0.48 to 0.65) |
| Turkey | 37687 (24742 to 54415) | 109.79 (72.45 to 157.8) |  | 50759 (36625 to 68639) | 76.96 (55.49 to 104.16) | -1.35 (-1.76 to -0.93) |
| Turkmenistan | 1797 (1488 to 2143) | 92.98 (77.66 to 110.08) |  | 2180 (1579 to 2965) | 60.19 (43.61 to 81.83) | -1.32 (-2.38 to -0.25) |
| Tuvalu | 5 (3 to 7) | 85.55 (56.02 to 124.45) |  | 7 (4 to 11) | 91.9 (54 to 143.49) | 0.24 (0.18 to 0.3) |
| Uganda | 2791 (1709 to 4164) | 38.64 (24.15 to 57.09) |  | 14498 (9188 to 21824) | 76.68 (48.88 to 114.71) | 2.44 (2.24 to 2.63) |
| Ukraine | 62314 (54746 to 70728) | 174.93 (153.6 to 198.65) |  | 47244 (35865 to 61399) | 135.64 (102.75 to 176.59) | -0.29 (-1.45 to 0.87) |
| United Arab Emirates | 796 (468 to 1282) | 54.81 (32.46 to 87.57) |  | 7375 (4494 to 11438) | 54.05 (33.05 to 83.5) | -0.02 (-0.14 to 0.1) |
| United Kingdom | 37599 (36431 to 38826) | 89.24 (86.45 to 92.19) |  | 34992 (33528 to 36601) | 71.75 (68.72 to 75.08) | -0.65 (-0.84 to -0.46) |
| United Republic of Tanzania | 5746 (3759 to 8625) | 49.7 (32.76 to 74.21) |  | 17847 (10988 to 27921) | 59.96 (37.03 to 93.44) | 0.62 (0.37 to 0.87) |
| United States of America | 162035 (155846 to 168009) | 86.29 (83.01 to 89.47) |  | 206031 (194611 to 219049) | 89.35 (84.34 to 95.11) | 0.27 (0.14 to 0.39) |
| United States Virgin Islands | 91 (64 to 127) | 111.42 (78.23 to 155.01) |  | 90 (59 to 138) | 125.6 (80.42 to 194.59) | 0.43 (0.28 to 0.58) |
| Uruguay | 2495 (2084 to 2942) | 122.25 (102.09 to 144.19) |  | 2715 (2149 to 3369) | 111.48 (88.09 to 138.6) | -0.4 (-0.47 to -0.33) |
| Uzbekistan | 9555 (8261 to 11057) | 88.92 (77.56 to 101.97) |  | 16243 (12617 to 20625) | 67.74 (52.65 to 85.96) | -0.72 (-1.09 to -0.35) |
| Vanuatu | 49 (28 to 77) | 61.48 (35.08 to 96.92) |  | 141 (79 to 219) | 80.01 (45.06 to 123.78) | 0.93 (0.67 to 1.19) |
| Venezuela (Bolivarian Republic of) | 6295 (5441 to 7261) | 57.79 (50.26 to 66.3) |  | 15721 (10937 to 21986) | 75.14 (52.24 to 105.1) | 0.89 (0.13 to 1.65) |
| Viet Nam | 18230 (12000 to 26532) | 56.42 (37.44 to 81.58) |  | 80506 (52071 to 119687) | 101.96 (65.89 to 151.68) | 2.11 (2.04 to 2.18) |
| Yemen | 2229 (1142 to 3852) | 39.14 (20.42 to 67.11) |  | 8037 (4657 to 12562) | 46.36 (27.12 to 72.09) | 0.66 (0.56 to 0.75) |
| Zambia | 2534 (1554 to 3780) | 73.02 (45.08 to 108.3) |  | 8675 (5220 to 13262) | 90.31 (54.15 to 138.15) | 0.77 (0.63 to 0.91) |
| Zimbabwe | 2386 (1709 to 3227) | 52.18 (37.75 to 70.03) |  | 7541 (4577 to 11540) | 88.78 (54.05 to 135.1) | 1.88 (1.53 to 2.23) |

UI: uncertainty interval, CI: confidence interval, AAPC, average annual percent change.
