# Supplementary material for: The global, regional, and national early-onset colorectal cancer burden and trends from 1990 to 2019: results from the Global Burden of Disease Study 2019
Source: BMC Public Health. 2022 Oct 12;22:1896. doi: 10.1186/s12889-022-14274-7 (PMC9555189; doi:10.1186/s12889-022-14274-7)
Supplement: Supplementary file 11 — Additional file 11: Table S6. Frontier DALYs, and effective difference by country or territory. [file 12889_2022_14274_MOESM11_ESM.docx]

Table S6: Frontier DALYs, and effective difference by country or territory.

| **Location** | **SDI** | **Age standardized DALYs** | **Frontier DALYs** | **Effective difference** | **Effective difference rank (Age standardized DALYs rank)** |
| --- | --- | --- | --- | --- | --- |
| Afghanistan | 0.343 | 75.26 (38.05 to 124.12) | 24.19 | 51.07 | 122 (122) |
| Albania | 0.681 | 70.83 (47.27 to 102.08) | 24.19 | 46.64 | 103 (103) |
| Algeria | 0.652 | 44.74 (29.62 to 64.86) | 24.21 | 20.53 | 31 (30) |
| American Samoa | 0.712 | 115.76 (75.24 to 174.15) | 24.21 | 91.55 | 190 (190) |
| Andorra | 0.894 | 83.83 (53.11 to 124.19) | 24.21 | 59.62 | 143 (143) |
| Angola | 0.47 | 58.64 (35.6 to 87.88) | 24.18 | 34.46 | 66 (65.5) |
| Antigua and Barbuda | 0.743 | 59.41 (43.76 to 79.4) | 24.19 | 35.22 | 69 (69) |
| Argentina | 0.708 | 117.22 (96.47 to 141.17) | 24.18 | 93.04 | 191 (191) |
| Armenia | 0.689 | 78.23 (59.72 to 99.5) | 24.2 | 54.03 | 133 (133) |
| Australia | 0.839 | 77.42 (63.45 to 93.92) | 24.18 | 53.24 | 130 (130) |
| Austria | 0.849 | 43.72 (34.98 to 53.64) | 24.2 | 19.52 | 26 (26) |
| Azerbaijan | 0.683 | 104.19 (72.01 to 146.82) | 24.21 | 79.98 | 177 (177) |
| Bahrain | 0.751 | 48.67 (32.58 to 70.38) | 24.18 | 24.49 | 42 (41) |
| Bangladesh | 0.483 | 27.35 (16.15 to 44.28) | 24.19 | 3.16 | 2 (1) |
| Barbados | 0.742 | 112.01 (82.79 to 148.33) | 24.2 | 87.81 | 186 (186) |
| Belarus | 0.745 | 78.52 (53.59 to 110.78) | 24.2 | 54.32 | 134 (134) |
| Belgium | 0.851 | 54.05 (43.2 to 66.89) | 24.2 | 29.85 | 56 (55.5) |
| Belize | 0.603 | 71.35 (55.28 to 90.62) | 24.21 | 47.14 | 105 (105) |
| Benin | 0.352 | 40.05 (24.69 to 61.17) | 24.21 | 15.84 | 20 (20) |
| Bermuda | 0.813 | 76.96 (54.38 to 105.5) | 24.2 | 52.76 | 127 (127.5) |
| Bhutan | 0.455 | 35.24 (16.09 to 58.84) | 24.2 | 11.04 | 11 (10) |
| Bolivia | 0.566 | 57.72 (33.49 to 89.77) | 24.21 | 33.51 | 63 (63) |
| Bosnia and Herzegovina | 0.718 | 113.36 (80.7 to 154.89) | 24.21 | 89.15 | 188 (188) |
| Botswana | 0.634 | 93.8 (51.73 to 153.03) | 24.18 | 69.62 | 159 (159) |
| Brazil | 0.64 | 81.28 (75.25 to 87.56) | 24.2 | 57.08 | 141 (141) |
| Brunei | 0.823 | 145.02 (104.82 to 195.28) | 24.21 | 120.81 | 200 (200) |
| Bulgaria | 0.764 | 132.81 (93.31 to 183.19) | 24.18 | 108.63 | 196 (196) |
| Burkina Faso | 0.257 | 39.68 (25.16 to 58.72) | 25.34 | 14.34 | 19 (19) |
| Burundi | 0.284 | 48.84 (29.65 to 77.03) | 25.17 | 23.67 | 39 (43) |
| C?te d'Ivoire | 0.408 | 49.72 (30.39 to 75.28) | 24.22 | 25.5 | 47 (47) |
| Cambodia | 0.469 | 98.8 (65.78 to 145.73) | 24.22 | 74.58 | 166 (166) |
| Cameroon | 0.49 | 59.15 (35.04 to 92.87) | 24.21 | 34.94 | 67 (67) |
| Canada | 0.873 | 69.34 (55.59 to 85.35) | 24.2 | 45.14 | 96 (96) |
| Cape Verde | 0.525 | 48.29 (31.38 to 72.78) | 24.2 | 24.09 | 41 (40) |
| Central African Republic | 0.274 | 46.91 (27.36 to 74.96) | 25.42 | 21.49 | 33 (37) |
| Chad | 0.238 | 39.07 (24.68 to 57.85) | 25.32 | 13.75 | 17 (18) |
| Chile | 0.759 | 66.31 (53.11 to 81.64) | 24.19 | 42.12 | 86 (86) |
| China | 0.686 | 110.29 (92.57 to 130.86) | 24.2 | 86.09 | 183 (183) |
| Colombia | 0.633 | 73.7 (51.03 to 103.89) | 24.21 | 49.49 | 114 (114) |
| Comoros | 0.455 | 55.43 (29.7 to 86.95) | 24.22 | 31.21 | 58 (58) |
| Congo (Brazzaville) | 0.568 | 68.81 (39.61 to 108.4) | 24.21 | 44.6 | 95 (95) |
| Cook Islands | 0.764 | 50.7 (26.89 to 77.87) | 24.21 | 26.49 | 49 (49) |
| Costa Rica | 0.68 | 101.12 (71.33 to 140.17) | 24.2 | 76.92 | 171 (171) |
| Croatia | 0.794 | 90.93 (63.53 to 126.38) | 24.18 | 66.75 | 154 (154) |
| Cuba | 0.668 | 73.75 (54.08 to 98.46) | 24.21 | 49.54 | 115 (116) |
| Cyprus | 0.841 | 45.28 (33.19 to 59.95) | 24.21 | 21.07 | 32 (32) |
| Denmark | 0.89 | 63.36 (50.92 to 77.95) | 24.21 | 39.15 | 80 (80) |
| Djibouti | 0.459 | 68.1 (37.12 to 112.1) | 24.21 | 43.89 | 94 (94) |
| Dominica | 0.729 | 81.16 (54.52 to 116.81) | 24.21 | 56.95 | 140 (140) |
| Dominican Republic | 0.592 | 92.12 (57.61 to 138.05) | 24.2 | 67.92 | 157 (157) |
| DR Congo | 0.382 | 36.58 (21.4 to 58.93) | 24.21 | 12.37 | 16 (15) |
| Ecuador | 0.64 | 73.11 (50.62 to 103.2) | 24.21 | 48.9 | 112 (112) |
| Egypt | 0.658 | 59.57 (36.98 to 89.1) | 24.22 | 35.35 | 70 (71) |
| El Salvador | 0.573 | 79.08 (52.48 to 115.16) | 24.21 | 54.87 | 135 (135) |
| Equatorial Guinea | 0.685 | 74.9 (37.54 to 132.53) | 24.2 | 50.7 | 120 (120) |
| Eritrea | 0.396 | 73.66 (45.27 to 113.81) | 24.18 | 49.48 | 113 (113) |
| Estonia | 0.835 | 71.75 (49.36 to 101.2) | 24.18 | 47.57 | 108 (107.5) |
| eSwatini | 0.577 | 89.98 (47.72 to 152.27) | 24.19 | 65.79 | 150 (150) |
| Ethiopia | 0.343 | 48.94 (34.49 to 69.92) | 24.21 | 24.73 | 44 (44) |
| Federated States of Micronesia | 0.58 | 109.15 (32.17 to 182.34) | 24.21 | 84.94 | 181 (181) |
| Fiji | 0.664 | 76.79 (50.82 to 111.85) | 24.15 | 52.64 | 125 (125) |
| Finland | 0.856 | 46.12 (36.3 to 57.76) | 24.22 | 21.9 | 35 (35) |
| France | 0.834 | 60.1 (48.24 to 73.56) | 24.19 | 35.91 | 74 (74) |
| Gabon | 0.656 | 82.3 (47.78 to 130.89) | 24.21 | 58.09 | 142 (142) |
| Georgia | 0.702 | 74.35 (53.88 to 99.9) | 24.2 | 50.15 | 118 (118.5) |
| Georgia | 0.841 | 74.35 (53.88 to 99.9) | 24.2 | 50.15 | 119 (118.5) |
| Germany | 0.898 | 65.86 (53.54 to 79.78) | 24.21 | 41.65 | 85 (85) |
| Ghana | 0.557 | 57.25 (35.64 to 86.28) | 24.16 | 33.09 | 62 (62) |
| Greece | 0.794 | 54.71 (45.79 to 64.89) | 24.21 | 30.5 | 57 (57) |
| Greenland | 0.761 | 152.47 (105.62 to 218.69) | 24.21 | 128.26 | 201 (201) |
| Grenada | 0.669 | 95.48 (69.3 to 126.93) | 24.17 | 71.31 | 162 (162) |
| Guam | 0.813 | 126.91 (86.23 to 181.33) | 24.21 | 102.7 | 195 (195) |
| Guatemala | 0.526 | 74.25 (53.69 to 99.7) | 24.18 | 50.07 | 117 (117) |
| Guinea | 0.325 | 40.34 (25.49 to 61.04) | 24.21 | 16.13 | 21 (21) |
| Guinea-Bissau | 0.355 | 58.64 (36.94 to 86.64) | 24.2 | 34.44 | 65 (65.5) |
| Guyana | 0.618 | 115.1 (76.42 to 166.02) | 24.21 | 90.89 | 189 (189) |
| Haiti | 0.432 | 67.03 (39.18 to 104.87) | 24.2 | 42.83 | 89 (89) |
| Honduras | 0.496 | 41.43 (22.74 to 69.52) | 24.19 | 17.24 | 22 (22) |
| Hungary | 0.791 | 108.66 (80.94 to 144.32) | 24.19 | 84.47 | 180 (180) |
| Iceland | 0.869 | 43.12 (33.11 to 55.02) | 24.14 | 18.98 | 24 (24) |
| India | 0.566 | 50 (41.06 to 60.23) | 24.21 | 25.79 | 48 (48) |
| Indonesia | 0.66 | 100.28 (70.86 to 133.09) | 24.21 | 76.07 | 170 (170) |
| Iran | 0.67 | 59.69 (54.14 to 66.28) | 24.18 | 35.51 | 72 (72) |
| Iraq | 0.671 | 63.17 (41.33 to 94.72) | 24.19 | 38.98 | 79 (79) |
| Ireland | 0.867 | 56.53 (43.89 to 71.68) | 24.2 | 32.33 | 61 (61) |
| Israel | 0.803 | 64.97 (52.09 to 80.43) | 24.21 | 40.76 | 83 (83) |
| Italy | 0.801 | 59.56 (54.87 to 64.18) | 24.2 | 35.36 | 71 (70) |
| Jamaica | 0.684 | 101.16 (68.78 to 142.34) | 24.21 | 76.95 | 172 (172) |
| Japan | 0.87 | 71.39 (66.54 to 75.34) | 24.15 | 47.24 | 106 (106) |
| Jordan | 0.731 | 70.19 (50.76 to 95.33) | 24.18 | 46.01 | 99 (99) |
| Kazakhstan | 0.723 | 70.69 (55.43 to 88.6) | 24.19 | 46.5 | 102 (102) |
| Kenya | 0.508 | 48.79 (36.43 to 63.37) | 24.21 | 24.58 | 43 (42) |
| Kiribati | 0.527 | 105.55 (66.43 to 164.09) | 24.18 | 81.37 | 178 (178) |
| Kuwait | 0.851 | 35.29 (25.49 to 47.61) | 24.2 | 11.09 | 12 (11) |
| Kyrgyzstan | 0.596 | 49.18 (37 to 63.91) | 24.2 | 24.98 | 45 (45) |
| Laos | 0.49 | 102.08 (59.89 to 155.21) | 24.15 | 77.93 | 175 (175) |
| Latvia | 0.82 | 70.43 (50.49 to 96.13) | 24.15 | 46.28 | 100 (100) |
| Lebanon | 0.708 | 88.76 (58.5 to 127.36) | 24.21 | 64.55 | 147 (147) |
| Lesotho | 0.507 | 75.32 (43.05 to 117.72) | 24.18 | 51.14 | 123 (123) |
| Liberia | 0.37 | 34.73 (19.5 to 56.24) | 24.18 | 10.55 | 9 (8) |
| Libya | 0.709 | 90.19 (58.11 to 134.96) | 24.21 | 65.98 | 151 (151) |
| Lithuania | 0.843 | 72.95 (52.84 to 98.92) | 24.21 | 48.74 | 111 (111) |
| Luxembourg | 0.895 | 52.41 (41.74 to 64.97) | 24.18 | 28.23 | 53 (53) |
| Madagascar | 0.396 | 51.53 (32.17 to 77.54) | 24.18 | 27.35 | 51 (51.5) |
| Malawi | 0.384 | 34.66 (20.79 to 54.7) | 24.23 | 10.43 | 8 (7) |
| Malaysia | 0.737 | 96.94 (64.57 to 139.78) | 24.21 | 72.73 | 163 (163) |
| Maldives | 0.562 | 35.77 (24.38 to 50.76) | 24.19 | 11.58 | 14 (13) |
| Mali | 0.263 | 44.9 (27.87 to 67.65) | 25.31 | 19.59 | 27 (31) |
| Malta | 0.801 | 52.47 (39.57 to 68.21) | 24.21 | 28.26 | 54 (54) |
| Marshall Islands | 0.544 | 109.34 (65.19 to 167.82) | 24.21 | 85.13 | 182 (182) |
| Mauritania | 0.496 | 35.59 (20.61 to 56.2) | 24.2 | 11.39 | 13 (12) |
| Mauritius | 0.705 | 80.15 (58.95 to 107.73) | 24.22 | 55.93 | 137 (137) |
| Mexico | 0.649 | 72.13 (61.07 to 84.42) | 24.19 | 47.94 | 109 (109) |
| Moldova | 0.696 | 98.69 (76.49 to 124.3) | 24.21 | 74.48 | 165 (165) |
| Monaco | 0.902 | 97.31 (63.79 to 143.07) | 24.21 | 73.1 | 164 (164) |
| Mongolia | 0.606 | 69.93 (46.06 to 103.16) | 24.21 | 45.72 | 97 (97) |
| Montenegro | 0.791 | 72.77 (53.82 to 95.49) | 24.18 | 48.59 | 110 (110) |
| Morocco | 0.548 | 46.06 (28.69 to 73.35) | 24.14 | 21.92 | 36 (34) |
| Mozambique | 0.307 | 47.98 (28 to 75) | 24.21 | 23.77 | 40 (39) |
| Myanmar | 0.521 | 99.82 (62.12 to 151.21) | 24.21 | 75.61 | 168 (168) |
| Namibia | 0.612 | 46.04 (26.45 to 73.94) | 24.26 | 21.78 | 34 (33) |
| Nauru | 0.618 | 152.91 (82.76 to 235.38) | 24.19 | 128.72 | 202 (202) |
| Nepal | 0.422 | 30.35 (17.45 to 48.11) | 24.2 | 6.15 | 4 (3) |
| Netherlands | 0.883 | 70.07 (56.33 to 85.44) | 24.21 | 45.86 | 98 (98) |
| New Zealand | 0.84 | 85.97 (70.86 to 103.66) | 24.18 | 61.79 | 144 (144) |
| Nicaragua | 0.517 | 51.52 (35.9 to 71.84) | 24.15 | 27.37 | 52 (50) |
| Niger | 0.162 | 29.19 (17.75 to 46.04) | 26.54 | 2.65 | 1 (2) |
| Nigeria | 0.515 | 34.54 (23.78 to 49.01) | 24.2 | 10.34 | 7 (6) |
| Niue | 0.711 | 93.54 (47.9 to 158.11) | 24.21 | 69.33 | 158 (158) |
| North Korea | 0.558 | 108.36 (61.67 to 179.84) | 24.22 | 84.14 | 179 (179) |
| North Macedonia | 0.744 | 101.31 (72.4 to 139.42) | 24.21 | 77.1 | 173 (173) |
| Northern Mariana Islands | 0.771 | 118.55 (74.76 to 181.16) | 24.2 | 94.35 | 192 (192) |
| Norway | 0.913 | 64.74 (57.92 to 70.64) | 24.21 | 40.53 | 82 (82) |
| Oman | 0.783 | 32.26 (20.94 to 51.75) | 24.2 | 8.06 | 5 (4) |
| Pakistan | 0.449 | 67.7 (47.95 to 93.99) | 24.21 | 43.49 | 92 (92) |
| Palau | 0.738 | 76.93 (48.92 to 114.64) | 24.18 | 52.75 | 126 (126) |
| Palestine | 0.588 | 99.89 (73.95 to 131.77) | 24.21 | 75.68 | 169 (169) |
| Panama | 0.686 | 66.49 (44.69 to 94.12) | 24.21 | 42.28 | 87 (87) |
| Papua New Guinea | 0.394 | 59.4 (36.81 to 91.78) | 24.2 | 35.2 | 68 (68) |
| Paraguay | 0.638 | 77.37 (50.87 to 113.27) | 24.2 | 53.17 | 129 (129) |
| Peru | 0.648 | 56.36 (36.09 to 82.94) | 24.17 | 32.19 | 60 (60) |
| Philippines | 0.623 | 136.45 (110.5 to 169.07) | 24.2 | 112.25 | 199 (199) |
| Poland | 0.802 | 87.08 (71.59 to 104.2) | 24.2 | 62.88 | 145 (145) |
| Portugal | 0.743 | 88.6 (71.05 to 109.29) | 24.17 | 64.43 | 146 (146) |
| Puerto Rico | 0.814 | 94.95 (65.4 to 135.71) | 24.2 | 70.75 | 160 (160) |
| Qatar | 0.83 | 35.03 (22.84 to 51.95) | 24.21 | 10.82 | 10 (9) |
| Romania | 0.76 | 111.31 (83.09 to 145.76) | 24.17 | 87.14 | 184 (184) |
| Russia | 0.805 | 95.09 (80.01 to 111.22) | 24.15 | 70.94 | 161 (161) |
| Rwanda | 0.429 | 56.06 (34.14 to 87.99) | 24.21 | 31.85 | 59 (59) |
| Saint Kitts and Nevis | 0.746 | 67.06 (20.7 to 110.13) | 24.21 | 42.85 | 90 (90) |
| Saint Lucia | 0.67 | 73.73 (55.98 to 95.47) | 24.15 | 49.58 | 116 (115) |
| Saint Vincent and the Grenadines | 0.627 | 90.49 (70.17 to 115.32) | 24.21 | 66.28 | 153 (153) |
| Samoa | 0.641 | 66.61 (36.69 to 103.26) | 24.21 | 42.4 | 88 (88) |
| San Marino | 0.884 | 70.59 (39.79 to 118.52) | 24.2 | 46.39 | 101 (101) |
| Saudi Arabia | 0.805 | 67.5 (43.86 to 101.01) | 24.21 | 43.29 | 91 (91) |
| Senegal | 0.389 | 44 (26.75 to 67.04) | 24.22 | 19.78 | 28 (27) |
| Serbia | 0.767 | 112.52 (79.36 to 156.72) | 24.2 | 88.32 | 187 (187) |
| Seychelles | 0.724 | 158.91 (114.48 to 215.84) | 24.2 | 134.71 | 203 (203) |
| Sierra Leone | 0.347 | 37.98 (23.51 to 57.34) | 24.16 | 13.82 | 18 (17) |
| Singapore | 0.861 | 47.26 (37.51 to 58.52) | 24.21 | 23.05 | 38 (38) |
| Slovakia | 0.812 | 98.9 (67.7 to 139.03) | 24.22 | 74.68 | 167 (167) |
| Slovenia | 0.84 | 64.24 (44.31 to 91.41) | 24.19 | 40.05 | 81 (81) |
| Solomon Islands | 0.407 | 119.95 (61.25 to 186.97) | 24.15 | 95.8 | 193 (193) |
| Somalia | 0.081 | 36.83 (20.09 to 71.93) | 32.27 | 4.56 | 3 (16) |
| South Africa | 0.678 | 62.38 (44.61 to 84.35) | 24.2 | 38.18 | 78 (78) |
| South Korea | 0.878 | 60.57 (47.6 to 75.92) | 24.21 | 36.36 | 76 (76) |
| South Sudan | 0.363 | 58.16 (31.59 to 100.52) | 24.21 | 33.95 | 64 (64) |
| Spain | 0.767 | 65.1 (53.17 to 79.52) | 24.2 | 40.9 | 84 (84) |
| Sri Lanka | 0.69 | 36.18 (23.53 to 53.75) | 24.21 | 11.97 | 15 (14) |
| Sudan | 0.515 | 49.46 (26.72 to 81.91) | 24.2 | 25.26 | 46 (46) |
| Suriname | 0.636 | 103.91 (72.13 to 144.58) | 24.15 | 79.76 | 176 (176) |
| Sweden | 0.872 | 60.92 (51.46 to 71.79) | 24.21 | 36.71 | 77 (77) |
| Switzerland | 0.929 | 44.4 (35.1 to 55.12) | 24.13 | 20.27 | 29 (28) |
| Syria | 0.619 | 44.47 (29.37 to 64.96) | 24.15 | 20.32 | 30 (29) |
| Taiwan (province of China) | 0.868 | 173.31 (124.55 to 236.4) | 24.19 | 149.12 | 204 (204) |
| Tajikistan | 0.539 | 77.8 (53.25 to 110.7) | 24.2 | 53.6 | 131 (131) |
| Tanzania | 0.423 | 59.96 (37.03 to 93.44) | 24.17 | 35.79 | 73 (73) |
| Thailand | 0.687 | 81.14 (53.99 to 117.56) | 24.22 | 56.92 | 139 (139) |
| The Bahamas | 0.796 | 134.37 (98.71 to 182.03) | 24.21 | 110.16 | 197 (197) |
| The Gambia | 0.399 | 33.53 (19.89 to 52.26) | 24.2 | 9.33 | 6 (5) |
| Timor-Leste | 0.514 | 78.22 (20.07 to 125) | 24.21 | 54.01 | 132 (132) |
| Togo | 0.417 | 43.32 (25.49 to 67.33) | 24.2 | 19.12 | 25 (25) |
| Tokelau | 0.626 | 80.69 (42.56 to 129.86) | 24.2 | 56.49 | 138 (138) |
| Tonga | 0.636 | 41.47 (26.1 to 62.41) | 24.21 | 17.26 | 23 (23) |
| Trinidad and Tobago | 0.757 | 91.3 (61.16 to 130.18) | 24.2 | 67.1 | 155 (155) |
| Tunisia | 0.672 | 51.53 (32.46 to 76.19) | 24.21 | 27.32 | 50 (51.5) |
| Turkey | 0.748 | 76.96 (55.49 to 104.16) | 24.15 | 52.81 | 128 (127.5) |
| Turkmenistan | 0.67 | 60.19 (43.61 to 81.83) | 24.2 | 35.99 | 75 (75) |
| Tuvalu | 0.589 | 91.9 (54 to 143.49) | 24.12 | 67.78 | 156 (156) |
| Uganda | 0.404 | 76.68 (48.88 to 114.71) | 24.21 | 52.47 | 124 (124) |
| UK | 0.847 | 71.75 (68.72 to 75.08) | 24.2 | 47.55 | 107 (107.5) |
| Ukraine | 0.736 | 135.64 (102.75 to 176.59) | 24.21 | 111.43 | 198 (198) |
| United Arab Emirates | 0.88 | 54.05 (33.05 to 83.5) | 24.21 | 29.84 | 55 (55.5) |
| Uruguay | 0.697 | 111.48 (88.09 to 138.6) | 24.19 | 87.29 | 185 (185) |
| USA | 0.859 | 89.35 (84.34 to 95.11) | 24.2 | 65.15 | 149 (149) |
| Uzbekistan | 0.631 | 67.74 (52.65 to 85.96) | 24.17 | 43.57 | 93 (93) |
| Vanuatu | 0.485 | 80.01 (45.06 to 123.78) | 24.16 | 55.85 | 136 (136) |
| Venezuela | 0.607 | 75.14 (52.24 to 105.1) | 24.19 | 50.95 | 121 (121) |
| Vietnam | 0.617 | 101.96 (65.89 to 151.68) | 24.21 | 77.75 | 174 (174) |
| Virgin Islands | 0.799 | 125.6 (80.42 to 194.59) | 24.22 | 101.38 | 194 (194) |
| Yemen | 0.412 | 46.36 (27.12 to 72.09) | 24.19 | 22.17 | 37 (36) |
| Zambia | 0.505 | 90.31 (54.15 to 138.15) | 24.19 | 66.12 | 152 (152) |
| Zimbabwe | 0.476 | 88.78 (54.05 to 135.1) | 24.21 | 64.57 | 148 (148) |

SDI: Socio-demographic index; UI: uncertainty interval; DALYs, Disability-Adjusted Life Years.
